# Pde1b controls cyclic nucleotide signaling in cDC1 and macrophages to regulate immune responses

**DOI:** 10.1038/s44319-026-00924-z

**Published:** 2026-09-15

**Authors:** Lais L O Rekowsky, Ricardo L L da Silva, Jonathan A Seenarine, Ayane de Sa Resende, Marissa Macchietto, Tatiana R de Moura, Diana M Elizondo, Michael W Lipscomb

**Affiliations:** 1 Department of Pharmacology, University of Minnesota, Minneapolis, MN 55455 USA; 2 Department of Health Education, Universidade Federal de Sergipe, Campus Lagarto, Lagarto, Sergipe CEP 49400-000 Brazil; 3 University of Minnesota Supercomputing Institute (MSCI), University of Minnesota, Minneapolis, MN 55455 USA; 4 Laboratory of Immunology and Molecular Biology, Federal University of Sergipe, Aracaju, Sergipe 49060-108 Brazil; 5 Department of Biological Sciences, University of Maryland Baltimore County, Baltimore, MD 21250 USA; 6 Center for Immunology, University of Minnesota, Minneapolis, MN 55455 USA

**Keywords:** Immunology, Microbiology, Virology & Host Pathogen Interaction, Signal Transduction

## Abstract

Cyclic nucleotides cAMP and cGMP are potent suppressors of immune activation, yet how their intracellular levels are regulated within antigen-presenting myeloid cells remains undefined. Here we identify phosphodiesterase 1B (Pde1b) as a lineage-restricted regulator of cyclic nucleotide signaling in conventional type 1 dendritic cells (cDC1) and a subset of macrophages. Using genetic loss-of-function models and Listeria infection, we show that Pde1b is required for immune responses. Pde1b deficiency impairs bacterial clearance and reduces antigen-specific CD8+ and CD4 + T cell expansion, while myeloid transcriptional programs are skewed towards alternative and immunoregulatory states. These defects are largely attributable to DC dysfunction, as adoptive transfer of wild-type DC in Pde1b-deficient recipients partially rescues T cell priming. Macrophages exhibit parallel defects, including reduced frequency and impaired inflammatory and antimicrobial function. Mechanistically, Pde1b loss elevates cAMP and cGMP in cDC1 and macrophages, leading to increased PKA/PKG signaling and CREB-associated transcriptional reprogramming. Pharmacological inhibition of other phosphodiesterase families does not fully recapitulate Pde1b loss. Together, these findings identify Pde1b as a cell-type-specific regulator of cyclic nucleotide signaling essential for immune responses.

## Synopsis

Phosphodiesterase 1B (Pde1b) is a lineage-restricted regulator of cyclic nucleotides in cDC1 and macrophages. Its deficiency impairs cyclic nucleotide homeostasis, attenuating antibacterial immunity and T cell priming.

- Pde1b is selectively expressed in cDC1 and specific macrophage subsets to control intracellular cAMP and cGMP levels.
- Loss of Pde1b disrupts antibacterial defense and significantly reduces antigen-specific CD8+ and CD4 + T cell priming in vivo.
- Pde1b deficiency drives aberrant transcriptional reprogramming and promotes an immunosuppressive state within myeloid populations.

## Abstract (web summary)

Phosphodiesterase 1B (Pde1b) is a lineage-restricted regulator of cyclic nucleotides in cDC1 and macrophages. Its deficiency impairs cyclic nucleotide homeostasis, attenuating antibacterial immunity and T cell priming.

## Graphical abstract

**Figure:**
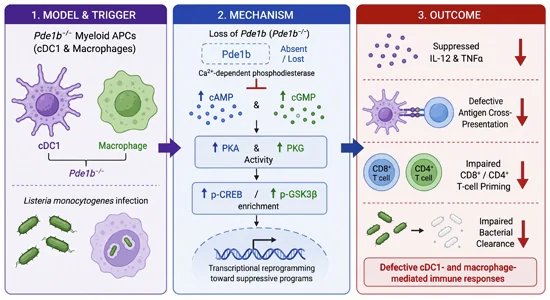

## Introduction

Phosphodiesterase (PDE) enzymes comprise 11 gene families that have different tissue expression patterns, regulatory modalities, specificities and rate kinetics for hydrolyzing cyclic nucleotides. Within this family, calcium/calmodulin-dependent PDE enzymes modulate intracellular cyclic AMP (cAMP) and cyclic GMP (cGMP) levels by selective hydrolysis, thereby controlling activation of protein kinases such as protein kinase A (PKA) and protein kinase G (PKG) (Erneux et al, 1985). These protein kinases can regulate downstream signaling activities involving  cAMP response element binding (CREB), glycogen synthase kinase 3 (GSK3), nuclear factor-kappa B (NFκB) and protein phosphatase 2A (PP2A) proteins, among others, which collectively shape immune and inflammatory responses (Kong et al, 2004; Montminy and Bilezikjian, 1987; Xie et al, 2013). This mechanistic interplay between phosphodiesterase enzymes and intracellular signaling is a recurrent theme across cell types, underpinning core homeostatic processes.

In the immune system, elevated intracellular levels of cAMP and cGMP are well-recognized to exert potent immunosuppressive and anti-inflammatory effects. More recent evidence demonstrates that activation of cAMP-dependent PKA and cGMP-dependent PKG pathways attenuates myeloid antigen-presenting cell functions and shifts T helper cell polarization toward a T_H_2 or T_H_17 phenotype (Abrahamsen et al, 2004; Aronoff et al, 2005; Galgani et al, 2004; Lee et al, 2020; Lee et al, 2015; Li et al, 1999). Additionally, regulatory T cells (Tregs) utilize cAMP to enforce tolerance by elevating cyclic nucleotide levels in neighboring myeloid and lymphoid cells to restrain immune responses (Bopp et al, 2007; Huang et al, 2009; Feng et al, 2011; Ring et al, 2015; Vang et al, 2013).

Dendritic cells (DC) and macrophages play pivotal roles as myeloid antigen-presenting cells (APC). DC comprise heterogeneous subsets with specialized functional roles, including conventional type 1 dendritic cells (cDC1), which are specialized for cross-presentation to CD8 + T cells and promotion of type 1 immune responses, and conventional type 2 dendritic cells (cDC2), which are associated with CD4 + T cell priming and the induction of Th2- and Th17-polarized responses that support humoral and barrier immunity. A third plasmacytoid DC (pDC) population contributes primarily to antiviral interferon responses. Similarly, macrophages exhibit classically activated (M1-like, pro-inflammatory) and alternatively activated (M2-like, anti-inflammatory/repair-associated) states shaped by microenvironmental cues. Collectively, these APC subsets can thus integrate complex intracellular signaling programs to coordinate distinct innate and adaptive immune responses in defense of pathogens and transformed cells.

Cyclic nucleotides critically influence both the differentiation and function of these DC and macrophage subsets (Giordano et al, 2003; Luan et al, 2015; Negreiros-Lima et al, 2020; Ottonello et al, 1995; Zhu et al, 2008). Elevated intracellular levels of cyclic nucleotides can impair differentiation and antigen presentation capacities, while also inhibiting phagocytic and inflammatory functions (Aronoff et al, 2005; Bryn et al, 2006; Challier et al, 2013; Giordano et al, 2003; Kambayashi et al, 2001; Li et al, 2008). High levels of cAMP in cDC1 suppress IRF8-dependent transcriptional programs and restrain type 1-polarized immune responses (la Sala et al, 2009), with elevated cGMP levels in DC skewing T cell priming toward a T_H_2-promoting phenotype, coupled with depressed levels of IL-12 and TNFɑ expression (Gnipp et al, 2018; Morita et al, 2003).

Phosphodiesterase 1B (Pde1b) is a calcium/calmodulin-regulated dual-specificity hydrolase that degrades both cAMP and cGMP (Bender and Beavo, 2006; Reed et al, 1998; Yu et al, 1997). Seminal work has established that Pde1b expression rises during macrophage differentiation from monocyte precursors and supports terminal differentiation by lowering cyclic nucleotide levels (Bender and Beavo, 2006; Bender et al, 2004; Bender et al, 2005). While prior studies first highlighted Pde1b’s potential as an immunoregulator, the detailed systems-level roles of Pde1b across immune compartments and the mechanisms by which it regulates integrated signaling axes to govern antigen-specific immunity have not been explored in vivo.

Here, we address this gap by combining in vivo pathogen challenge models with antigen-specific T cell readouts, rescue/add-back studies, single-cell transcriptomics, and multi-parameter flow cytometry in a Pde1b-deficient mouse model. The approach has allowed us to uncover its critical immunomodulatory roles. Our analyses reveal that Pde1b expression is restricted to specific antigen-presenting myeloid subsets, particularly cDC1 and a distinct macrophage subpopulation, and is absent in lymphoid cells. Notably, Pde1b deficiency leads to impaired inflammatory profiles and restrained antigen-specific T cell responses, indicating disruption of both myeloid activation programs and downstream adaptive immune responses.

Mechanistically, Pde1b deficiency drives elevated intracellular cAMP and cGMP levels, resulting in heightened PKA and PKG activities. This dysregulation is accompanied by increased phosphorylation of GSK3β and CREB within cDC1 and macrophages, linking cyclic nucleotide control to downstream transcriptional and functional immune pathways. Collectively, our findings identify Pde1b as a lineage-restricted regulator of cyclic nucleotide second messenger signaling that shapes immune effector responses.

## Results

### Pde1b is selectively expressed in cDC1 dendritic cells, macrophages and neutrophils, but not in cDC2, B cells or T cells

**Figure EV1:**
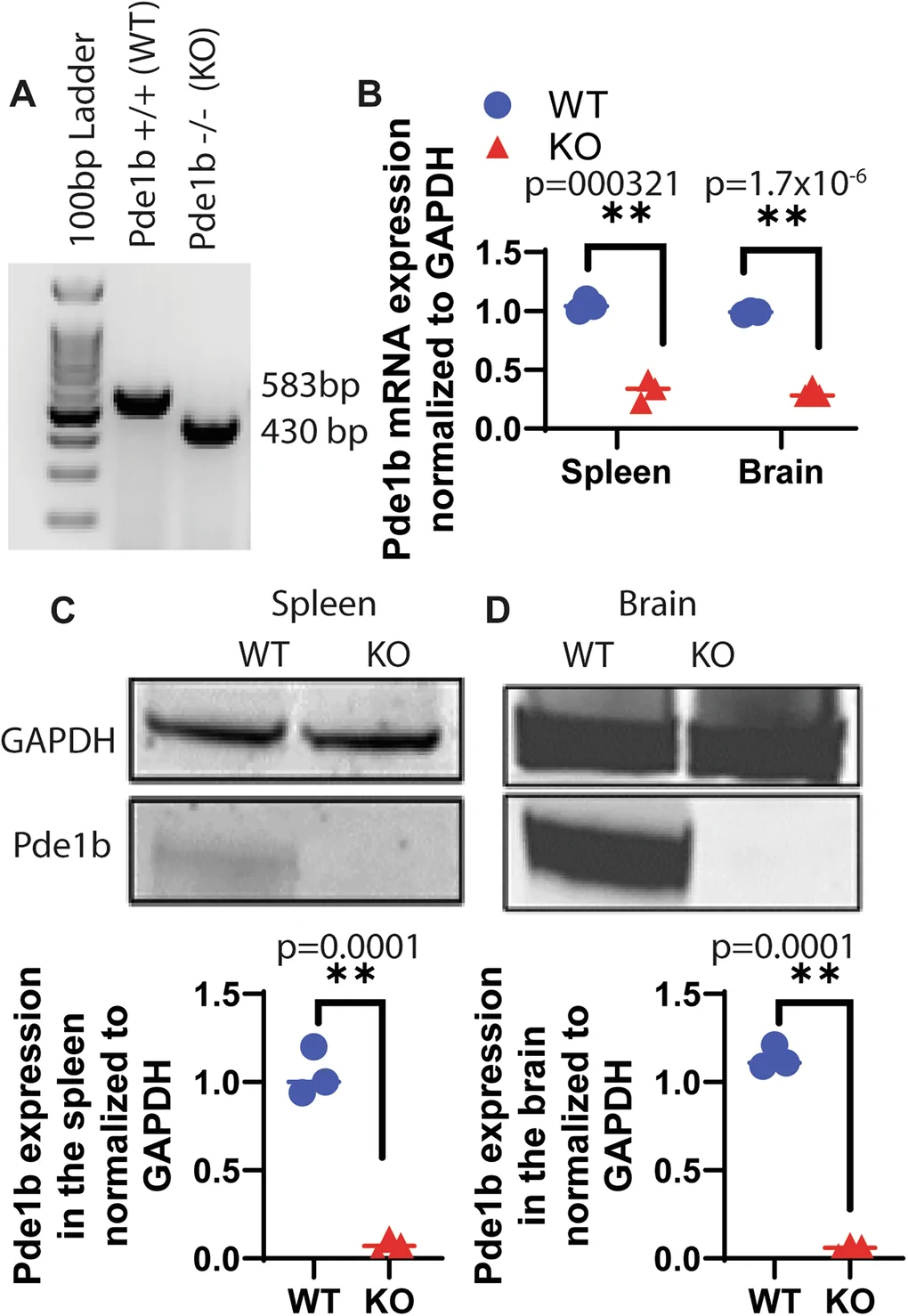
Validation of the Pde1b global knockout mouse. (**A**) Genotyping PCR of Pde1b−/− and WT mice, showing genomic DNA band sizes for the targeted Pde1b locus. (**B**) Quantitative PCR analysis of Pde1b mRNA expression in spleen and brain, normalized to beta actin. Data shown as a mean ± SEM from three biological replicates. (**C**,** D**) Western blot analysis of Pde1b protein in spleen and brain tissues from Pde1b−/− and WT mice. Protein levels were normalized to their respective endogenous control. Bar graphs show quantification of Pde1b protein expression relative to GAPDH. Data shown as a mean ± SEM from three biological replicates. Statistical analysis among group conditions was performed using an unpaired Student’s *t*-test. *p < 0.05, **p < 0.01 and ns not significant; numerical *p* values for statistically significant comparisons are shown above indicated groups within the graphs where available.

To determine the effects of Pde1b loss, global knockout mice (Pde1b−/−) were utilized. No differences in health or viability were observed among WT, Pde1b+/− and Pde1b−/− (KO) genotypes, consistent with prior reports (Reed et al, 2002). Deletion of exon 3 in the Pde1b gene generated a ~150 bp smaller band in Pde1b−/− mice compared to WT upon PCR genotyping (Fig. EV1A). This Pde1b−/− strain produces unstable transcripts as measured by qPCR (Fig. EV1B). However, no protein can be detected by Western blot (Fig. EV1C,D).

**Figure 1:**
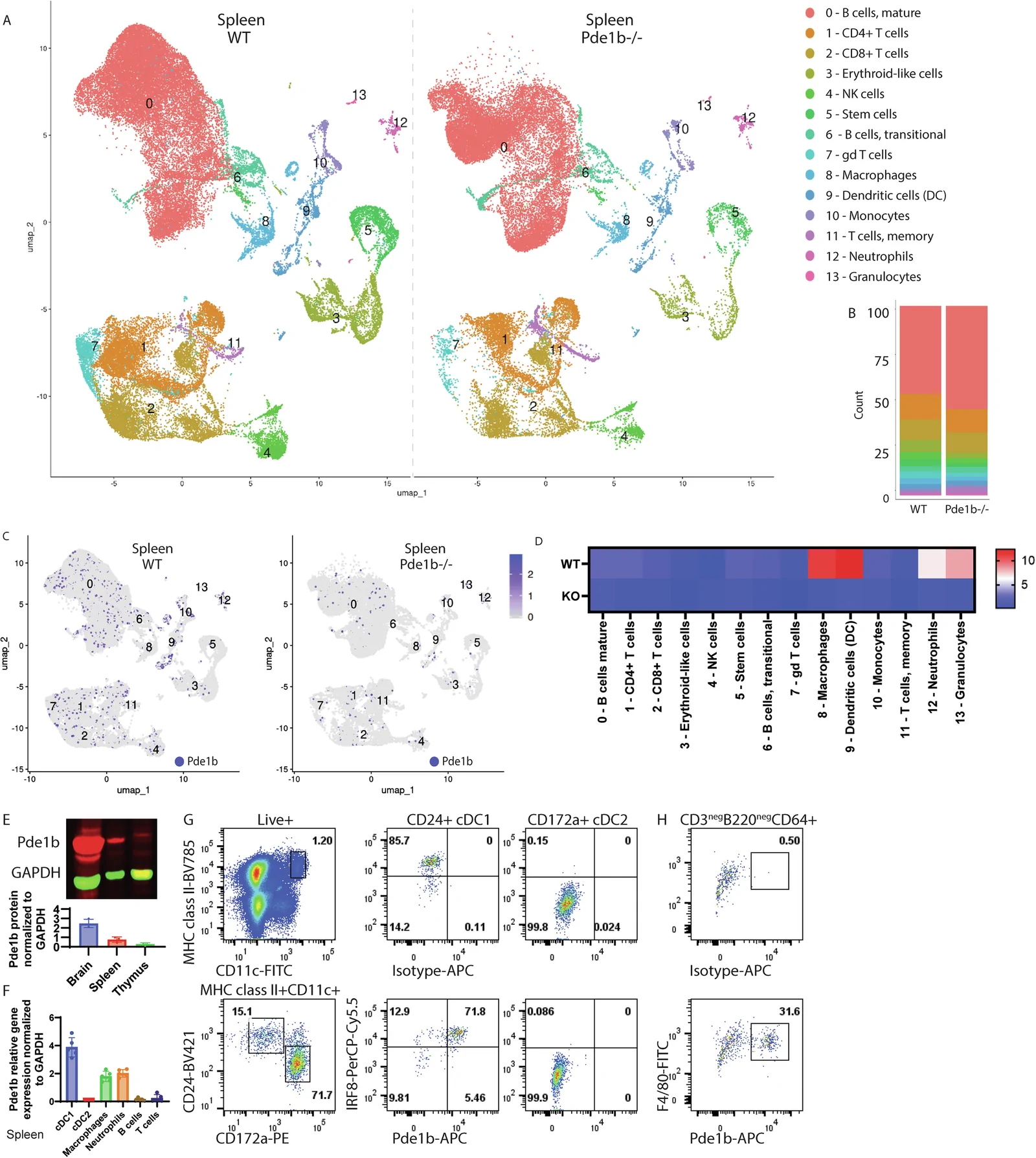
Pde1b expression and validation of genetic deletion in splenic immune populations. (**A**) UMAP projection showing distribution of immune cell subsets across genotypes based on clustering analysis of single-cell RNA sequencing (scRNA-seq) data from WT and Pde1b−/− spleens, identifying major immune cell populations. (**B**) Relative abundance of major immune cell populations. (**C**,** D**) Feature plot and quantification of Pde1b expression across immune clusters in WT versus Pde1b−/− mice. (**E**) Western blot analysis of Pde1b protein expression in brain, spleen, and thymus from WT mice. Protein levels normalized to GAPDH. (**F**) Quantitative PCR (qPCR) analysis of Pde1b mRNA expression in FACS-sorted splenic immune populations, including cDC1, cDC2, macrophages, monocytes, neutrophils, B cells, and T cells. (**G**) Flow cytometric identification of Pde1b-expressing cDC1 subsets (CD11c+MHC class II + CD24+CD172a^neg^); IRF8 expression was used to confirm lineage identity. (**H**) Flow cytometric analysis of splenic macrophages (F4/80 + CD64 + ) for Pde1b expression. Data in panels (**A**–**D**) are derived from scRNA-seq analysis of *n* = 4 mice per genotype. Data in panels (**E**–**H**) represent *n* = 3 to 6 biological replicates as indicated, shown as mean ±  SEM. Statistical analyses were performed using two-tailed unpaired Student’s *t*-tests. Exact *p* values are indicated in the figures. Statistical significance is denoted as *p < 0.05 and *p* < 0.01; ns not significant. Source data are available online for this figure.

Single-cell RNA sequencing (scRNA-seq) of splenic cells from Pde1b−/− and WT mice analyzed an average of ~25,000 high-quality cells per genotype across four biological replicates (*n* = 4 each), with rigorous batch correction, cell-type annotation and confirmation of major findings in independently sorted and flow cytometry-validated cohorts. Canonical correlation analysis and clustering identified 14 major immune cell populations (Fig. 1A). Comparing Pde1b–/− to WT controls revealed differences in the frequency and distribution of mature B cells, macrophages, memory T cells and transitional B cells (Fig. 1B). While Pde1b−/− mice showed minor detection of residual 5′ transcript fragments due to disruption of the gene by the exon 3 insertion cassette (Fig. 1C), overall detected transcript counts were reduced compared to WT. Pde1b transcript expression levels in WT confirmed a strong presence in macrophages (cluster 8) and DC (cluster 9), and to a lesser extent in neutrophils (cluster 12) and granulocytes (cluster 13), with expression largely abolished in the KO (Fig. 1D).

To corroborate gene expression profiling with protein assessment, a Western blot was performed to confirm levels in the spleen of C57BL/6 wild-type (WT) mice, with only marginal levels in the thymus (Fig. 1E); brain tissue was included as a reference sample based on prior reports of Pde1b expression to confirm expected tissue-specific expression (Reed et al, 2002). For deeper probing, FACS-sorted analysis of splenic immune populations revealed Pde1b protein expression to be in cDC1, macrophages and neutrophils, with no expression measured in cDC2 subsets, B cells or T cells, as evaluated by qPCR (Fig. 1F). Flow cytometric analysis of spleen confirmed Pde1b expression in cDC1, defined as CD24+CD172a^neg^IRF8+ within the CD11+ MHC class II+ subsets (Fig. 1G) and in the F4/80+ CD64+ macrophages (Fig. 1H).

### Pde1b is essential for effective clearance of *Listeria* bacterial infection in vivo

**Figure 2:**
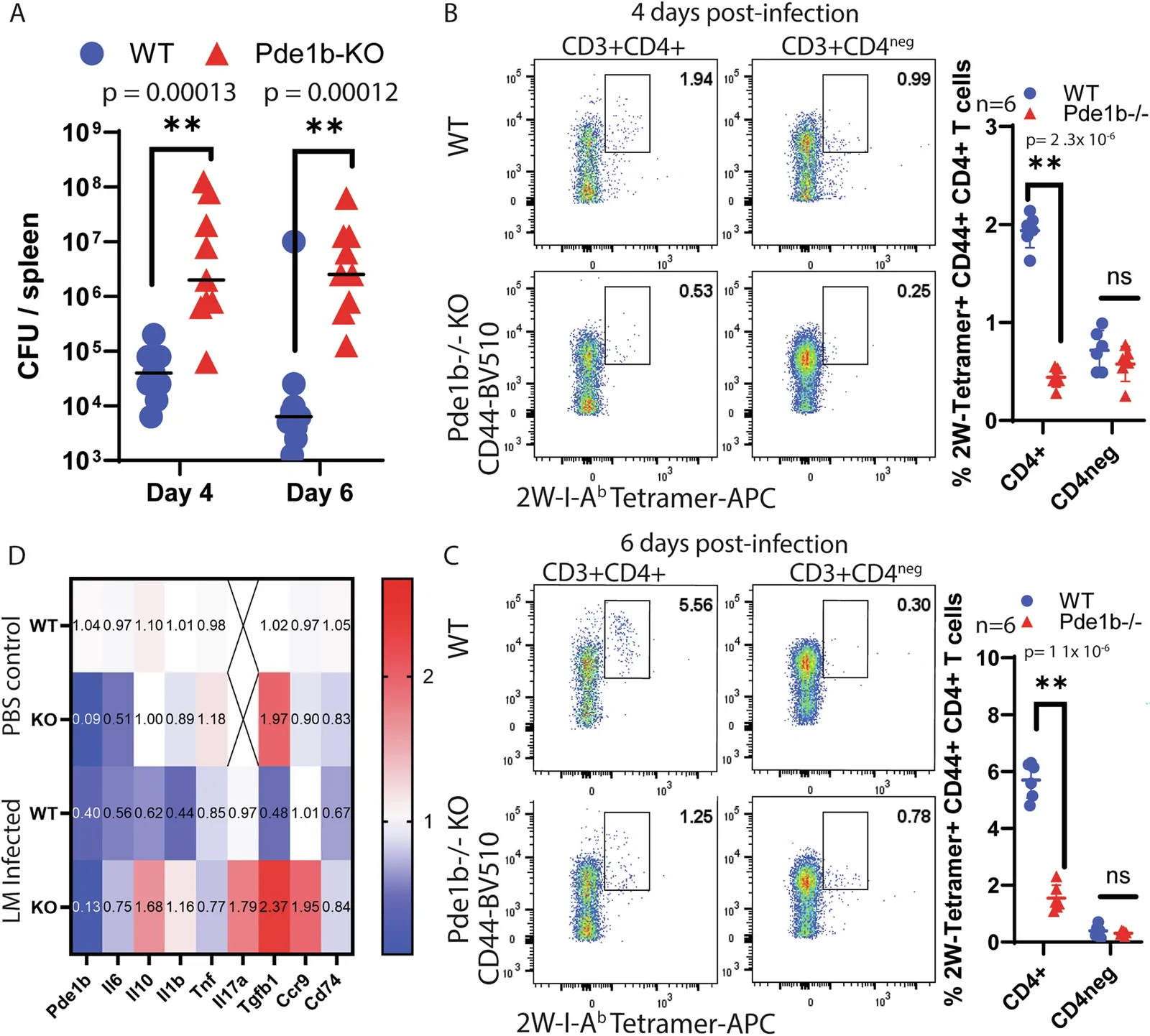
Absence of Pde1b impairs antibacterial immunity and antigen-specific CD4 + T cell responses in vivo. (**A**) Bacterial burden in spleens of WT and Pde1b−/− mice following infection with *Listeria monocytogenes* (LM) at the indicated time points. Data shown as a mean ± SEM from nine biological replicates. (**B**,** C**) Frequency of antigen-specific CD44+ CD4+ T cells following infection with attenuated ΔactA-LM-2W, measured by 2W:I-A^b^ tetramer staining at days 4 and 6 post-infection. Bar graphs show mean ± SEM from 6 biological replicates. (**D**) qPCR analysis of cytokine gene expression in spleens from uninfected mice or at day 6 post-infection in WT vs. KO groups. Statistical analyses were performed using two-tailed unpaired Student’s *t*-tests. Data were shown as mean ± SEM. Exact *p* values are indicated in the figures. Statistical significance is denoted as *p < 0.05 and **p < 0.01; ns not significant. Source data are available online for this figure.

Given select expression of Pde1b in myeloid APC populations, in vivo challenge experiments were performed to assess the capacity to mount functional immune responses against bacterial challenge. To assess bacterial burden, mice were infected with *live* sublethal levels of *Listeria monocytogenes* (LM). Bacterial burden in the spleen remained elevated in Pde1b−/− mice compared to WT controls at days 4 and 6 post-infection (Fig. 2A). To assess in vivo antigen-specific T cell responses, studies employed the attenuated strain of LM expressing the 2W antigen (ΔactA-LM-2W), which has reduced virulence but is still capable of eliciting a strong adaptive immune response that can be well quantified. Using 2W:I-Ab MHC class II tetramers, a markedly lower frequency of LM-antigen-specific CD44^hi^CD4 + T cells were measured on both days 4 and 6 post-infection in Pde1b−/− mice compared to WT controls (Fig. 2B,C). As an internal control, CD3+CD4^neg^ T cells (representing the CD8+ T cell compartment) showed no binding to the tetramer, consistent with MHC class I restriction. Assessment of total spleen by qPCR at day 6 post-infection revealed increased expression of cytokines Il10, Il17a and Tgfb1 and the chemokine Ccr9 (Fig. 2D).

### Loss of Pde1b impairs macrophage inflammatory function and cDC1 antigen presentation

**Figure 3:**
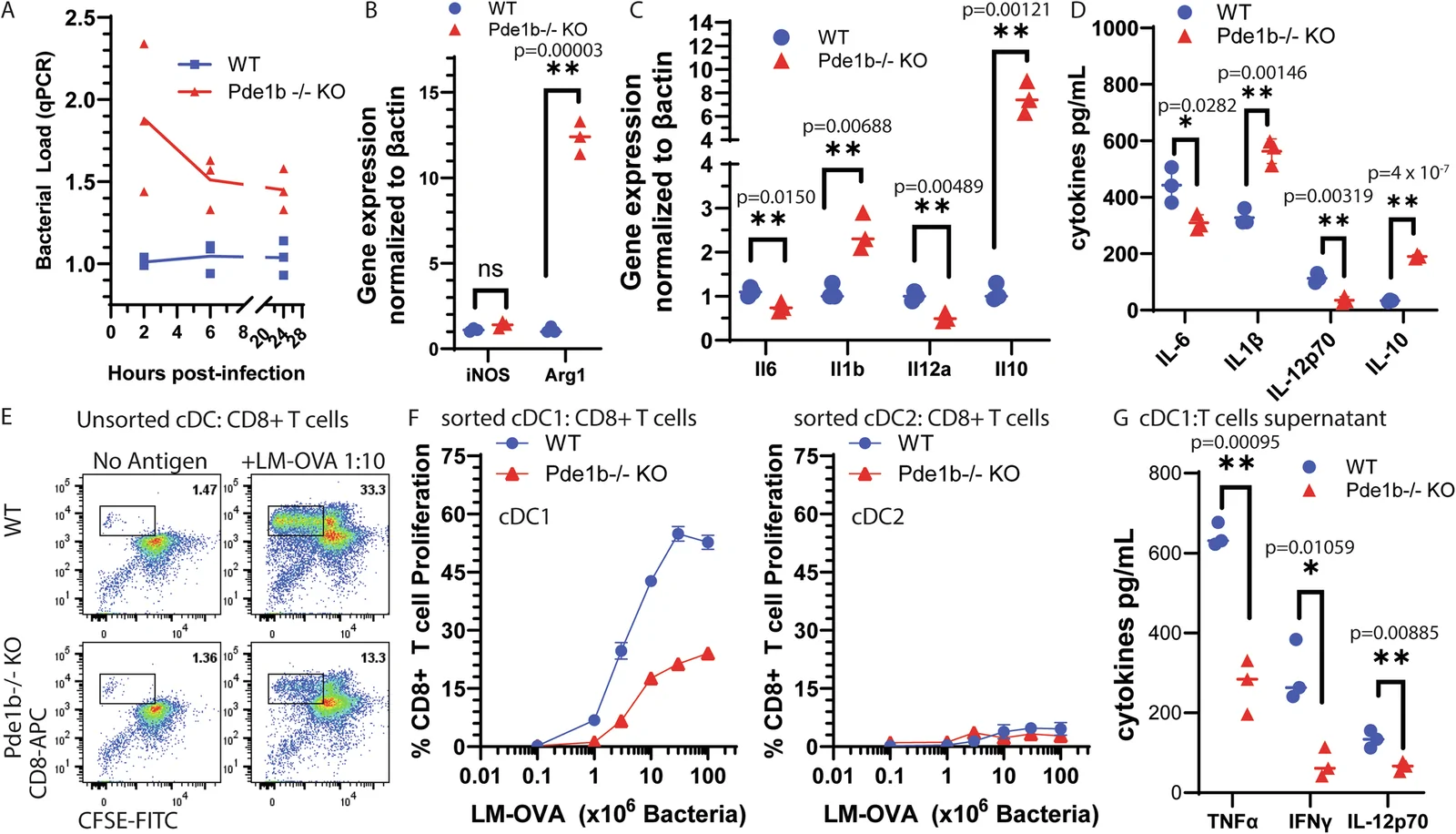
Pde1b deficiency impairs macrophage inflammatory responses and cDC1 antigen presentation. (**A**) Intracellular bacterial burden in bone marrow-derived macrophages (BMM) following LM infection over 24 h in vitro. (**B**, **C**) qPCR analysis of regulatory (Arg1, Nos2 (iNOS)) and cytokine (Il6, Il1b, Il12a, and Il10) gene expression in infected macrophages. (**D**) ELISA quantification of cytokines (IL-6, IL-1β, IL-12p70, IL-10, and TGFβ) in macrophage supernatants. (**E**) Proliferation of OT-I CD8+ T cells co-cultured with LM-OVA-infected Flt3L-derived DC. (**F**) Antigen presentation capacity of FACS-sorted cDC1 and cDC2 subsets infected with varying doses of LM-OVA. (**G**) Cytokine production (TNFα, IFNγ, IL-12p70) from cDC1:T cell cocultures. Data in panels **A** through **G** represent *n* = 3 biological replicates, shown as mean ± SEM. Statistical analyses were performed using two-tailed unpaired Student’s *t*-tests. Exact *p* values are indicated in the figures. Statistical significance is denoted as *p < 0.05 and *p* < 0.01; ns not significant. Source data are available online for this figure.

Given the reduced priming of LM-specific CD4 + T cells observed in Pde1b−/− mice following in vivo pathogen challenge, studies then investigated the specific role of macrophages and cDC1 in inflammation and adaptive immunity. Pde1b−/− bone marrow-derived macrophages showed impaired LM clearance upon infection (Fig. 3A), concomitant with elevated levels of Arg1 and Il10 and depressed expression of Il12a (Fig. 3B,C). Secreted cytokine profiles confirmed increased IL-10 and reduced IL-12p70 (Fig. 3D).

Flt3L-generated bone marrow DC (Flt3L-cDC) from Pde1b−/− mice infected with LM encoding ovalbumin (LM-OVA) had reduced capacity to stimulate proliferation of OT-I CD8+ T cells (OVA-specific T cell receptor-transgenic CD8+ T cells recognizing OVA peptide presented on MHC class I) (Fig. 3E), consistent with impaired cross-presentation capacity required for priming responses to intracellular pathogens. Given the selective expression of Pde1b in cDC1, studies next assessed whether this defect reflects a cDC1-intrinsic impairment. FACS-sorted cDC1 from the spleens of Pde1b−/− mice infected with LM-OVA at varying concentrations had reduced capacity to drive CD8+ T cell responses, whereas cDC2 had poor priming capacity regardless of genotype (Fig. 3F). Supernatants from LM-infected Pde1b−/− cDC1 and OT-I CD8+ T cell cocultures contained lower levels of TNFα, IFNγ and IL-12p70 compared to WT controls (Fig. 3G). These results demonstrate that Pde1b expression is important for effective inflammatory responses and antigen-specific T cell priming in myeloid APC.

### Rescue by adoptive transfer of WT cDC, but not Pde1b-deficient cDC, is able to partially restore priming of LM-specific CD8+ T cell responses in Pde1b−/− recipient mice in vivo

**Figure 4:**
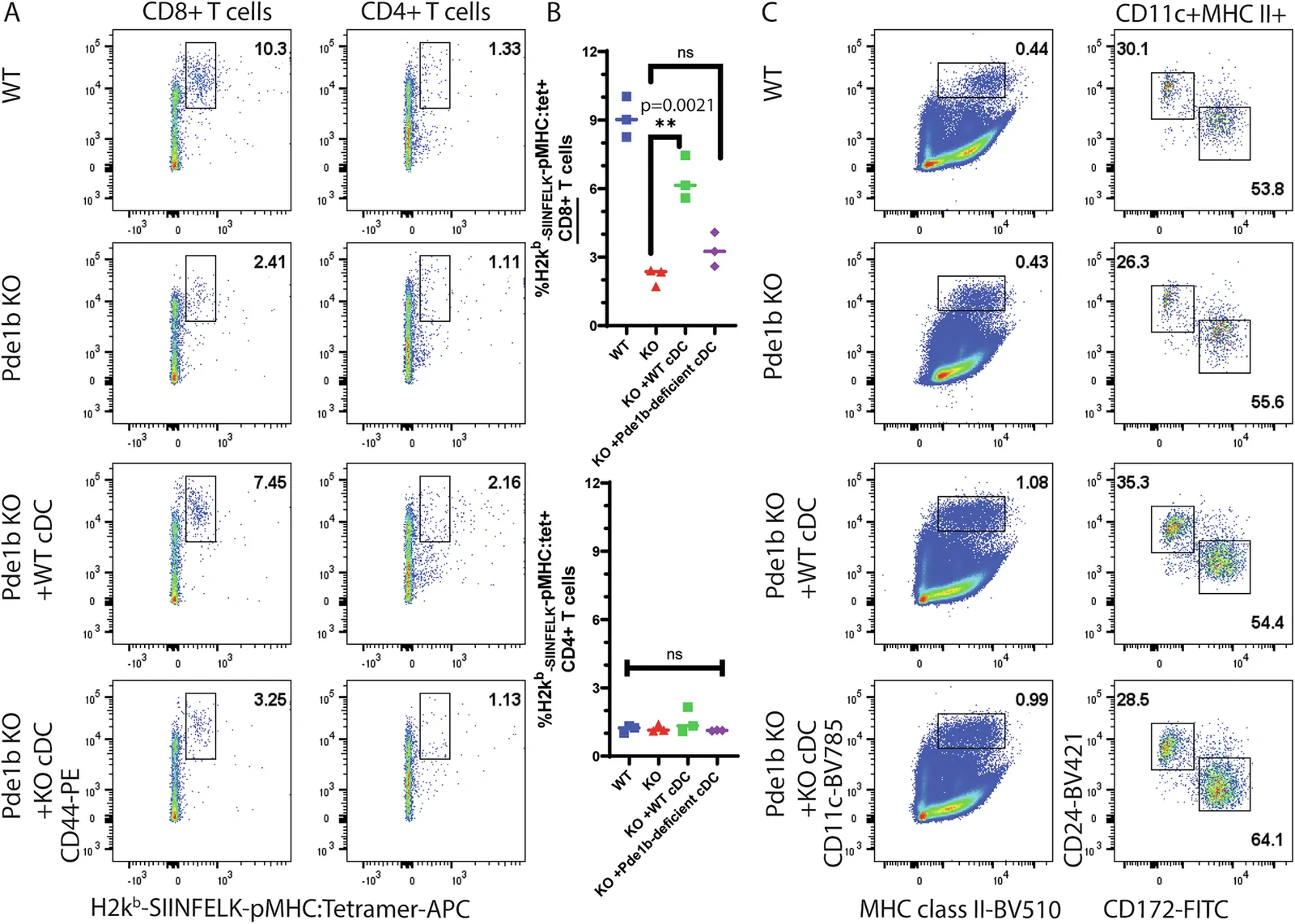
Adoptive transfer of WT dendritic cells partially restores antigen-specific CD8+ T cell priming in Pde1b−/− recipient mice following *Listeria* infection in vivo. (**A**) Flow cytometric plots showing expansion of LM-OVA-specific CD8+ T cell responses in WT, Pde1b−/−, and Pde1b−/− receiving adoptive transfer of WT or Pde1b-deficient splenic cDC prior to LM-OVA infection. (**B**) Quantification of antigen-specific CD8+T cell responses in WT, Pde1b−/−, and adoptive transfer groups. Data shown as mean ± SEM from three biological replicates per condition. CD4+ T cell populations lacking tetramer staining served as internal controls for antigen- and MHC class-specificity. Data shown as mean ± SEM. (**C**) Flow cytometric analysis of splenic CD11c+MHC class II+ cDC following adoptive transfer, confirming successful engraftment of transferred cDC populations in recipient mice. Statistical analyses were performed using two-tailed unpaired Student’s *t*-tests. Data were shown as mean ± SEM. Exact *p* values are indicated in the figures. Statistical significance is denoted as *p < 0.05 and **p < 0.01; ns not significant. Source data are available online for this figure.

To corroborate whether impaired CD8+ T cell priming in Pde1b−/− mice reflects a defect intrinsic to dendritic cells in vivo, splenic-derived WT or Pde1b-deficient cDC were adoptively transferred into Pde1b−/− mice prior to infection with LM-OVA. Transfer of WT cDC partially restored LM-specific CD8+ T cell expansion, whereas transfer of Pde1b-deficient cDC failed to rescue this defect and remained comparable to Pde1b−/− mice without transfer (Fig. 4A,B). As an internal control, MHC class II-restricted CD4+ T cells showed no tetramer staining, confirming antigen- and MHC-specificity. Successful in vivo adoptive cell transfer was validated by measuring the frequency of CD11c+ MHC class II+ cells in the spleen (Fig. 4C).

### Pde1b deficiency induces modest transcriptional remodeling in cDC1 without altering subset development

**Figure 5:**
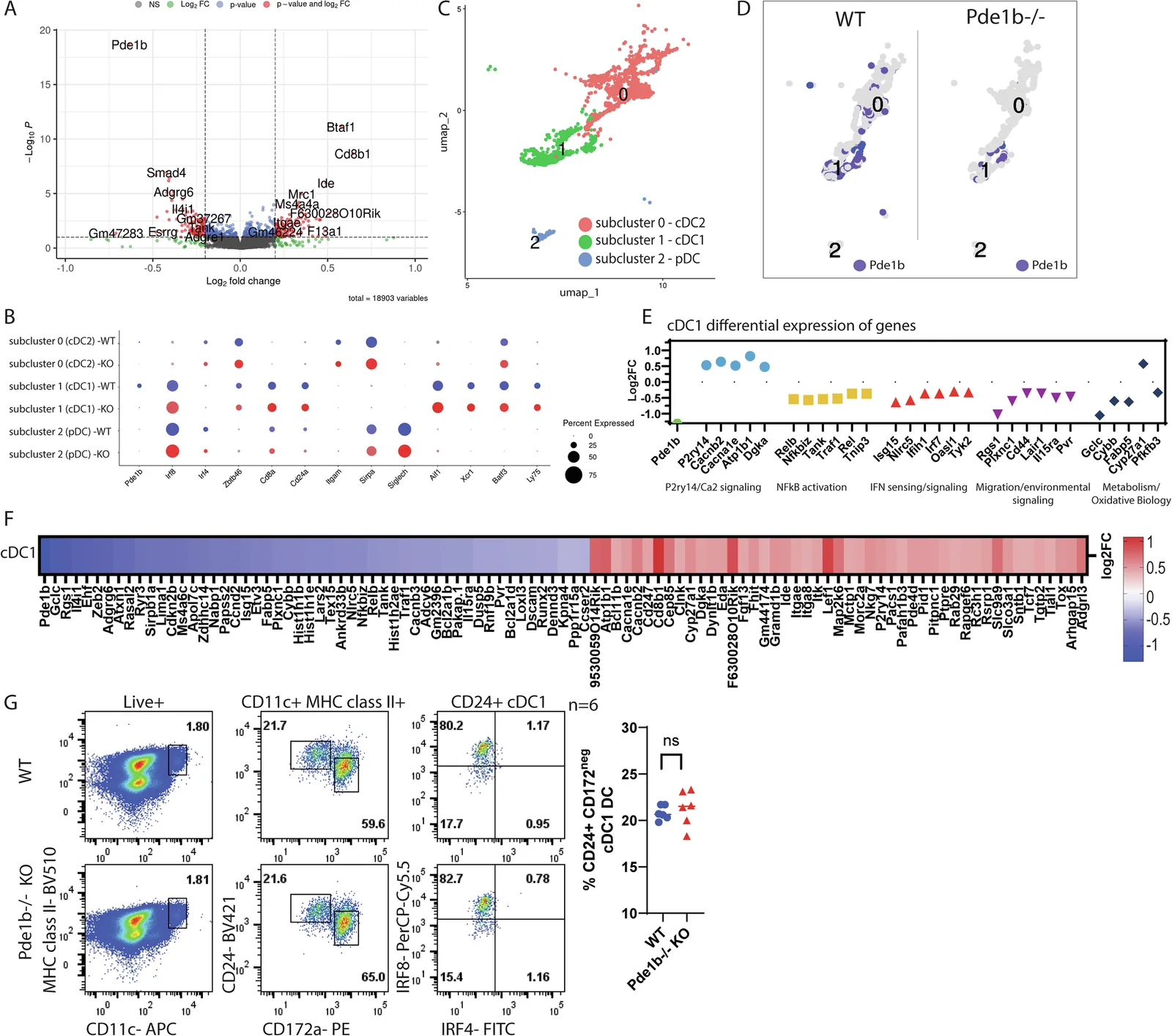
Pde1b deficiency alters transcriptional programs in dendritic cells, but does not impede development of cDC1 or cDC2. (**A**) Volcano plot of differentially expressed genes (DEGs) in DC from WT and Pde1b−/− spleens. Positive log2 fold-change values indicate genes upregulated in Pde1b−/− cells relative to WT, whereas negative values indicate downregulation in Pde1b−/− cells. (**B**) Dot plot showing marker-based identification of DC subsets (cDC1, cDC2, and pDC). (**C**) UMAP clustering showing cDC1, cDC2, and pDC subsets identified from the DC cluster. (**D**) Feature plot showing Pde1b expression across DC subset clusters. (**E**) Curated panel of 30 differentially expressed genes selected from the cDC1 DEG set based on effect size and functional relevance to dendritic cell biology, highlighting calcium signaling, NF-kappa B-mediated inflammatory responses, interferon/antiviral programs, migratory and environmental sensing pathways, and metabolic/oxidative regulation. Log2 fold-change values are shown; values of plus/minus 0.5 and plus/minus 1 correspond approximately to ~1.4-fold and 2-fold changes in expression, respectively. (**F**) Heatmap of expanded top 95 differentially expressed genes in cDC1 cells between WT and Pde1b−/− mice, ranked by log2 fold-change among genes with differential expression. (**G**) Flow cytometric quantification of cDC1 and cDC2 frequencies using MHC class II, CD11c, CD24, and CD172a; IRF8 confirms cDC1 identity. Data in panels (**A**) through (**F**) are derived from scRNA-seq analysis of *n* = 4 mice per genotype. Data in panel (**G**) represent flow cytometric quantification from *n* = 6 mice per group, shown as mean ±  SEM. Differential expression analysis was performed using standard scRNA-seq statistical frameworks as described in Methods. Statistical analyses were performed using two-tailed unpaired Student’s *t*-tests. Exact *p* values are indicated in the figures. Statistical significance is denoted as *p < 0.05 and *p* < 0.01; ns not significant. Source data are available online for this figure.

**Figure EV2:**
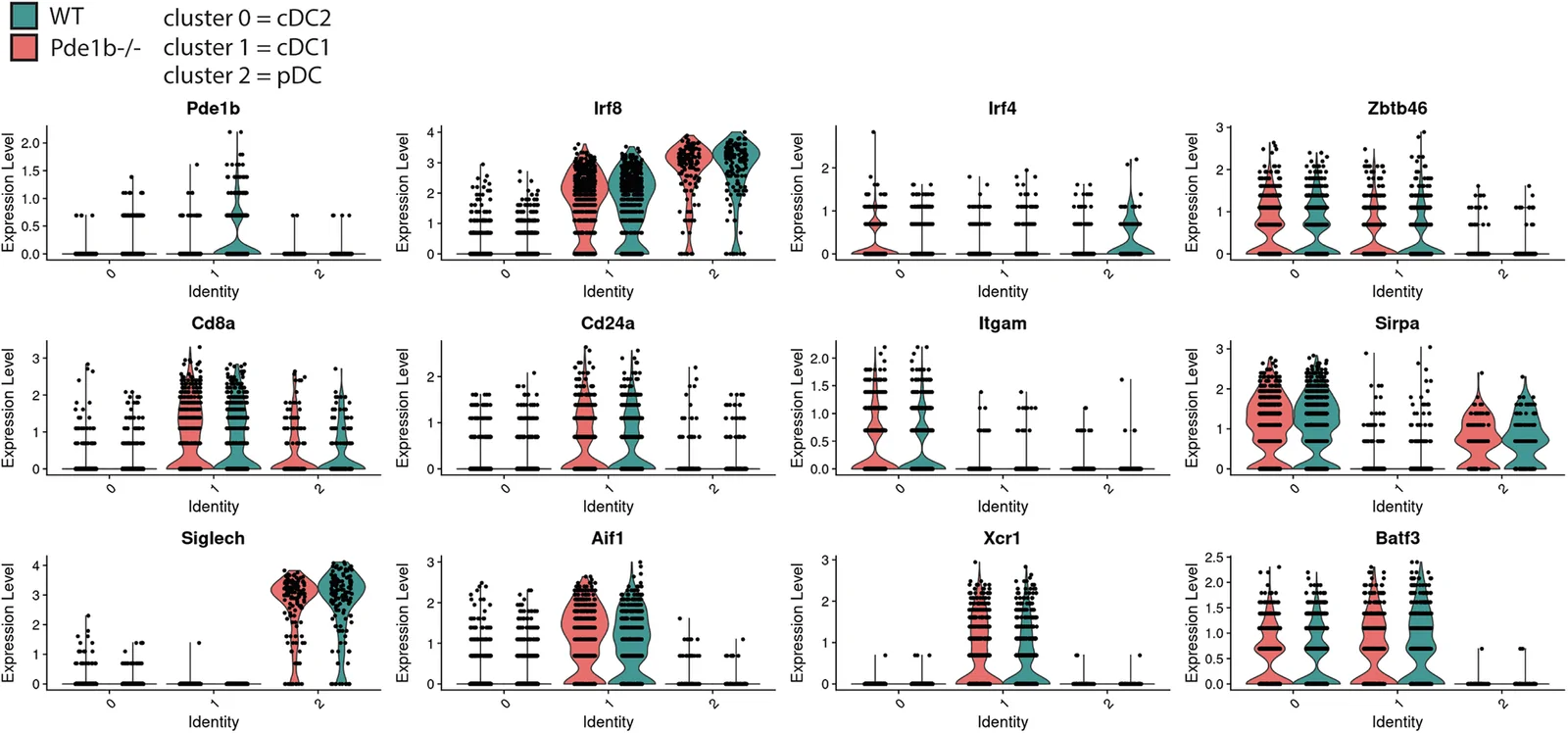
Identification of DC subsets using lineage-specific markers. scRNA-seq analysis (*n* = 4 mice per genotype) was used to probe Irf8, Irf4, Zbtb46, Cd8a, Cd24a, Itgam, Sirpa (CD172a), Siglech, Aif1, Xcr1, and Batf3 in the distinct DC subclusters to determine cDC2, cDC1, and pDC subsets. Cluster 0 = cDC2, cluster 1 = cDC1, and cluster 2 = pDC.

**Figure EV3:**
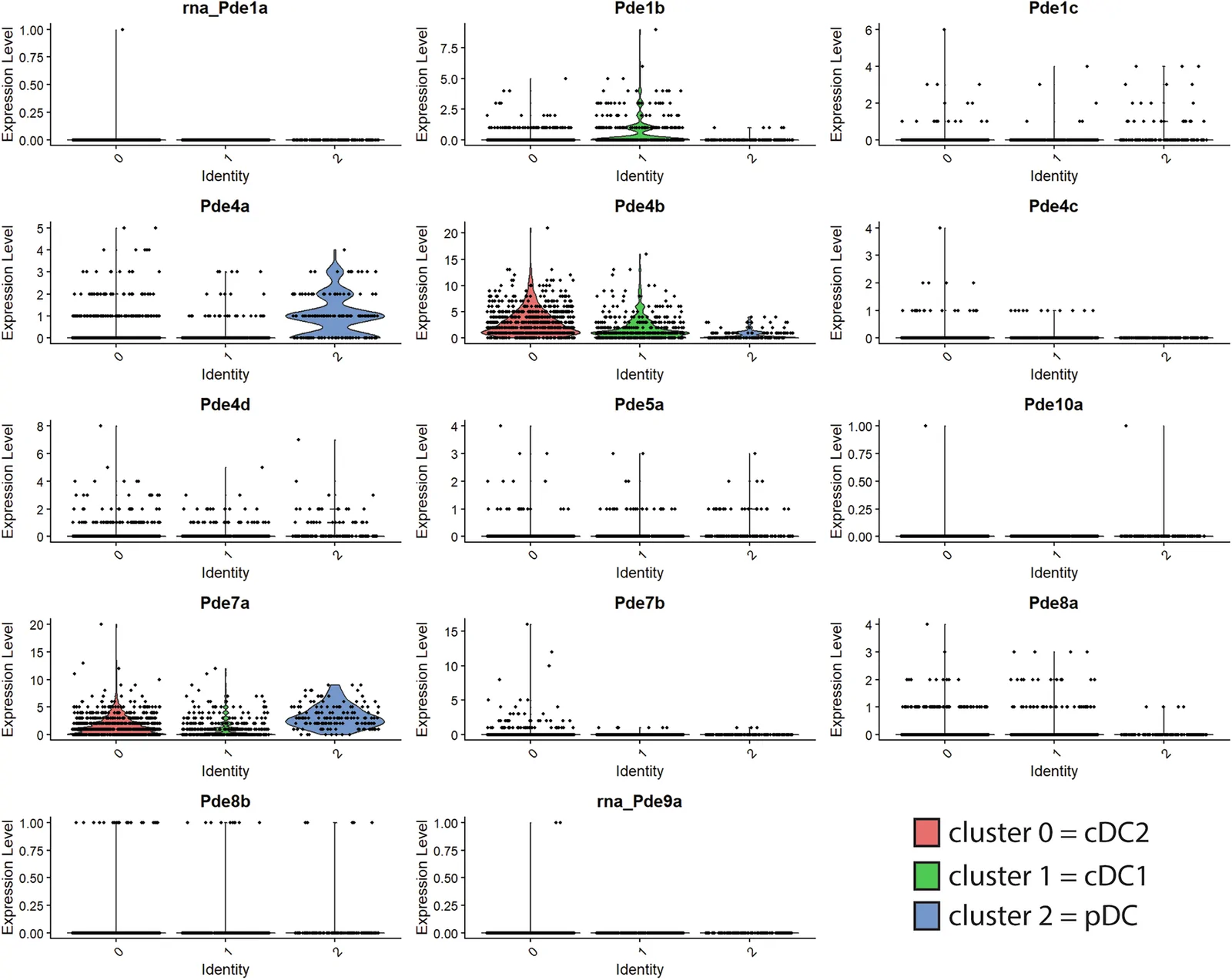
Expression of phosphodiesterase family members in dendritic cell subsets. Single-cell RNA sequencing (scRNA-seq) analysis of DC subset clusters showing expression of Pde1a, Pde1b, Pde1c, Pde4a, Pde4b, Pde4c, Pde4d, Pde5a, Pde7a, Pde7b, Pde8a, Pde8b, Pde9a, and Pde10a (*n* = 4 mice per genotype). Expression profiles are presented as violin plots. Data shown as mean from integrated biological replicates.

To determine how deficiency in Pde1b alters cDC functional capacities, transcriptional profiling and immunophenotyping were performed using scRNA-seq and flow cytometric analyses. Within the DC cluster, a limited number of major transcriptional changes were observed, reflecting modest global alterations (Fig. 5A). These included increased expression of Batf1, Ide, Cd8b1, and Mrc1, alongside reduced expression of Pde1b, Smad5, Adgrg6, Il4i1, and Tank in Pde1b-deficient mice. Deeper analysis of the DC compartment enabled identification of distinct subsets through integration of UMAP-based cluster segregation and canonical lineage marker expression, identifying classical DCs (cDCs, spanning both cDC1 and cDC2) via Batf3 and Zbtb46 expression to distinguish them from plasmacytoid DCs (pDCs), with cDC1 further defined by Xcr1, Irf8, Aif1, Cd8a, and Cd24; cDC2 defined by Itgam (CD11b) and Sirpa (CD172a); and pDCs defined by Siglech and Irf8 (Figs. 5B,C and EV2). Pde1b expression was largely restricted to the cDC1 cluster (Fig. 5D). Notably, both Pde4b and Pde7a phosphodiesterase genes were also present within cDC1 (Fig. EV3); no other PDE family members or isoforms were detected.

Differential gene expression analysis was performed in cDC1 cells comparing WT and Pde1b^−^/^−^ mice. At steady state, loss of Pde1b resulted in modest but coordinated transcriptional changes. Functional annotation of differentially expressed genes identified alterations in transcripts associated with inflammatory, cytokine-responsive, interferon-related, migratory and metabolic programs (Fig. 5E). Among these changes, expression of NFκB-associated genes, including Nfkbiz, Relb, Traf1, and Rel was reduced, while genes linked to purinergic and cyclic nucleotide signaling, including P2ry14, Dgka, and Grk3, were increased. Additional decreases were observed in cytokine-responsive and interferon-associated genes, including Il15ra, Tyk2, Jak2, Il21r, Irf7, Nlrc5, and Isg15. Examination of the broader set of top differentially expressed genes revealed that these changes were not restricted to a select number, but instead reflect a distributed transcriptional signature characterized by numerous modest shifts in cDC1 gene expression profile in absence of Pde1b at steady state (Fig. 5F). Importantly, flow cytometric analysis showed no significant differences in the frequencies of cDC1 or cDC2 populations (Fig. 5G), indicating that Pde1b is dispensable for DC subset development and homeostasis at steady state.

### Pde1b marks splenic macrophage heterogeneity and is associated with distinct transcriptional programs

**Figure 6:**
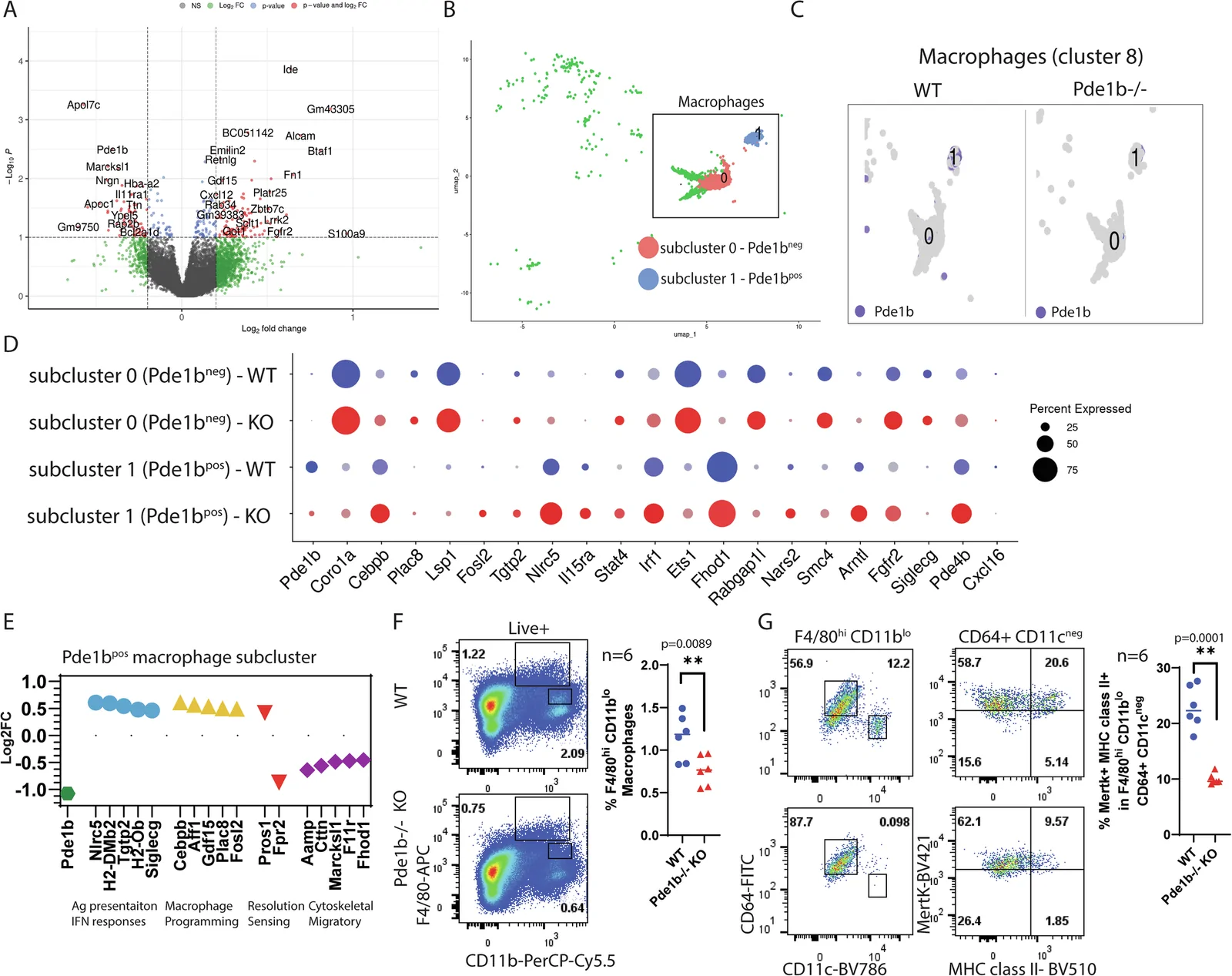
Pde1b deficiency promotes an immunosuppressive phenotype in macrophages under steady-state conditions. (**A**) Volcano plot of differentially expressed genes (DEGs) in the macrophage subcluster. Positive log2 fold-change values indicate genes upregulated in Pde1b−/− cells relative to WT, whereas negative values indicate downregulation in Pde1b−/− cells. (**B**,** C**) Identification of macrophage subclusters based on transcriptional profiles; a non-macrophage cluster enriched for immunoglobulin genes was excluded. (**D**) Dot plot showing differential gene expression across macrophage subclusters. (**E**) Curated panel of DEG selected from the Pde1b^pos^ macrophage subcluster based on effect size and functional relevance, highlighting antigen presentation and interferon-responsive programs, macrophage state regulation, environmental sensing pathways and cytoskeletal/migratory remodeling. Log2 fold-change values are shown; values of ±0.5 and ±1 correspond approximately to ~1.4-fold and 2-fold changes in expression, respectively. (**F**) Flow cytometric quantification of F4/80+CD11b^lo^ macrophages. Data shown as a mean ± SEM from 6 biological replicates. (**G**) Phenotypic characterization of the macrophage subsets based on MertK and MHC class II expression within the F4/80+CD11b^lo^CD64+CD11c^neg^ macrophage population. scRNA-seq data were generated from *n* = 4 mice per genotype. Flow cytometry data were shown as mean ± SEM with *n* = 6 mice per group. Differential expression analysis was performed using standard scRNA-seq statistical frameworks as described in Methods. Statistical analyses were performed using two-tailed unpaired Student’s *t*-tests. Data were shown as mean ± SEM. Exact *p* values are indicated in the figures. Statistical significance is denoted as *p < 0.05 and **p < 0.01; ns, not significant. Source data are available online for this figure.

**Figure EV4:**
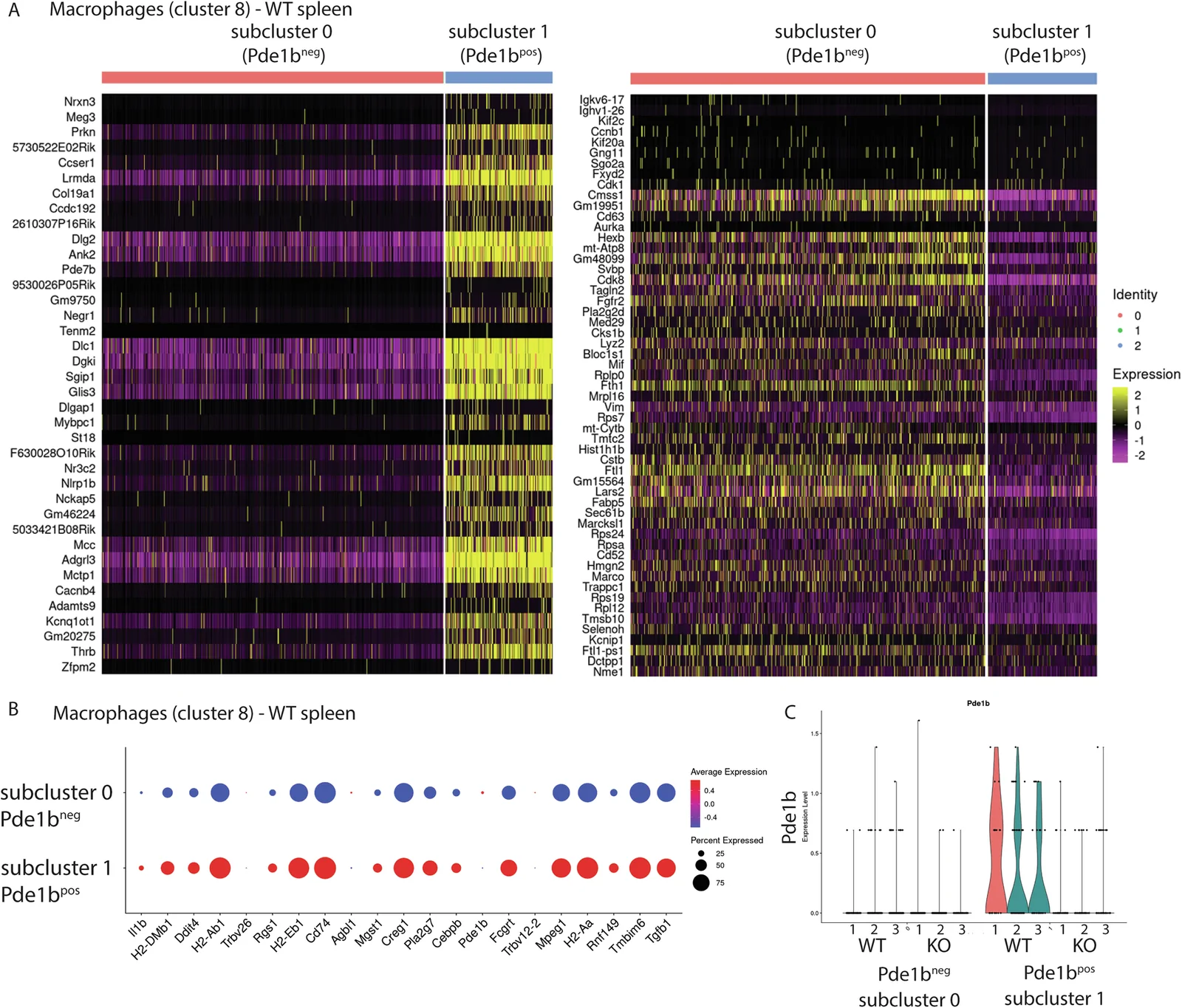
Macrophage (cluster 8) differential gene expression. (**A**) Heatmap table from single-cell RNA sequencing (scRNA-seq) showing differentially expressed genes (DEGs) between subcluster 0 (Pde1b-negative) and subcluster 1 (Pde1b-positive) within the parent macrophage cluster 8 of WT samples (*n* = 4 mice per genotype). (**B**) Dot plot illustrating frequency and expression levels of top DEG in subcluster 0 and subcluster 1 of WT splenic macrophages (*n* = 4 mice per genotype). (**C**) Analysis of biological replicates for uniformity in identification and expression of Pde1b in the unique Pde1b-positive subcluster, but not the Pde1b-negative cohort (*n* = 4 mice per genotype).

Comparative transcriptional profiling of splenic macrophages revealed increased expression of Fn1, Ide, Alcam, Lrk2, Cxcl12, and Fgfr2 in Pde1b-deficient macrophages, concomitant with decreased expression of Apoc1, Apol7c, Marcksl1, and Il11ra1 (Fig. 6A). Deeper analyses identified two subclusters in macrophages, with Pde1b expression restricted to subcluster 1 (Pde1b^pos^) (Fig. 6B,C). Several genes were differentially expressed between Pde1b^pos^ and Pde1b^neg^ in WT spleen, indicating transcriptional heterogeneity (Fig. EV4A,B). Differential expression analysis within Pde1b^pos^ macrophages identified genes altered between WT and Pde1b−/− cells, which were subsequently examined across macrophage subclusters 0 and 1. Analysis of biological replicates confirmed reproducible identification of the Pde1b^pos^ macrophage subset, with Pde1b expression consistently detected in the Pde1b^pos^ cluster but absent from the Pde1b^neg^ population (Fig. EV4C). Notably, the Pde1b^pos^ subcluster remained identifiable in Pde1b−/− mice, indicating that Pde1b expression itself was not required for the establishment of this macrophage subpopulation. Examination of WT and Pde1b−/− macrophages revealed increased expression of Cebpb, Folr2, Stat4, Arntl, Fgfr2, and Nars2 within the Pde1b^pos^ subset of knockout mice (Fig. 6D), consistent with a potential shift in inflammatory programming. Curated differential expression analysis of the Pde1b^pos^ macrophage subcluster revealed modest transcriptional changes, including increased expression of antigen presentation and regulatory genes (Nlrc5, H2-DMb2, Cebpb, and Fosl2) and selective reduction of the environmental sensing receptor Fpr2, a G-protein-coupled receptor involved in chemotactic responses, tissue-derived danger sensing and pro-resolving inflammatory signaling (Fig. 6E). Subset-specific analyses by flow cytometry showed reduced frequency of F4/80+CD11b^lo^ splenic macrophages (Fig. 6F). Within the F4/80+CD11b^lo^CD64+CD11c^neg^ compartment, Pde1b−/− macrophages exhibited reduced MHC class II expression (Fig. 6G). Collectively, Pde1b deficiency reduces the frequency of a defined splenic macrophage subset at steady state and is associated with modest but coordinated remodeling of immune sensing and antigen presentation programs within this compartment.

### Pde1b loss elevates cyclic nucleotide and kinase activities

**Figure 7:**
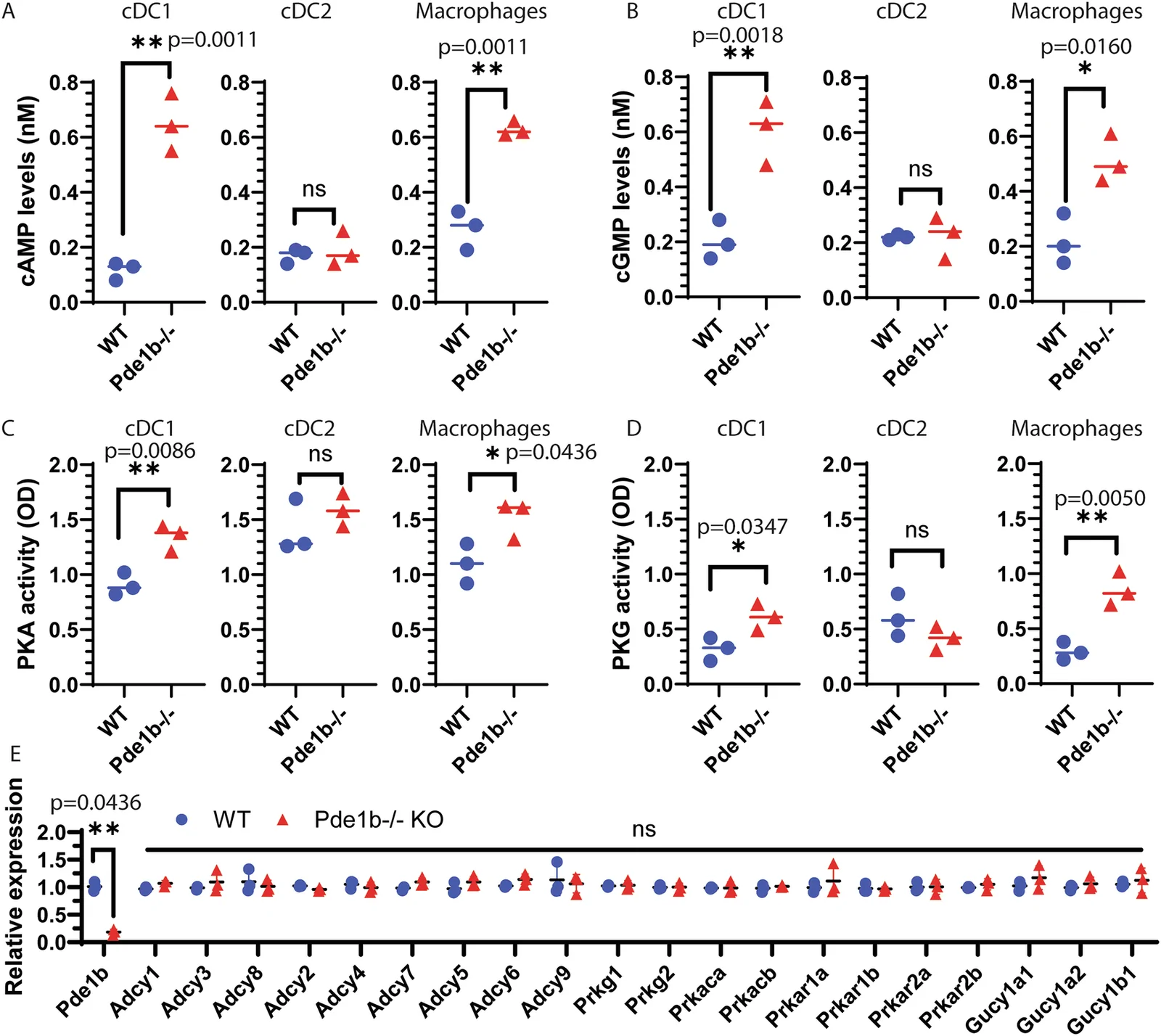
Pde1b regulates cyclic nucleotide signaling and kinase activation in myeloid cells. (**A**,** B**) Intracellular cAMP and cGMP levels measured in FACS-sorted cDC1, cDC2, and macrophages from WT and Pde1b^−^/^−^ mice. (**C**,** D**) PKA and PKG kinase activity were measured in the same populations. (**E**) scRNA-seq analysis of cyclic nucleotide signaling components in cDC1. Data were shown as mean ± SEM from *n* = 4 biological replicates. Statistical analyses were performed using two-tailed unpaired Student’s *t*-tests. Exact *p* values are indicated in the figures. Statistical significance is denoted as *p < 0.05 and *p* < 0.01; ns not significant. Source data are available online for this figure.

Studies next evaluated the downstream changes in cAMP-PKA and cGMP-PKG in cDC and macrophages in the absence of Pde1b. Under steady-state, splenic-derived FACS-purified cDC1 and macrophages from Pde1b-deficient mice exhibited significantly elevated intracellular levels of both cAMP and cGMP compared to WT controls (Fig. 7A,B). By contrast, cDC2 subsets showed no change in cyclic nucleotide concentration, demonstrating a selective role for Pde1b in specific myeloid APC lineages. Correspondingly, loss of Pde1b resulted in increased activities of both PKA and PKG kinase in cDC1 and macrophages, but not in cDC2 populations, as quantified by kinase phosphorylation assays (Fig. 7C,D). Comparison of WT and Pde1b−/− cDC1 and macrophages revealed no significant changes in expression of adenylate cyclase, soluble guanylate cyclase or PKA and PKG subunits, indicating that elevated signaling is not accompanied by compensatory transcriptional remodeling of upstream or downstream pathway components (Fig. 7E). Taken together, these data reveal that Pde1b limits cyclic nucleotide signaling and downstream kinase activation.

### Acute inhibition of PDE1, but not PDE4 or PDE7, phenocopies the impaired immune priming observed in Pde1b-deficient cells in vitro

**Figure 8:**
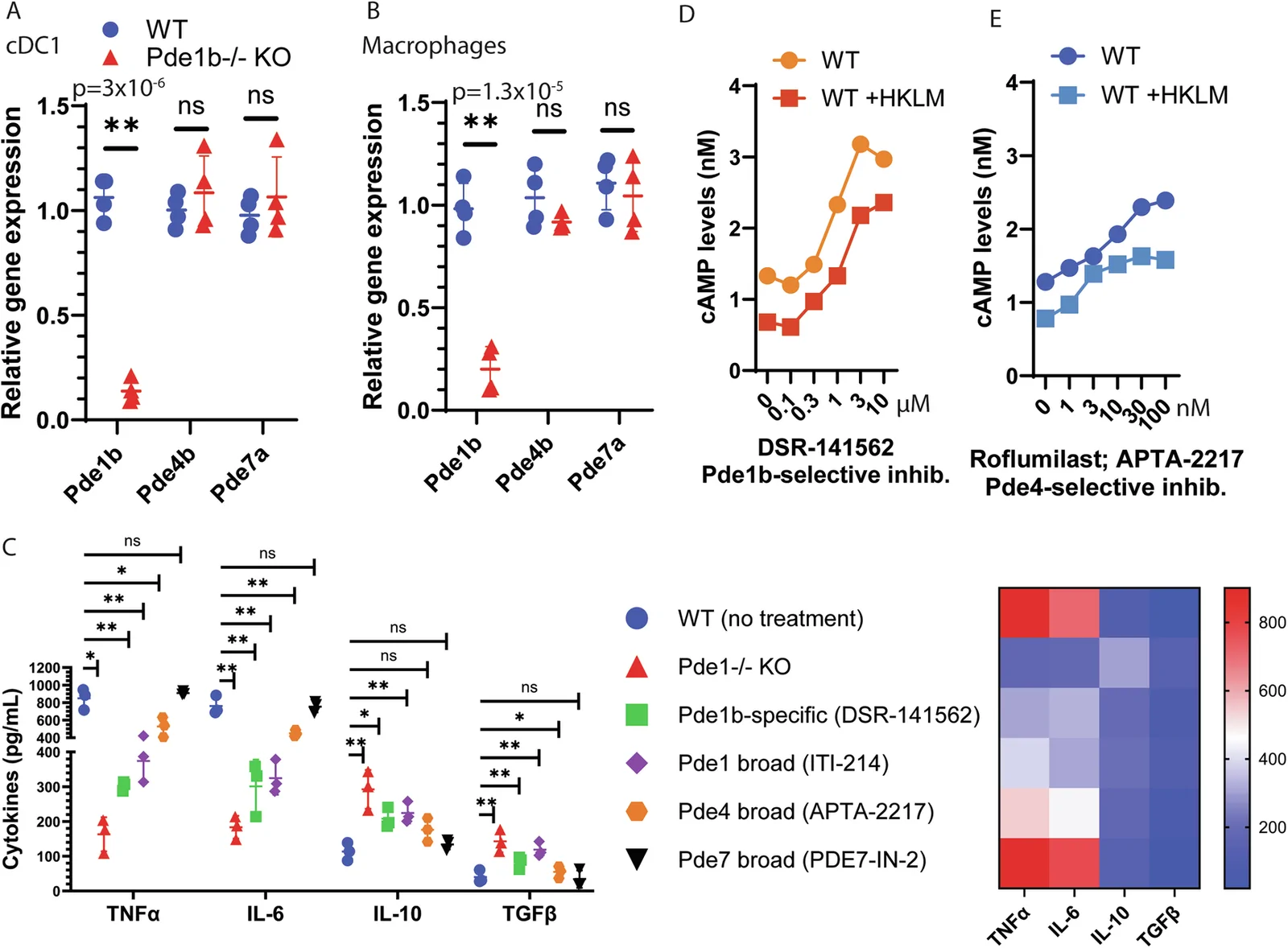
Pharmacological inhibition of PDE1 phenocopies loss of Pde1b and reveals isoform-specific control of inflammatory signaling. (**A**, **B**) Gene expression analysis of PDE isoforms (Pde1b, Pde4b, and Pde7a) in cDC1 and macrophages derived from scRNA-seq data (*n* = 4 mice per genotype). Expression values were normalized to WT controls (set to 1), and relative expression in Pde1b−/− cells is shown. (**C**) Cytokine expression in splenic-derived DC following TLR2 stimulation (HKLM) in the presence of selective inhibitors targeting Pde1b (DSR-141562; Pde1b-preferential), pan-PDE1 (ITI-214), PDE4 (roflumilast), and PDE7 (PDE7-IN-2). Data shown as mean ± SEM from *n* = 3 biological replicates. Exact *p* values for WT compared to Pde1−/− KO, Pde1b-preferential inhibitor, Pde1 broad inhibitor, PDE4 broad inhibitor, and PDE7 inhibitor are as follows: TNFα: 0.00075, 0.00136, 0.00324, 0.02716, and ns, respectively; IL-6: 0.00083, 0.00367, 0.00288, 0.00864, and ns, respectively; IL-10: 0.00727, 0.00967, 0.00815, ns, and ns, respectively; and TGFβ: 0.00809, 0.04309, 0.00806, ns, and ns, respectively. (**D**, **E**) Intracellular cAMP levels in cDC following graded pharmacological inhibition of PDE1 or PDE4 under basal and HKLM-stimulated conditions. Data are shown as mean ± SEM from *n* = 3 biological replicates; each replicate represents a culture derived from separate mice. Statistical analyses were performed using two-tailed unpaired Student’s *t*-tests. Exact *p* values are indicated in the figures. *p < 0.05, *p* < 0.01, and ns not significant. Source data are available online for this figure.

**Figure EV5:**
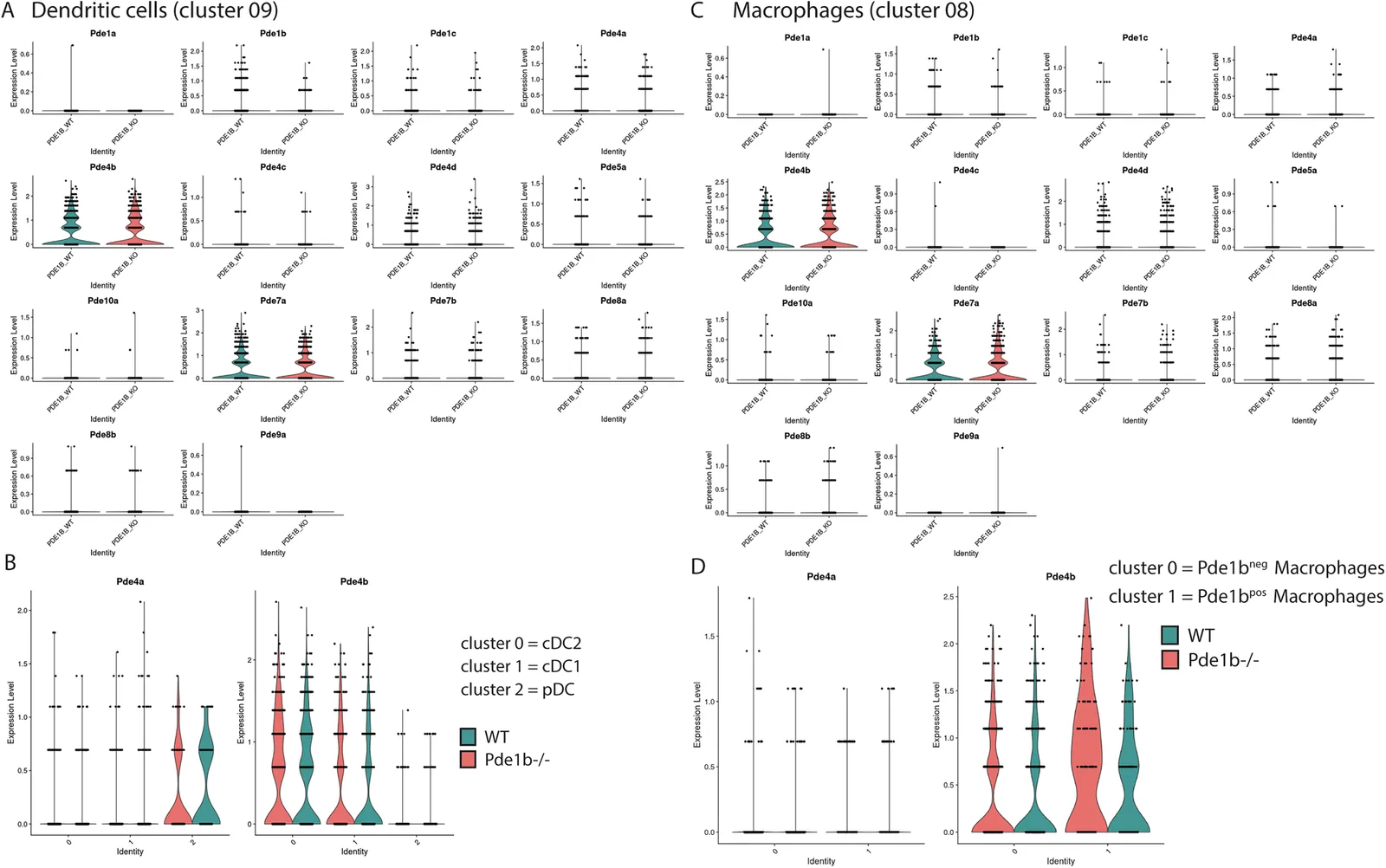
Lack of compensatory upregulation of PDE isoforms in Pde1b-deficient mice. Expression levels of PDE isoforms in (**A**) total DC cluster 9 and of (**B**) Pde4a and Pde4b within distinct DC subclusters analyzed from scRNA-seq data in WT and Pde1b−/− mice (*n* = 4 mice per genotype). Expression levels of PDE isoforms in (**C**) total macrophage cluster 8 and of (**D**) Pde4a and Pde4b within distinct macrophage subclusters analyzed from scRNA-seq data in WT and Pde1b−/− mice (*n* = 4 mice per genotype). Data shown as mean from integrated biological replicates.

To determine whether this phenotype reflects specificity for PDE1 or redundancy with other phosphodiesterases, we first assessed expression of candidate PDE family members. Pde4b and Pde7a genes were expressed in both cDC1 and macrophages, with no evidence of compensatory upregulation in either population or distinct subsets in Pde1b−/− mice (Figs. 8A,B and EV5A,B). We next evaluated the functional contribution of these enzymes using pharmacological inhibitors in primary DC. These included a PDE1-selective inhibitor (DSR-141562; preferential for PDE1B, IC50 = 43.9 nM vs PDE1A = 97.6 nM and PDE1C = 431.8 nM), a pan-PDE1 inhibitor (ITI-214), a PDE4 inhibitor (roflumilast; APTA-2217) and a PDE7 inhibitor (PDE7-IN-2). Concentrations were selected based on published IC50 values where available. As these compounds differ in isoform selectivity, results were interpreted as pathway-level inhibition. PDE1 inhibition markedly reduced inflammatory cytokine production following TLR2 stimulation with heat-killed *Listeria monocytogenes* (HKLM), whereas PDE4 inhibition produced only partial effects, and PDE7 inhibition had no detectable impact (Fig. 8C). To relate these functional effects to cyclic nucleotide signaling, intracellular cAMP was measured under basal and HKLM-stimulated conditions across increasing inhibitor concentrations. PDE1 inhibition produced a larger increase in cAMP than PDE4 inhibition across all doses tested, although both inhibitors elevated cAMP in a dose-dependent manner (Fig. 8D,E).

### Absence of Pde1b results in increased phosphorylation of CREB and GSK3 in DC and macrophages

**Figure 9:**
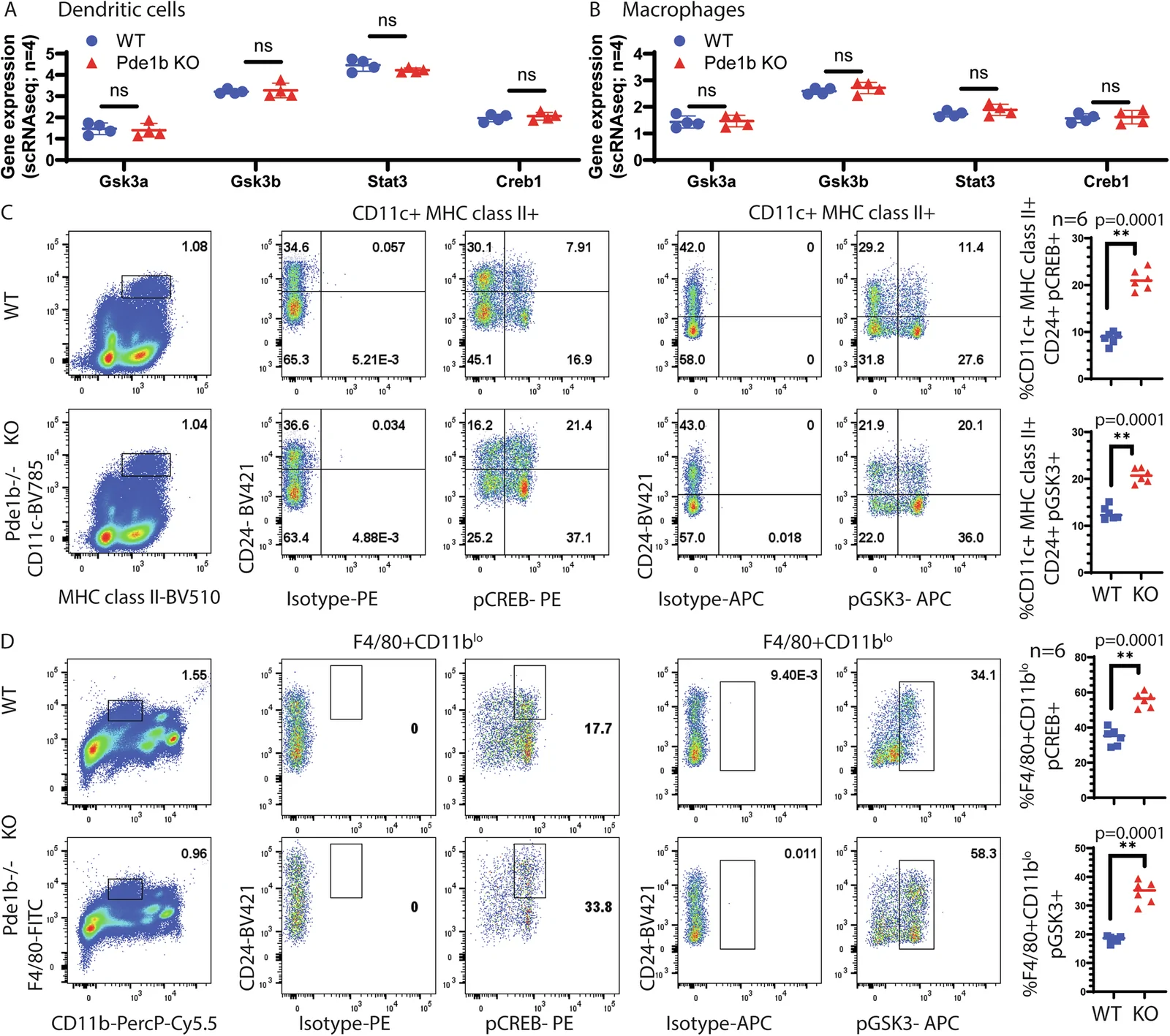
Increased phosphorylation of GSK3 and CREB in Pde1b-/- DC and macrophages. (**A**,** B**) sc-RNAseq of Gsk3a, Gsk3b, Stat3, and Creb1 in splenic-derived cDC and macrophages. (**C**) Phospho-flow cytometric analysis of CD11c+ MHC class II + DC for phosphorylated-CREB_S133_ and -GSK3β_S9_; CD24 used to denote the cDC1 population. All gates established using isotype controls. Bar graphs display the mean and standard deviation of 9 replicates. (**D**) Phospho-flow cytometric analysis of F4/80+CD11b^lo^ macrophages for phosphorylated-CREB_S133_ and -GSK3β_S9_. Bar graphs display 9 replicates. Statistical analyses were performed using two-tailed unpaired Student’s *t*-tests. Data were shown as mean ± SEM. Exact *p* values are indicated in the figures. Statistical significance is denoted as *p < 0.05 and **p < 0.01; ns not significant. Source data are available online for this figure.

To determine downstream consequences of heightened cyclic nucleotide-driven protein kinase activity, phosphorylation states of CREB and GSK3 were assessed in sorted DC and macrophage populations. Single-cell transcriptomics showed no significant changes in levels of total Gsk3a, Gsk3b, Stat3 or Creb1 expression in either DC or macrophages (Fig. 9A,B). Phospho-flow cytometric analysis of DC subsets revealed increased levels of phosphorylated CREB at Serine 133 (pCREB_S133_) and GSK3β at serine 9 (pGSK3β_S9_) in the cDC1 subsets (Fig. 9C). Parallel analysis of the F4/80+CD11b^lo^ macrophages also observed increased levels of pCREB_S133_ and moderately elevated levels of pGSK3β_S9_ (Fig. 9D). These post-translational modifications correlate with suppressed inflammatory gene signatures and are consistent with reprogramming toward immunoregulatory transcriptional states, providing a mechanistic basis for reduced inflammatory and antigen presentation capacity.

## Discussion

Cyclic nucleotide phosphodiesterases are increasingly recognized as important regulators of immune signaling by imposing constraints on intracellular second messenger dynamics. PDE1 family members have emerged as potential lineage-restricted regulators of immune cell state. Pde1b is selectively enriched in cDC1 and subsets of macrophages, as well as select granulocyte populations, but is absent from lymphoid lineages. In turn, recent work shows that Pde1a and Pde1b regulate cAMP-PKA signaling in T cells to support stem-like, anti-exhaustion states (Li et al, 2026). Together, these findings support a model in which cyclic nucleotide signaling is a core regulatory system governing immune cell function, with PDE1 isoforms acting as modulators of signaling output across immune compartments.

Genetic deletion of Pde1b impaired clearance of intracellular bacterial infection and inhibited antigen-specific CD4+ and CD8+ T cell responses, accompanied by a cytokine profile consistent with enhanced anti-inflammatory and T_H_17-associated programs and reduced type 1-associated responses. These collective changes indicate a failure to sustain cell-mediated immunity, with a concomitant shift toward regulatory and alternative inflammatory states. This phenotype indicates that Pde1b functions upstream in myeloid antigen-presenting cell programming that is required to appropriately instruct type 1-polarized adaptive immune responses. Its loss is associated with a breakdown in DC and macrophage capacity to support the generation and maintenance of type 1 effector responses during infection.

Prior studies have established that cAMP signaling within DC is a key determinant of T cell polarization and immune outcome, where elevated cAMP biases responses priming away from type 1 immunity and can redirect helper T cell differentiation in a context-dependent manner (Li et al, 2012; Lee et al, 2015; Datta et al, 2010), and similar cAMP-dependent mechanisms in macrophages have been shown to suppress pro-inflammatory (M1-like) activation programs while favoring regulatory states (Rayees et al, 2019). In this context, the phenotypes observed following Pde1b deletion are consistent with dysregulated cyclic nucleotide signaling within antigen-presenting cells, supporting the immunoregulatory role of Pde1b in modulating immunity vs. tolerance.

Pde1b-deficient cDC1 have reduced IL-12 production and impaired antigen-specific CD8 + T cell priming in vivo, which is partially rescued by adoptive transfer of WT DC. This partial rescue is consistent with the broader cellular context of Pde1b deficiency, in which multiple myeloid compartments, such as macrophages and granulocytes, also lack Pde1b, such that restoration of DC function alone reconstitutes a portion of the impaired immune response. As a potential limitation, recipient mice harbor endogenous Pde1b-deficient cDC1 alongside transferred wild-type cDC1. This cellular co-existence introduces the possibility of bidirectional crosstalk influencing effector states or direct competition for antigen and cognate T cells during priming. Although the results show partial restoration, in the absence of a cDC1-deficient host model, we cannot conclusively rule out that endogenous mutant cDC1 actively impede or outcompete adoptively transferred cells. Furthermore, because macrophage populations remain Pde1b-deficient, these experiments cannot definitively establish whether cDC1 restoration alone is fully sufficient for maximal effector T cell responses without concurrent correction across cooperating myeloid compartments. To assess whether these functional defects in DC reflected altered activation preparedness, transcriptomic profiling of cDC1 at steady state was performed to define changes in baseline programs that may constrain subsequent effector responses. This analysis revealed coordinated reduction of Irf7- and Relb-associated transcriptional programs, diminished ISG15 expression and increased P2ry14, which suggests a shift away from interferon-responsive and NFκB–linked maturation states towards a program more consistent with altered sensing of inflammatory and stress-associated cues. This coordinated remodeling suggests that the phosphodiesterase is required to maintain a cDC1 state that is transcriptionally poised for type 1 immune priming.

A complementary immune rewiring is observed in macrophages following Pde1b deletion, accompanied by reduced macrophage abundance and decreased MHC class II expression by flow cytometry, indicating diminished antigen-presenting capacity. Increased expression of Cebpb is associated with macrophage transcriptional programs linked to tissue-repair–associated activation states (Ruffell et al, 2009). Decreased expression of Fpr2 is notable, as this receptor has been implicated in macrophage efferocytosis and inflammation-resolution pathways, where reduced signaling is associated with impaired resolution of inflammatory responses (Wu et al, 2023). Increased expression of Siglecg is consistent with elevated inhibitory receptor tone, where the gene modulates activation thresholds in TLR-, antiviral and phagocytic programs (Chen et al, 2013; Qin et al, 2026). Increased expression of Fn1 is notable, as FN1+ macrophage states have been shown to exhibit high efferocytosis activity and tissue-remodeling programs associated with pro-tumoral immunosuppressive remodeling and reduced antitumor immune effectiveness (Xi et al, 2026). Collectively, these changes indicate a coordinated shift away from effective antigen-presenting and inflammatory competence toward reduced myeloid activation capacity during antibacterial immune responses.

To better define the enzymatic architecture governing cyclic nucleotide signaling in these cells, deep scRNA-seq profiling of both cDC1 and macrophages revealed selective expression of PDE1, PDE4, and PDE7 family members. PDE4 has been widely implicated in maintaining basal cAMP homeostasis and establishing inflammatory set-points in myeloid antigen-presenting cells, and is regulated in part by MAPK-ERK signaling pathways that contribute to steady-state control of cyclic nucleotide signaling (Jin et al, 2005; Pryzwansky et al, 1998; Shepherd et al, 2004). PDE7 is thought to contribute more selectively to context-dependent tuning of activation thresholds. Despite these distinct roles, neither isoform exhibited compensatory transcriptional adaptation following Pde1b deletion. Pharmacological inhibition of PDE7 had no detectable effect, while PDE4 inhibition produced only modest increases in cAMP, consistent with its role in basal cyclic nucleotide buffering rather than stimulus-coupled signaling control. This is consistent with prior work implicating PDE4 in inflammatory tuning in DC and macrophages, but indicates that PDE4- and PDE7-mediated regulation alone is insufficient to reproduce the activation-coupled cyclic nucleotide control observed in this system.

In contrast to PDE4- and PDE7-mediated basal or context-restricted regulation, Pde1b is uniquely distinguished by its Ca²⁺/calmodulin dependence, which couples receptor-induced calcium flux to localized cyclic nucleotide degradation. This enables Pde1b to be selectively engaged during immune responses, providing dynamic control of cyclic nucleotide signaling in cDC1 and macrophage subsets through spatially restricted cAMP/cGMP hydrolysis, a mode of regulation not achieved by PDE4 or PDE7, to govern pro-inflammatory responses and cell-mediated immunity.

## Methods

**Table:** Reagents and tools table

Reagent/resource	Reference or source	Identifier or catalog number
**Experimental models**		
Pde1b knockout mouse (global KO)	In-house/institutional colony	Not applicable
Wild-type C57BL/6 mice	Jackson Laboratory	JAX #000664
**Recombinant DNA**		
N/A		
**Antibodies**		
CD11c (N418)	BioLegend	117305
MHC class II (M5/114/15.2)	BioLegend	107645
CD24 (M1/69, 30-F1)	BioLegend	101825
CD172a (P84)	BioLegend	144011
IRF8 (V3GYWCH)	Thermo Fisher Scientific	46-9852-82
IRF4 (3E4)	Thermo Fisher Scientific	11-9858-82
B220 (RA3-6B2)	BioLegend	103231
CD3 (17A2)	BioLegend	100203
CD64 (X54-5/7.1)	BioLegend	139301
F4/80 (BM8)	BioLegend	123116
CD11b (M70)	BioLegend	101201
Mertk (2b10c42)	BioLegend	151503
Pde1b (EPR12293(2))	Abcam	ab182565
H2-Kb | chicken ova 257-264 | SIINFEKL	NIH Tetramer Core Facility	134271
2W-I-Ab tetramer	NIH Tetramer Core Facility	132585
CD44 (IM7)	BioLegend	103043
CD8 (53-6.7)	BioLegend	100711
CD4 (RM4-5)	BioLegend	100531
phospho-CREB1 (Ser133) (A22026B)	BioLegend	651402
phospho-GSK3β (1C9E2)	Thermo Fisher Scientific	67558-1-IG
**Oligonucleotides and other sequence-based reagents**		
Pde1b	Thermo Fisher Scientific	Mm00476643_m1
Listeria monocytogenes	Thermo Fisher Scientific	Ba07921936_s1; Ba07922252_s1
Nos2	Thermo Fisher Scientific	Mm00440502_m1
Arg1	Thermo Fisher Scientific	Mm00475988_m1
Il6	Thermo Fisher Scientific	Mm00446190_m1
Tnfa	Thermo Fisher Scientific	Mm00443258_m1
Il12a	Thermo Fisher Scientific	Mm00434169_m1
Il10	Thermo Fisher Scientific	Mm01288386_m1
Tgfb1	Thermo Fisher Scientific	Mm01178820_m1
Il17a	Thermo Fisher Scientific	Mm00439618_m1
Ccr9	Thermo Fisher Scientific	Mm02528165_s1
Cd74	Thermo Fisher Scientific	Mm00658576_m1
Creb1	Thermo Fisher Scientific	Mm00501607_m1
GAPDH (housekeeping)	Thermo Fisher Scientific	Mm99999915_g1
ACTB (housekeeping)	Thermo Fisher Scientific	Mm02619580_g1
**Chemicals, enzymes and other reagents**		
Trizol RNA extraction reagent	Thermo Fisher Scientific	15596018
TaqMan Gene Expression Master Mix	Thermo Fisher Scientific	4444556
CellTrace™ CFSE (488 nm laser) Cell Proliferation Kit, for flow cytometry (180 tests)	Thermo Fisher Scientific	C34554
Luminex multiplex cytokine kit	Thermo Fisher Scientific	custom kit
**Software**		
Seurat (scRNA-seq analysis)	Satija Lab	v4/v5
GraphPad Prism	GraphPad Software	v9+
FlowJo	BD Biosciences	v10+
QuantStudio Real-Time PCR Software	Thermo Fisher Scientific	v1.3+
**Other**		

### Animals

Mouse models used in the study included C57BL/6 (B6; WT), Pde1b−/−(B6.129P2-Pde1btm1Dgen/Mmnc), OT-I (C57BL/6-Tg(TcraTcrb)1100Mjb/J), and OT-II (B6.Cg-Tg(TcraTcrb)425Cbn/J). B6, OT-I and OT-II animals were purchased from The Jackson Laboratory. Pde1b knockout mice (Pde1b−/−, KO; strain name: B6.129P2-Pde1btm1Dgen/Mmnc) were procured from MMRC through the University of North Carolina, Chapel Hill. Knockout was achieved via insertion of a neomycin resistance cassette into exon 3, disrupting the coding region. Genotyping was performed by PCR using the following primers (5’ to 3’): Forward: AGTTTTCTGGAGGCCTTGGAGACAG; Reverse: CCCCATTGCTCACTTGGTCTGAATG; Neomycin: GGGTGGGATTAGATAAATGCCTGCTCT. Both male and female mice were used for in vivo studies and as a source of bone marrow, spleen and lymph nodes for in vitro and ex vivo experiments. All animal experiments were conducted in accordance with institutional guidelines and were approved by the Institutional Animal Care and Use Committee (IACUC) of the University of Minnesota under approved protocol no. IACUC_2502-42757. All procedures complied with national and institutional regulations governing the care and use of laboratory animals.

### Antibodies and bioreagents

The following antibodies and respective clones purchased from BioLegend were used for staining and/or depletion: CD11c (N418), MHC class II (M5/114/15.2), CD24 (M1/69, 30-F1), CD172a (P84), IRF8 (V3GYWCH), F4/80 (BM8), IRF4 (3E4), CX3CR1 (SA011F11), CD11b (M70), CD64 (X54-5/7.1), Mertk (2b10c42), B220 (RA3-6B2), CD19 (4B12), IgD (11-26 c.2a), CD38 (90), CD44 (IM7), TCRβ (H57-597), CD3 (17A2), CD4 (RM4-5), CD8 (53-6.7), SiglecH (eBio440C), Ly6G (IA8), phospho-CREB1 (Ser133) (A22026B). The following antibodies were purchased from Abcam: Pde1b (EPR12293(2)). The following antibodies were purchased from Thermo Fisher Scientific (Waltham, MA, USA): phospho-GSK3β (1C9E2). 2W-I-Ab tetramer was received as a gift from Marc Jenkins and T. Dileepan. H2kb_SIINFEKL_pMHC tetramer received from NIH Tetramer Core Facility. CFSE labeling dye (Thermo Fisher Scientific, Waltham, MA, USA) was utilized to stain T cells for proliferation studies.

### Single-cell RNA sequencing

Single-cell RNA sequencing (scRNA-seq) was performed on spleen samples from 10-week-old Pde1b−/− and WT mice (*n* = 4 per group). Tissues were dissociated into single-cell suspensions using the GentleMACS Dissociator (Miltenyi, Germany), with red blood cells removed by ACK lysis. Live cells were processed for 5’ scRNA-seq, targeting ~25,000 cells per sample at a sequencing depth of 50,000 reads per cell using the Chromium Single Cell Controller (10x Genomics) at the University of Minnesota Genome Center (UMGC) Single Cell Platform. Libraries were prepared using Chromium GEM-X Single Cell 5’ chemistry (10x Genomics), pooled, and sequenced on an Illumina NovaSeq 6000 platform. Raw base call files were processed, demultiplexed, and aligned to the mouse reference genome (mm10-2020-A) using Cell Ranger (10x Genomics). Resulting gene-cell matrices were analyzed in Seurat v5 (R v4.4.1) (Hao et al, 2024). Cells were subjected to standard quality control filtering to remove low-quality cells and potential doublets based on transcript abundance, gene complexity and mitochondrial content. Data were normalized using SCTransform with glmGamPoi, and variable features were used for principal component analysis (PCA). To minimize confounding from adaptive immune receptor diversity, immunoglobulin and T-cell receptor genes were excluded from dimensionality reduction. Batch effects across biological replicates were corrected using Seurat integration based on canonical correlation analysis (CCA), and integrated embeddings were used for downstream clustering and visualization. Cell populations were identified using shared nearest-neighbor graph construction followed by Louvain clustering and visualized using Uniform Manifold Approximation and Projection (UMAP). Automated cell type annotation was performed using SingleR with the ImmGen reference dataset, and assignments were refined by manual curation using canonical marker gene expression. Differential gene expression analyses were performed using a pseudobulk framework in which counts were aggregated by biological replicate and condition, followed by DESeq2-based testing implemented in Seurat (Love et al, 2014). Cluster-specific marker genes were identified using FindAllMarkers (Wilcoxon rank-sum test). Visualization of gene expression was performed using SCTransform-normalized values. RNA-seq data have been deposited in GEO under accession number GSE297611 (https://www.ncbi.nlm.nih.gov/geo/query/acc.cgi?acc=GSE297611).

### In vitro generation of bone marrow-derived macrophages and Flt3L-generated dendritic cells

Bone marrow cells derived from murine tibias and femurs were cultured with 10 ng/mL of recombinant mouse Flt3L in IMDM supplemented with Pen/Strep and 10% fetal bovine serum (FBS) to generate DC. After 8–10 days of in vitro culture, cells were harvested for in vitro studies or further labeled with fluorochrome-labeled antibodies to CD24, CD172a, CD11c, and MHC class II to purify by FACS prior to in vitro culture studies or magnetic sorting using CD11c ultrapure magnetic beads by Miltenyi. Bone marrow cells cultured with 25 ng/mL of murine M-CSF in RPMI supplemented with Pen/Strep and 10% FBS generated macrophages.

### Isolation of splenic-derived dendritic cells and macrophages

Mouse spleens were harvested prior to dissociation into a single-cell suspension using the GentleMACS dissociator (Miltenyi, Germany). Erythrocytes were then removed through the use of ACK lysis buffer. FACS-sorting using the BD FACS Jazz (BD Biosciences, San Jose, USA) was employed to isolate CD11c+MHC class II + CD24+CD172a^neg^ cDC1, CD11c+MHC class II+CD24^neg^CD172a + cDC2, or F4/80+CD11b^lo^CD64+CD11c^neg^ subsets from total splenocytes. The sorting approach utilized FSC-H vs FSC-A to discriminate out doublets and Live/Dead fixable dyes to gate on live populations. The procedure yielded greater than ~97% purity, as corroborated by flow cytometric analyses. Cells were then utilized for in vitro T cell priming, ex vivo pathogen-infection studies and measurements of cyclic nucleotides and protein kinase activities.

### Pathogen challenge in vivo experiments

*Listeria monocytogenes* strain ΔactA-LM-2W and the 2W:I-Ab tetramers were kindly provided by Drs. Marc Jenkins and Thamotharampillai Dileepan (University of Minnesota, Minneapolis, MN). This LM strain has been shown to cause transient infection after intravenous (i.v.) injection, strong proliferation of responder CD4+ T cells, and subsequent rapid clearance from the system. Pde1b−/− mice at 8–12 weeks of age were injected intravenously with 1 × 10^7^ LM resuspended in 150 µL of PBS. This dose corresponds to a sublethal systemic infection that permits quantification of bacterial clearance and adaptive immune responses without mortality during the experimental window. Inocula were prepared from log-phase cultures and confirmed by plating to verify input CFU. Four to six days post-infection, animals were sacrificed to harvest the spleen and draining lymph nodes (dLN). Tissues were then processed into a single-cell suspension, and erythrocytes were removed prior to staining with 2W:I-Ab tetramers and antibodies. In some experiments, magnetic bead isolation using antibodies targeting the tetramer fluorochrome was employed to enrich tetramer-bound LM-specific T cells, as previously described (Dileepan et al, 2021; Kotov and Jenkins, 2019). Cells were then analyzed by standard flow cytometric analysis for frequency of 2W:I-Ab specific CD4+ T cells.

### Adoptive transfer of dendritic cells in vivo

DC were isolated from spleens of WT or Pde1b−/− mice by fluorescence-activated cell sorting (FACS) of CD11c+MHC class II+ cells, with further enrichment of cDC1 populations defined as CD24+CD172a^neg^ subsets where indicated. Sorted populations had >95% purity. Recipient Pde1b−/− mice (8–12 weeks of age) received 2 × 10^5^ sorted cDC in 100 µl via intravenous (i.v.) injection. 24 h after adoptive transfer, mice were infected intravenously with LM expressing ovalbumin (LM-OVA). Transferred cDC were not pre-loaded with antigen ex vivo, allowing antigen acquisition and presentation to occur in vivo during infection. Spleens were harvested 4–6 days post-infection, and antigen-specific CD8+ T cell responses were quantified by flow cytometry.

### Bacterial burden quantification

Spleens were aseptically harvested from infected mice and homogenized in sterile PBS. Serial dilutions of homogenates were plated on brain heart infusion (BHI) agar plates and incubated at 37 °C overnight. Colony-forming units (CFU) were counted the following day and normalized to whole spleen homogenate per animal.

### Isolation, activation and proliferation of T cells by dendritic cells in vitro

Naive T cells derived from OT mice were isolated using negative depletion approaches through magnetic columns (Miltenyi, Germany). Briefly, spleen and lymph nodes from either OT-I or OT-II mice were isolated. Tissues were subjected to the GentleMACS dissociator (Miltenyi, Germany) to generate single-cell suspensions. Next, OT-II-derived cells were labeled with antibodies to CD8 and MHC class II to deplete the subsets to isolate CD4 + T cells. Similarly, antibodies to CD4 and MHC class II were used to purify the CD8+ T cells in the OT-I model. These cells then served as responders. To prime these responder naive T cells, Flt3L-generated DC were cultured with HKLM encoding ovalbumin. In select studies, DC were FACS-sorted to isolate the CD24+CD172a^neg^ cDC1 and CD24^neg^CD172a + cDC2 subsets. After 5 h of in vitro culture and successive washing, HKLM-fed DC were used to prime CFSE-labeled T cells for 3–4 days in vitro. Proliferation capacity was then measured by monitoring fluorescence intensity dilutions by flow cytometric analysis. In parallel experiments, RNA was isolated for gene expression analysis, and the supernatant was collected for cytokine assessment by ELISA and Luminex Multiplex.

### Pharmacologic inhibition of PDE

Total splenocytes, DC or macrophages were isolated from the mice spleen and immediately plated in 96-well plates at equal density in complete RPMI. To assess isoform-specific contributions of phosphodiesterases to cyclic nucleotide signaling, cells were pretreated for 30–60 min with pharmacologic inhibitors prior to stimulation. The following inhibitors were used: DSR-141562 (Pde1b-preferring; 45 and 150 nM), ITI-214 (pan-PDE1; 10 and 30 nM), roflumilast (PDE4; 1 and 3 nM) and PDE7-IN-2 (PDE7; 1 and 3 µM). Concentrations were based on reported IC₅₀ values. Final DMSO concentration was ≤0.1% in all conditions. Cells were subsequently stimulated with high-dose HKLM or infected with live LM. Cells were harvested 6-8 h post-stimulation for RNA isolation or 18–24 h for supernatant collection and subsequent ELISA measurements.

### Flow cytometric analysis and fluorescence-activated cell sorting (FACS)

Cells were prepared for staining in 0.5% BSA and 2 mM EDTA in PBS with NaN3. Fluorochrome-labeled antibodies were utilized for extracellular staining. For intracellular antibody labeling, cells were fixed in 3% PFA prior to permeabilizing with 0.15% saponin in PBS and staining with primary and secondary antibodies. For phosphoprotein staining, cells were fixed and permeabilized using True-Phos Perm Buffer (BioLegend), following manufacturer instructions to preserve phosphorylation states prior to antibody staining. Cells were analyzed using the BD FACS Lyric flow cytometric analyzer (BD Biosciences, USA) and datasets evaluated using FlowJo (FlowJo LLC, BD Biosciences, USA). All gates were established using unstained and isotype controls. For fluorescence-activated cell sorting (FACS), isolated single cell suspensions were prepared for staining with 1% BSA and 2 mM EDTA in PBS. Cells were stained with antibodies for 20 min prior to washing. Labeled cells were then sorted using the BD FACSJazz flow cytometric sorter (BD Biosciences, USA).

### Real-time quantitative PCR (qPCR)

To analyze gene expression, total RNA was isolated from cells using Trizol (Thermo Fisher Scientific, Waltham, USA) and reverse transcribed into cDNA using Veriti Thermocycler (Thermo Fisher Scientific, Waltham, USA). For qPCR, TaqMan Gene Expression Fast Advanced Master Mix and Gene Expression Assay kits were used to quantify expression levels. Taqman probes to Pde1b (Mm00476643_m1), Irf8 (Mm00492567_m1), Irf4 (Mm00516431_m1), Adgre1 (Mm00802529_m1), Ly6c (Mm03009946_m1), *Listeria monocytogenes* (Ba07921936_s1, Ba07922252_s1), Nos2 (Mm00440502_m1), Arg1 (Mm00475988_m1), Il6 (Mm00446190_m1), Tnfa (Mm00443258_m1), Il12a (Mm00434169_m1), Il10 (Mm01288386_m1), Tgfb1 (Mm01178820_m1), Il17a (Mm00439618_m1), Ccr9 (Mm02528165_s1), Cd74 (Mm00658576_m1), and Creb1 (Mm00501607_m1). For normalization, housekeeping probes used in the qPCR multiplex assays included GAPDH (Mm99999915_g1) and ACTB (Mm02619580_g1). All probes ordered from Thermo Fisher Scientific (Waltham, MA, USA). Samples were acquired and analyzed using the QuantStudio 6 qPCR instrument (Thermo Fisher Scientific, Waltham, MA). Expression levels of target transcripts were calculated by the comparative Ct method (2–ΔΔCt formula).

### Luminex and ELISA assays

Luminex was used for multiplex quantitation of supernatants from cell cultures. For long-term storage, supernatants were collected and stored at −80 °C prior to analyses. The Multiplex Luminex Protein Assay (Thermo Fisher Scientific, USA) kit for detecting IFNγ, IL-1β, IL-2, IL-4, IL-6, IL-10, IL-12p70, IL-17A (CTLA-8), IL-22, TGFβ1, and TNFɑ. Procedures followed manufacturer-recommended protocols. To corroborate multiplex analyses, singleplex ELISA assays were performed to detect IL-6, IL1β, IFNγ, IL-12p70, IL-10, TGFβ, and TNFɑ (R&D Systems; BD Biosciences).

### Cytokine, protein kinase, and cGMP/cAMP immunoassays

Levels of cAMP and cGMP were measured using an ELISA kit in accordance with the manufacturer's instructions (Enzo Life Science, Farmingdale, NY, USA; Abcam, Boston, MA, USA). For steady-state analyses, FACS-sorted splenic cDC1, cDC2, and macrophage populations were used. For pharmacologic inhibition studies, splenocytes or macrophages were treated with indicated inhibitors in the presence or absence of HKLM stimulation. Intracellular cAMP levels were then measured at defined time points following treatment. PKA and PKG activities were quantified using colorimetric kinase activity assays (Thermo Fisher Scientific, USA) and normalized to total protein content in each sample to account for variation in cell number and extraction efficiency.

### Western blotting

Protein lysates were generated using NP-40 lysis buffer supplemented with protease inhibitor cocktail (Thermo Fisher, USA) for protein extraction prior to running on a 4–12% SDS-PAGE gel. Protein was then wet transferred to a nitrocellulose blot, blocked in 5% dry milk and subsequently probed with primary and secondary fluorescent-tagged antibodies diluted in TBS-Tween. Blot images were captured using the Licor Odyssey (Licor Biosciences, Lincoln, Nebraska, USA). Quantitative analyses were performed using the ImageStudio software (Licor Biosciences). Band intensities were quantified using the densitometric analysis function by measuring the integrated pixel intensity of target protein and loading control bands in separate fluorescence channels, and target protein expression was normalized to the loading control (i.e. GAPDH, Vinculin) by calculating the ratio of target band intensity to the corresponding loading control intensity.

### Statistics and reproducibility analyses

All experiments were performed using biological replicates across at least three separate animal cohorts, unless otherwise indicated in the figure legends. Where applicable, technical replicates (such as qPCR duplicates or triplicates) were performed within assays to ensure measurement accuracy. Statistical analyses were performed using two-tailed unpaired Student’s *t*-tests. These tests assume approximate normal distribution of the data and similar variance between groups; given the sample sizes and consistency with standard practice in the field, these assumptions were not formally tested. Variance between groups was comparable based on the similar dispersion of data points. Data were presented as mean ± SEM, as indicated in the figure legends. Statistical significance is denoted as *p* < 0.05 (*), *p* < 0.01 (**), and ns (not significant); exact *p* values are provided in the figures. No formal sample size calculations were performed. Sample sizes were selected based on prior experience with similar experimental systems and are consistent with standard practices in the field. Predefined exclusion criteria included failed infection, unsuccessful intravenous (i.v.) cell transfer, or technical issues during tissue processing. All exclusions are reported in the corresponding figure legends. No data points were excluded unless meeting these criteria. Investigators were not blinded to group allocation during experiments or outcome assessment. No formal randomization procedure was used; animals were assigned to experimental groups based on genotype and handled identically across conditions to minimize potential bias. This study was conducted and reported in accordance with the ARRIVE guidelines for animal research.

### Use of AI-assisted technologies

During the preparation of this work, the authors used AI-assisted technologies to assist with condensing and refining the discussion section and the figure legends for clarity and conciseness. Following this assistance, all content was carefully reviewed and edited to ensure accuracy and consistency. Authors take full responsibility for all content of the publication.

## Supplementary information

Peer Review File

Source data Fig. 1

Source data Fig. 2

Source data Fig. 3

Source data Fig. 4

Source data Fig. 5

Source data Fig. 6

Source data Fig. 7

Source data Fig. 8

Source data Fig. 9

Expanded View Figures

## Data Availability

Single-cell RNA-seq data generated in this study have been deposited in the Gene Expression Omnibus database under accession number GSE297611 (https://www.ncbi.nlm.nih.gov/geo/query/acc.cgi?acc=GSE297611). The source data of this paper are collected in the following database record: biostudies:S-SCDT-10_1038-S44319-026-00924-z.
