# Supplementary material for: Pde1b controls cyclic nucleotide signaling in cDC1 and macrophages to regulate immune responses
Source: EMBO Rep. 2026 Sep 15;27(20):5904–26. doi: 10.1038/s44319-026-00924-z (PMC13694280; doi:10.1038/s44319-026-00924-z)
Supplement: Supplementary file 1 [file 44319_2026_924_MOESM11_ESM.pdf]

Expanded View Figures

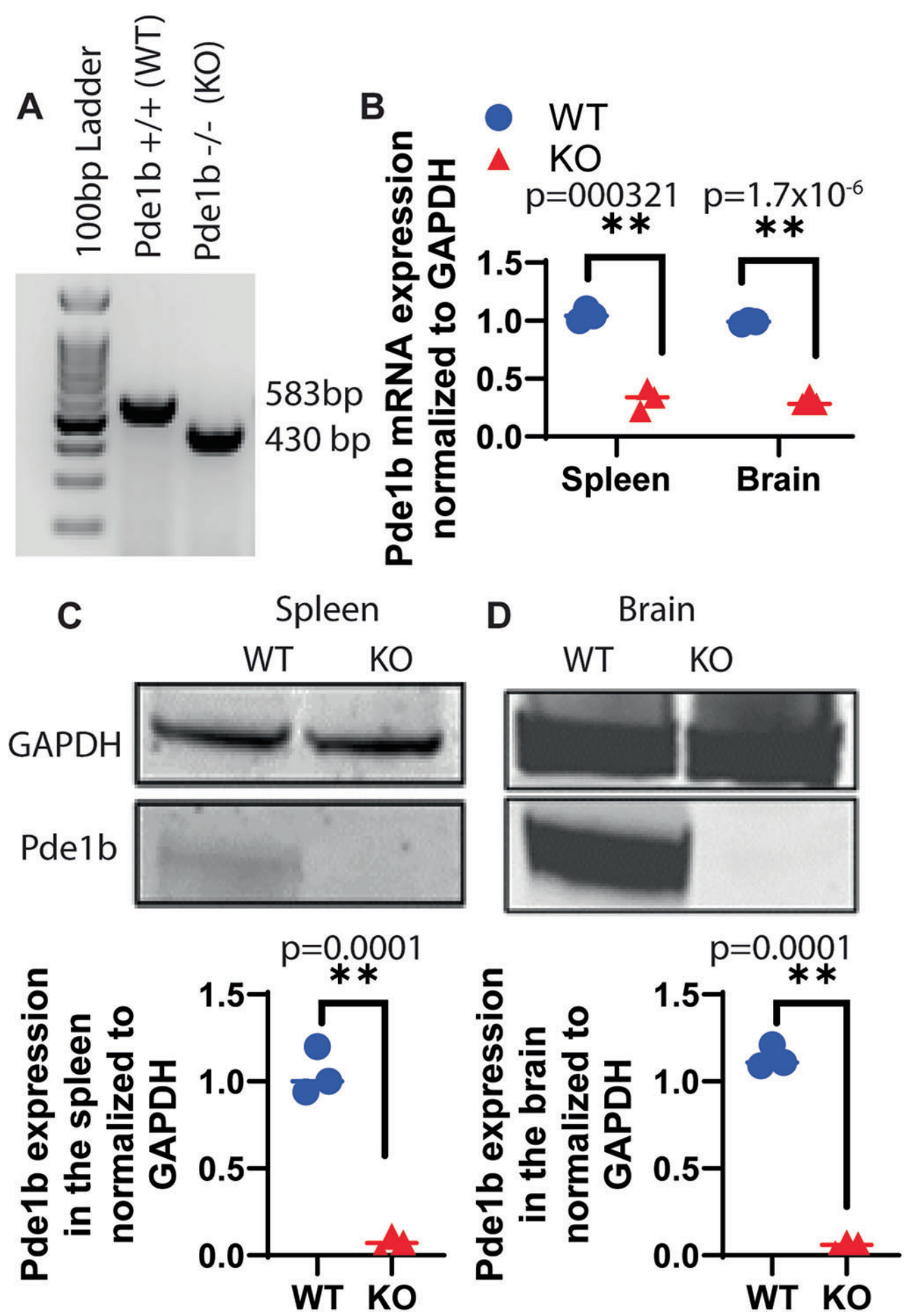

Figure EV1. Validation of the Pde1b global knockout mouse.

(**A**) Genotyping PCR of Pde1b−/− and WT mice, showing genomic DNA band sizes for the targeted Pde1b locus. (**B**) Quantitative PCR analysis of Pde1b mRNA expression in spleen and brain, normalized to beta actin. Data shown as a mean ± SEM from three biological replicates. (**C**, **D**) Western blot analysis of Pde1b protein in spleen and brain tissues from Pde1b−/− and WT mice. Protein levels were normalized to their respective endogenous control. Bar graphs show quantification of Pde1b protein expression relative to GAPDH. Data shown as a mean ± SEM from three biological replicates. Statistical analysis among group conditions was performed using an unpaired Student's *t*-test. *$p < 0.05$, **$p < 0.01$ and ns not significant; numerical *p* values for statistically significant comparisons are shown above indicated groups within the graphs where available.

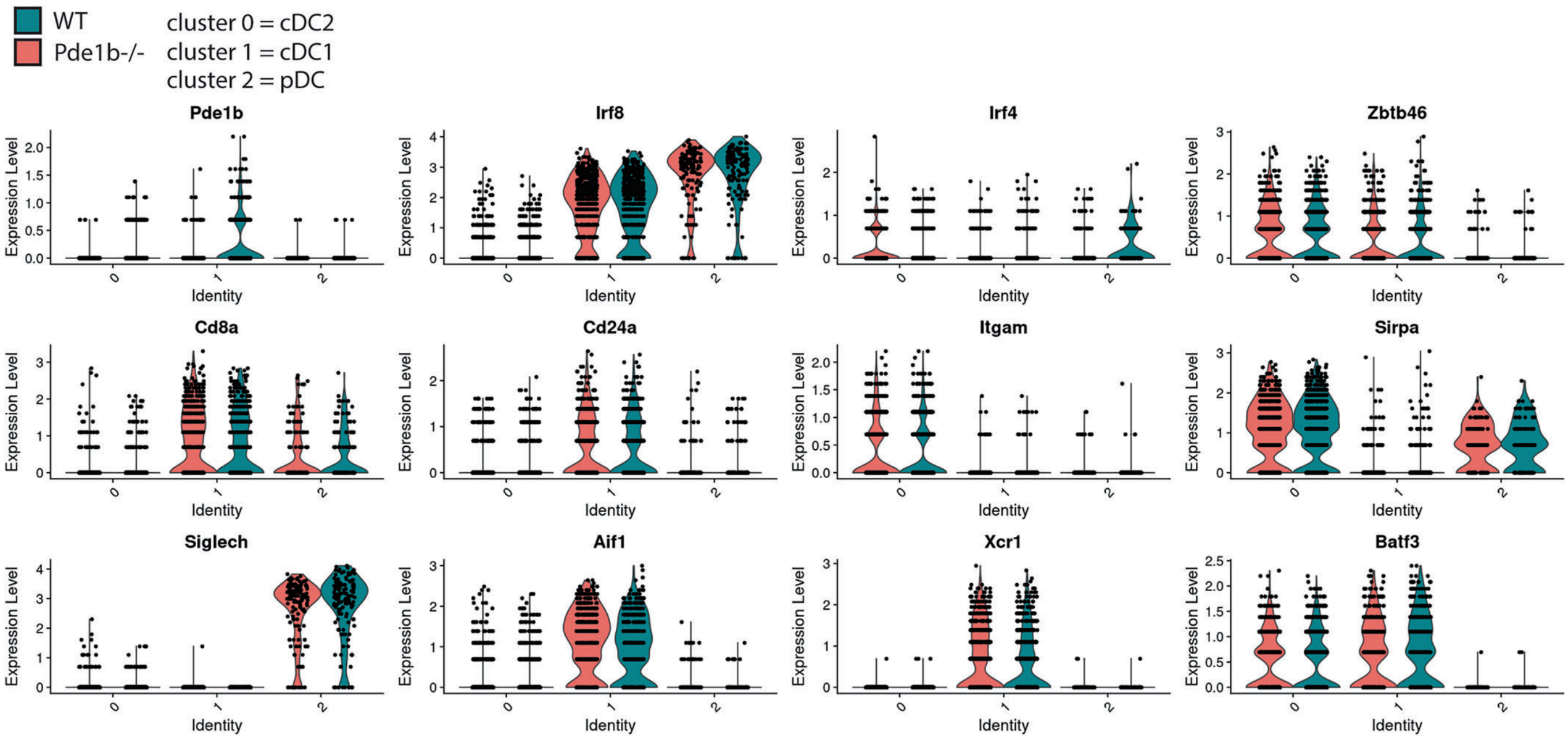

Figure EV2. Identification of DC subsets using lineage-specific markers.

scRNA-seq analysis ($n = 4$ mice per genotype) was used to probe Irf8, Irf4, Zbtb46, Cd8a, Cd24a, Itgam, Sirpa (CD172a), Siglech, Aif1, Xcr1, and Batf3 in the distinct DC subclusters to determine cDC2, cDC1, and pDC subsets. Cluster 0 = cDC2, cluster 1 = cDC1, and cluster 2 = pDC.

 © The Author(s)

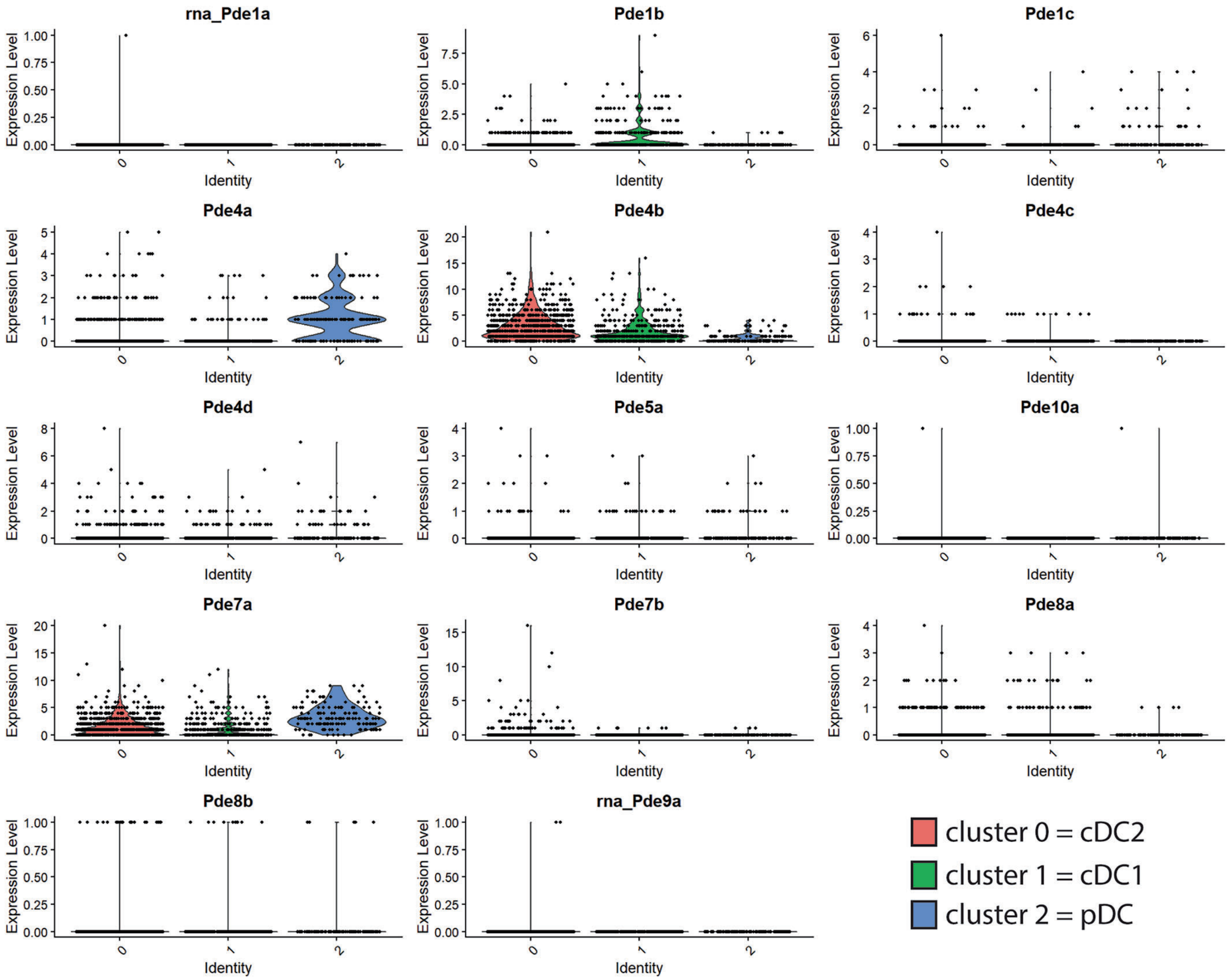

Figure EV3. Expression of phosphodiesterase family members in dendritic cell subsets.

Single-cell RNA sequencing (scRNA-seq) analysis of DC subset clusters showing expression of Pde1a, Pde1b, Pde1c, Pde4a, Pde4b, Pde4c, Pde4d, Pde5a, Pde7a, Pde7b, Pde8a, Pde8b, Pde9a, and Pde10a (*n* = 4 mice per genotype). Expression profiles are presented as violin plots. Data shown as mean from integrated biological replicates.

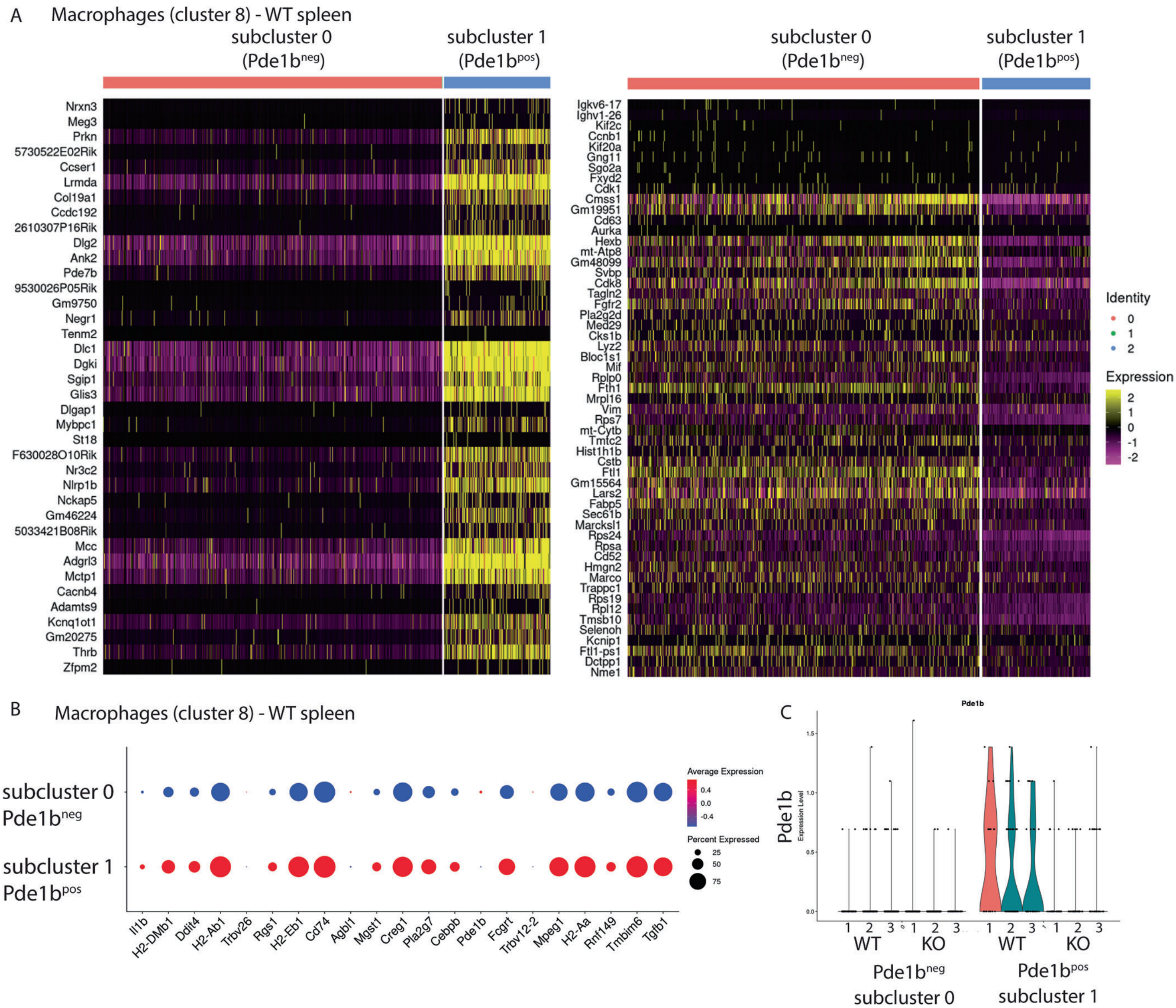

Figure EV4. Macrophage (cluster 8) differential gene expression.

(**A**) Heatmap table from single-cell RNA sequencing (scRNA-seq) showing differentially expressed genes (DEGs) between subcluster 0 (Pde1b-negative) and subcluster 1 (Pde1b-positive) within the parent macrophage cluster 8 of WT samples ($n = 4$ mice per genotype). (**B**) Dot plot illustrating frequency and expression levels of top DEG in subcluster 0 and subcluster 1 of WT splenic macrophages ($n = 4$ mice per genotype). (**C**) Analysis of biological replicates for uniformity in identification and expression of Pde1b in the unique Pde1b-positive subcluster, but not the Pde1b-negative cohort ($n = 4$ mice per genotype).

 © The Author(s)

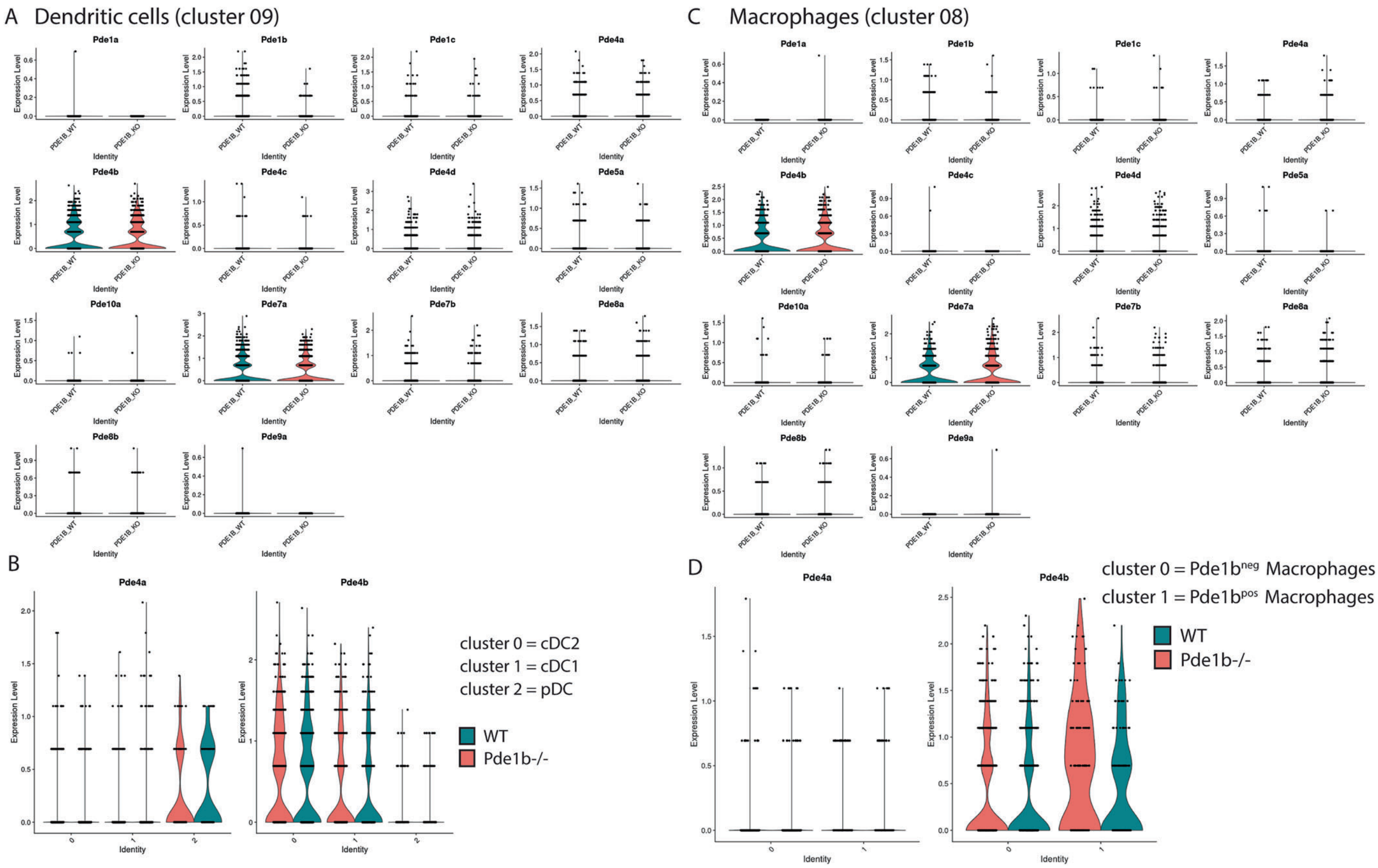

Figure EV5. Lack of compensatory upregulation of PDE isoforms in Pde1b-deficient mice.

Expression levels of PDE isoforms in (**A**) total DC cluster 9 and of (**B**) Pde4a and Pde4b within distinct DC subclusters analyzed from scRNA-seq data in WT and Pde1b −/− mice ($n = 4$ mice per genotype). Expression levels of PDE isoforms in (**C**) total macrophage cluster 8 and of (**D**) Pde4a and Pde4b within distinct macrophage subclusters analyzed from scRNA-seq data in WT and Pde1b−/− mice ($n = 4$ mice per genotype). Data shown as mean from integrated biological replicates.
