# Supplementary material for: Pde1b controls cyclic nucleotide signaling in cDC1 and macrophages to regulate immune responses
Source: EMBO Rep. 2026 Sep 15;27(20):5904–26. doi: 10.1038/s44319-026-00924-z (PMC13694280; doi:10.1038/s44319-026-00924-z)
Supplement: Supplementary file 2 [file 44319_2026_924_MOESM1_ESM.pdf]

EMBO reports

Pde1b controls cyclic nucleotide signaling in cDC1 and macrophages to regulate immune responses

Lais Rekowsky, Ricardo da Silva, Jonathan Seenarine, Ayane Resende, Marissa Macchietto, Tatiana de Moura, Diana Elizondo, and Michael Lipscomb

Corresponding author(s): Michael Lipscomb (lips0046@umn.edu)

Review Timeline:

Submission Date:	10th Sep 25
Editorial Decision:	10th Dec 25
Revision Received:	17th May 26
Editorial Decision:	6th Aug 26
Revision Received:	11th Aug 26
Accepted:	25th Aug 26

Editor: Martina Rembold / Achim Breiling

Transaction Report:

(Note: With the exception of the correction of typographical or spelling errors that could be a source of ambiguity, letters and reports are not edited. Depending on transfer agreements, referee reports obtained elsewhere may or may not be included in this compilation. Referee reports are anonymous unless the Referee chooses to sign their reports.)

1st Editorial Decision **10th Dec 2025**

Dear Dr. Lipscomb

Thank you for the submission of your research manuscript to our journal. I apologize for the delay in handling your manuscript. We have meanwhile received the full set of referee reports that is copied below and I have also discussed the reports further with the referees.

As you will see, the referees consider the findings potentially interesting but I notice a number of significant concerns that are largely shared by all referees:

- One relates to potential compensatory effects of other PDEs, which could e.g. be addressed with more expression analysis or acute inhibitor treatment.
- Other concerns relate to cell type specificity, whether Pde1b KO mice display more severe disease symptoms when infected with Listeria, or to the absence of rescue experiments.
- Another important concern is the number of replicates for the scRNA-seq experiments. This point seems important as many conclusions rely on it.

I have discussed the referee reports and the points noted above further with the referees.
Based on their feedback, I suggest the following revisions:
- Please add 1 or 2 more replicates for the scRNA-seq to strenghten these data
- Please mine your existing datasets for compensatory expression of other PDEs
- Use acute inhibitor treatments to verify the effects observed in the global PDE1b KO model and to test for potential compensation by other PDEs
- For the in vivo inhibitor, rescue experiments and to test for cell type specificity, referee #2 and #3 suggested using specific cKO mice lacking cDC1 (e.g., BATF3-/- or XCR-DTR) to differentiate between the role of cDC1 and macrophages and/or an adoptive transfer of WT Pde1b expressing cells into the Pde1b KO model (referee #3, point 3). This could help to discriminate between Pde1b's role in cDC1 and macrophages.
- The analysis of a Pde1b cKO mouse model is not required.

I hope this outline makes sense. Please let me know in case you disagree, and we can discuss the exact revision requirements further, also in a video chat, if you like.

Please address all referee concerns in a complete point-by-point response. Acceptance of the manuscript will depend on a positive outcome of a second round of review. It is EMBO Reports policy to allow a single round of revision only and acceptance or rejection of the manuscript will therefore depend on the completeness of your responses included in the next, final version of the manuscript.

We realize that it is difficult to revise to a specific deadline. In the interest of protecting the conceptual advance provided by the work, we recommend a revision within 3 months (March 10th, 2026. I realize that more time might be required for the revision and I am open to discuss the timeline in more detail.

==

When submitting your revised manuscript, we will require:

1) a .docx formatted version of the manuscript text (including legends for main figures, EV figures and tables). Please make sure that the changes are highlighted to be clearly visible.

2) individual production quality figure files as .eps, .tif, .jpg (one file per figure).
Please download our Figure Preparation Guidelines (figure preparation pdf) from our Author Guidelines pages https://www.embopress.org/page/journal/14693178/authorguide for more info on how to prepare your figures.

3) a .docx formatted letter INCLUDING the reviewers' reports and your detailed point-by-point responses to their comments. As part of the EMBO Press transparent editorial process, the point-by-point response is part of the Review Process File (RPF), which will be published alongside your paper.

4) a complete author checklist, which you can download from our author guidelines (<https://www.embopress.org/page/journal/14693178/authorguide>). Please insert information in the checklist that is also reflected in the manuscript. The completed author checklist will also be part of the RPF.

5) Please note that all corresponding authors are required to supply an ORCID ID for their name upon submission of a revised manuscript (<https://orcid.org/>). Please find instructions on how to link your ORCID ID to your account in our manuscript

tracking system in our Author guidelines
(<https://www.embopress.org/page/journal/14693178/authorguide#authorshipguidelines>)

6) We replaced Supplementary Information with Expanded View (EV) Figures and Tables that are collapsible/expandable online. A maximum of 5 EV Figures can be typeset. EV Figures should be cited as 'Figure EV1, Figure EV2" etc... in the text and their respective legends should be included in the main text after the legends of regular figures.

- For the figures that you do NOT wish to display as Expanded View figures, they should be bundled together with their legends in a single PDF file called *Appendix*, which should start with a short Table of Content. Appendix figures should be referred to in the main text as: "Appendix Figure S1, Appendix Figure S2" etc. See detailed instructions regarding expanded view here:
<https://www.embopress.org/page/journal/14693178/authorguide#expandedview>

- Additional Tables/Datasets should be labeled and referred to as Table EV1, Dataset EV1, etc. Legends have to be provided in a separate tab in case of .xls files. Alternatively, the legend can be supplied as a separate text file (README) and zipped together with the Table/Dataset file.

7) Please include a dedicated "Data Availability" section at the end of the Methods (suggested wording: "The [structural coordinates | microarray | mass spectrometry] data from this publication have been deposited to the [name of the database] database [URL] and assigned the identifier [accession | permalink | hashtag]."). Should this not apply, this should still be stated as "This study includes no data deposited in external repositories."

8) At EMBO Press we ask authors to provide source data for the main manuscript figures. You will receive a separate email with instructions for providing source data with your revised manuscript, including how to upload and organize the files.

Additional information on source data and instruction on how to label the files are available
<https://www.embopress.org/page/journal/14693178/authorguide#sourcedata>

9) The journal requires a statement specifying whether or not authors have competing interests (defined as all potential or actual interests that could be perceived to influence the presentation or interpretation of an article). In case of competing interests, this must be specified in your disclosure statement. Further information: https://www.embopress.org/competing-interests

10) Figure legends and data quantification:
The following points must be specified in each figure legend:

- the name of the statistical test used to generate error bars and P values,
- the EXACT p-values,
- the number (n) of independent experiments (please specify technical or biological replicates) underlying each data point,
- the nature of the bars and error bars (s.d., s.e.m.)

- If the data are obtained from n {less than or equal to} 5, show the individual data points in addition to the SD or SEM.
- If the data are obtained from n {less than or equal to} 2, use scatter blots showing the individual data points.

Discussion of statistical methodology can be reported in the materials and methods section, but figure legends should contain a basic description of n, P and the test applied.

See also the guidelines for figure legend preparation:
https://www.embopress.org/page/journal/14693178/authorguide#figureformat

- Please also include scale bars in all microscopy images and define their size in the legend, not the image.

11) Our journal encourages inclusion of *data citations in the reference list* to directly cite datasets that were re-used and obtained from public databases. Data citations in the article text are distinct from normal bibliographical citations and should directly link to the database records from which the data can be accessed. In the main text, data citations are formatted as follows: "Data ref: Smith et al, 2001" or "Data ref: NCBI Sequence Read Archive PRJNA342805, 2017". In the Reference list, data citations must be labeled with "[DATASET]". A data reference must provide the database name, accession number/identifiers and a resolvable link to the landing page from which the data can be accessed at the end of the reference. Further instructions are available at <https://www.embopress.org/page/journal/14693178/authorguide#referencesformat>.

12) All Materials and Methods need to be described in the main text using our 'Structured Methods' format. According to this format, the Methods section includes a Reagents and Tools Table (listing key reagents, experimental models, software and relevant equipment and including their sources and relevant identifiers) followed by a Methods and Protocols section describing the methods, ideally using a step-by-step protocol format. The aim is to facilitate adoption of the methodologies across labs. Please download and fill our Reagents and Tools Table template (.docx), which you can find in our author guidelines:
https://www.embopress.org/page/journal/14693178/authorguide#structuredmethods.

When submitting your revised manuscript, please do not include the Reagents and Tools Table in the Methods section of the manuscript but upload it as a separate file choosing the file type "Reagent Table".
An example of a Method paper with Structured Methods can be found here:
https://www.embopress.org/doi/10.15252/msb.20178071.

13) As part of the EMBO publication's Transparent Editorial Process, EMBO Reports publishes online a Review Process File to accompany accepted manuscripts. This File will be published in conjunction with your paper and will include the referee reports, your point-by-point response and all pertinent correspondence relating to the manuscript.

You are able to opt out of this by letting the editorial office know (emboreports@embo.org). If you do opt out, the Review Process File link will point to the following statement: "No Review Process File is available with this article, as the authors have chosen not to make the review process public in this case."

We would also welcome the submission of cover suggestions, or motifs to be used by our Graphics Illustrator in designing a cover.

I look forward to seeing a revised form of your manuscript when it is ready and please let me know if you have questions or comments regarding the revision.

Yours sincerely,

Martina Rembold, PhD
Senior Editor
EMBO reports

====================

Referee #1:

Rekowsky et al. used an impressive portfolio of methods, ranging from single-cell transcriptomic analyses to knockout mouse models and an in vivo infection model, to establish i.) the expression profile of PDE1b and ii.) its putative functions as new lineage-specific marker and regulator of distinct myeloid (sub)lineages regulating e.g. the maturation of DCs (type 1 cDC1) and macrophages (M1-like CD11c+F4/80+CD11blow and others). The authors further present data that suggest an extrinsic influences of PDE1b -expressing myeloid antigen-presenting cells on PDE1b (low or now) lymphocytes and granulocytes. Finally, based on the impaired T-cell priming of PDE1b-deficient APCs, the authors conclude that in vivo infections with an attenuated strain of Listeria monocytogenes are cleared less efficiently.
Overall, the work done appears to be very solid, well presented, and coherently interpreted. I have only a few major points, which the authors should take into account before their manuscript is published:
1.) Suppl. Fig. 2A-D: This is a highly relevant figure that, in my opinion, should be moved to the main part of the paper. According to the transcript analysis I also wonder if a truncated Pde1b protein I still present in the KO samples presented. To assess this, complete gels/membranes need to be provided. Please also clarify for Suppl. Fig. 2B if GAPDH or beta-actin were used to normalize the data. Legend and panel do not match with respect to this point.
2.) On the one hand, as a functional consequence of PDE1b inhibition, and on the other hand, as a prerequisitee for the PDE1b-controlled cGMP (and cAMP) pools, the authors must also address in more detail any of the possible cGMP (such as sGCalpha1, sGCalpha2, sGCbeta1, and NRP1, NRP2) and cAMP generators in the manuscript. I have not found any information on this important aspect in the main text body. Therefore, my question is if any of the aforementioned cyclase / cyclase subunits, in particular those that are prone to produce cGMP, are also detectable, for example, in the PDE1b-positive cDC1or in respective MC sub-lineages?
3.) Even though the authors show that no other PDEs were compensating for the loss of PDE1b, multiple and unexpected regulations during the development of global / constitutive knockout mice need to be taken into account. Thus, it would clearly strengthen the manuscript if the authors could either include a representative set of data from a conditional PDE1b KO lacking this phosphodiesterase e.g. only in the DC or MC cell population. Alternatively, and because I realize that a floxed PDE1b line is not readily available, I wonder what the effect of (acute or chronic) vinpocetine or SCH51866 or lenrispodun treatment on the immune response to LM might be and if any of this compounds that are considered to inhibit PDE1 (compared to other PDEs) rather specifically would mimic e.g. the tolerogenic expression signatures seen in the PDE1b KO.
4.) Fig. 9: The authors should clarify which PKG isoform is present in the cDC1 cells and in macrophages. In this respect the available ssRNAseq data must also be complemented by informative Western blots using specific antibodies for PKG1alpha, PKG1beta and/or PKG2.
5.) The term "metabolic control" used throughout the MS is not clearly defined and not related to altered metabolic functions like

glycolysis and oxidative phosphorylation of the PDE1b proficient versus deficient immune cells.
6.) The authors stated that "Notably, no compensatory increased expression of other phosphodiesterase genes or isoforms was observed in macrophages or DC (Supplemental Figure 3A-3B). Can we deduce from this that in other immune cells such compensation was seen in the PDE1b KO?
7.) I wonder if the colonization of liver, galbladder, bone marrow upon L. monocytogenes infections was altered between PDE1b KO and WT mice? Can the authors report any additional microbiological, histopathological, and clinical parameters that differed in a PDE1b-dependent manner?

Referee #2:

Summary
1. Does this manuscript report a single key finding? YES/NO
Yes, it reports on the effect of knockout of the phosphodiesterase Pde1b.
2. Is the reported work of significance (YES), or does it describe a confirmatory finding or one that has already been documented using other methods or in other organisms etc (NO)? YES/NO
Yes
3. Is it of general interest to the molecular biology community? YES/NO
No, might be rather interesting for the immunology field.
4. Is the single major finding robustly documented using independent lines of experimental evidence (YES), or is it really just a preliminary report requiring significant further data to become convincing, and thus more suited to a longerformat article (NO)? YES/NO
No, the report is mainly about a performed scRNAseq experiment with only two biological replicates.
Report:
In the manuscript "Pde1B is a lineage-specific regulator of cyclic nucleotide signaling that balances immunity", Rekowsky and da Silva et al. report on the effect of knockout of the phosphodiesterase Pde1B. They performed scRNAseq with total spleen and bone marrow cells of wildtype and Pde1b-/- mice. They report that Pde1b is mainly expressed in conventional dendritic cells type 1, macrophages, as well as neutrophils but lacking in all lymphocyte subsets. By comparing mRNA expression between cells from wildtype and Pde1b-/- mice, they show that loss of Pde1b in cDC1, macrophages and neutrophils also affects gene expression in other cell types such as B cells, T cells and NK cells. Overall, knockout of Pde1b leads to a more tolerogenic gene expression in affected by accumulation of cAMP/cGMP. Subsequently, the authors demonstrate reduced T cell responses in a Listeria monocytogenes infection model.
While the concept of Pde1b loss leading to a more tolerogenic phenotype is interesting, the claim is mainly based on differential gene expression in a scRNAseq dataset with only two biological replicates. Further, analysis of flow cytometry and scRNAseq data has some flaws. In the following are my points of concern:
Major comments:
1. Why did the authors use two biological replicates which limits the robustness of the data? Interpretation of changes in frequency as shown in Figure 1B and 6E are difficult to interpret when originating from two replicates. Further, the authors did not report or show whether the described occurred in both replicates.
2. How did the authors choose the log2 fold change used in the different volcano plots throughout the manuscript? When describing the GSEA on page 3, the authors used a logFC > 2. However, for the subsequent analysis they used a log2FC of ~0.2/0.25, which seems quite low and they did not comment on that.
3. The presented gating in different figures seems to be rather involuntarily and rough (e.g. Figure 2I, 3H, 3I, 3J, 4A, 6G, 6H, 8E). The authors should refine their gating strategies or show isotype or FMO controls that were used to set gates.
4. The authors report that other PDEs were not changed but only analyzed expression on the whole DC and macrophage cluster. As they observed expression only in a subset of cells, analysis in Supp. Fig. 3 should be repeated for Pde1b+ cDC1 and Pde1b+ macrophages only.
5. Subclustering of macrophages: Why did the authors exclude the green-labeled cells in Figure 3C?
6. The scRNAseq data of the bone marrow indicates reduced frequencies of MDPs. Did the authors observe reduced frequencies of DCs and monocytes/macrophages outside of the bone marrow in Pde1b-/- mice? If they perform BM cultures and induce differentiation into DCs and monocytes/macrophages, do they observe reduced output in Pde1b-/- mice compared to wt?
7. The authors use a Listeria infection model and show that Listeria-specific CD4+ T cells are reduced in Pde1b-/- mice and provide in vitro data that macrophages have an increased bacterial load. Did the authors observe a more severe disease in Pde1b-/- mice and increased bacterial load in vivo? These data would be important as they show that the observed changes are really relevant.
8. Why did the authors peform co-culture of cDC1 and OT-I T cells after challenge with Lm-OVA? In infected mice, they observed reduced frequencies of Listeria-specific CD4+ T cells not CD8+ T cells. So, they should rather test whether Pde1b-/- cDC1 show reduced capacity to activate OT-II T cells.
9. How do the authors explain this extrinsic effect of knockout of Pde1b? Do DCs and/or macrophages transfer cAMP and cGMP to others cells? Do they produce other tolerogenic mediators?
10. Is the expression of Pde1b conserved between mice and humans? Did the authors check the expression of Pde1b and other isoforms in published bulk- or scRNA datasets? This information is important for a translation of the data to the human system.
Minor comments:

1. In Volcano-Plots: The authors should clearly indicate whether the genes with positive or negative logfc are increased in Pde1b-/- cells.
2. Why do DC and macrophages express less Pde1b in the bone marrow compared to spleen? Is it increased during maturation of the cells? How is the expression in other tissues?
3. The authors report that the expression of Pde1b is higher in spleen compared to thymus. Did they directly compare the expression level in cDC1 and macrophages between spleen and thymus? As thymus mainly consists of T cells, which are shown to be negative for Pde1b, this might be misinterpreted.
4. What do the authors mean with: "Gene set enrichment analysis (GSEA) of differentially expressed genes (DEGs; logFC > 2) against the Molecular Signatures Database revealed significant overlaps with macrophages, DC and mature B cells, as well as notable associations with NK cells, memory T cells and granulocytes (Figure 1E)."
5. Page 4, second paragraph: "depressed frequency" might be rephrased.
6. Page 4, third paragraph: "Flow cytometry showed decreased levels of Cx3CR1+CD11b+ myeloid subpopulations in Pde1b-/- mice, with fewer pro-inflammatory Ly6ChiCD11cneg and more non-classical, patrolling LyC6mid/lo non-classical (patrolling) monocytes, which are anti-inflammatory and involved in tissue-repair, were increased (Supplemental Figure 5D)." Should be rephrased.

Referee #3:

This is an impressive reports which identifies PDE1B as a unique lineage-specific checkpoint regulating immune tolerance. As a dual se´pecific (cAMP/cGMP) phosphodiesterases Pde1b is expressed in cDC1 dendritic cells and a unique macrophage subset. Global Pde1b-deficient mice show suppresses inflammatory responses, impaired antigen-specific T cell priming, and immune tolerance against Listeria infection. This is a nivel and highly exciting finding. However, the report have several weaknesses which shoulb be addressed:

1. To be able to highlight the exclusive role of PDe1b instead of several PDEs, the author should analyze their RNA expression data and and show if Pde1b KO led to compensatory regulation of other Pde1 subfamilies (a, c) and other Pde families (Pde2-11).

2. The whole study relies on the use of just one global KO mouse model where the effects of Pde1b are abrogated chronically. How about more acute effects? There are good PDE1 inhibitiors avaiable, e.g. ITI212 and simal compound which work in vivo. ´Though not selective for Pde1b vs. Pde1c, would they recaputulate the effects of the KO?

3. The paper is missing any kind of "rescue" experiement should that adoptive transfer of WT Pde1b expressing cells or overexpression of Pde1b in KO cell populations can rescue the phenotype.

4. I am not an expert in RNAseq but I noticed that some data e.g shown in Figure 1 are done with just two biological replicates which might be underpowered? In terms of statistics, t-test was used thoughour. However, some of the graphs show multiple comparisons on the same bar graph e.g Fig 7, Fig 8. One should either use a posthoc test for multiple comparisons and show each set of WT vs KO data separately like in Fig 9

Response to Reviewers

We thank the Editor and the reviewers for their very constructive evaluation and thoughtful comments of our manuscript. The employed recommendations have helped clarify our science, streamline our story and strengthened the rigor. In particular, we have focused on the functional and mechanistic value of antigen-presentation, rescue studies, determination of compensatory changes and downstream mechanisms. We believe we were able to successfully address all comments and feedback. Below, we provide point-by-point responses and direct the reviewers to the location of the requested dataset or appropriate edits.

#1 - Reviewer feedback: Potential compensatory regulation of other PDE family members in the global *Pde1b* KO model, and need for orthogonal validation using acute inhibition.

- Reviewer #1: "multiple and unexpected regulations during the development of global / constitutive knockout mice need to be taken into account," and suggested testing acute inhibition to validate whether the phenotype is directly attributable to PDE1 activity
- Reviewer #2: noted that analysis of PDE expression was only performed at the level of whole DC/macrophage clusters, and requested subset-specific analysis (Pde1b^{+} populations).
- Reviewer #3 explicitly asked: "show if Pde1b KO led to compensatory regulation of other Pde1 subfamilies (a, c) and other Pde families (Pde2–11)."

<u>Response</u>: We thank the reviewers for raising the important question of potential compensatory PDE activity and the concern that the phenotype might reflect developmental adaptation in the global knockout. To directly address this, we combined transcriptomic analysis with pharmacological inhibition experiments. scRNAseq and qPCR analyses demonstrate that Pde1a and Pde1c are not detectably expressed in cDC1 or macrophages, consistent with published ImmGen datasets, and there is no compensatory upregulation of other PDE1–11 family members in these populations in Pde1b-deficient cells. To functionally test whether the observed phenotype could be explained by alternative cyclic nucleotide regulatory pathways, we performed acute inhibition experiments using Pde1b-selective, pan-PDE1, PDE4, and PDE7 inhibitors in splenic APC. PDE1 inhibition recaptured the cytokine phenotype observed in Pde1b-deficient cells, whereas PDE7 inhibition had no detectable effect. PDE4 inhibition produced more modest effects and was associated with lower magnitude changes in inflammatory readouts, consistent with a more basal contribution of PDE4 to cyclic nucleotide homeostasis rather than direct regulation of the activation-dependent phenotype. Together, these data indicate that the phenotype is not driven by compensatory PDE expression or developmental adaptation, but reflects a a dominant role of PDE1 activity in select APC subsets to regulate responses. Data is presented in **<u>Supplemental Figure 2 and 4 and in Figure 8</u>**

#2 - Reviewer feedback: insufficient biological replication and statistical robustness of scRNA-seq data.

- Reviewer #2: "only two biological replicates" were used and that interpretation of frequency changes is limited without replicate-level validation.
- Reviewer #3: similarly noted that key conclusions rely on scRNA-seq datasets with limited replication.

Response: scRNAseq and accompanying bioinformatics was performed on additional two WT and two Pde1b-/- KO mice spleen. This brings total biological replicates to four WT compared to four KO. Data was re-analyzed for clustering and differential expression. Results demonstrated reproducibility across replicates, no significant differences observed among all measures of intracohort comparisons. Manuscript methods section and results updated to note replicate handling, clarify how thresholds were selected, and use of log2FC thresholds for pseudobulk. Below is the umap replicates of all eight samples; the four samples on left are WT and four samples on the right are KO. Samples were integrated and analyzed as prior described for pseudobulk analysis in the analytical software package. Source data has been made available.

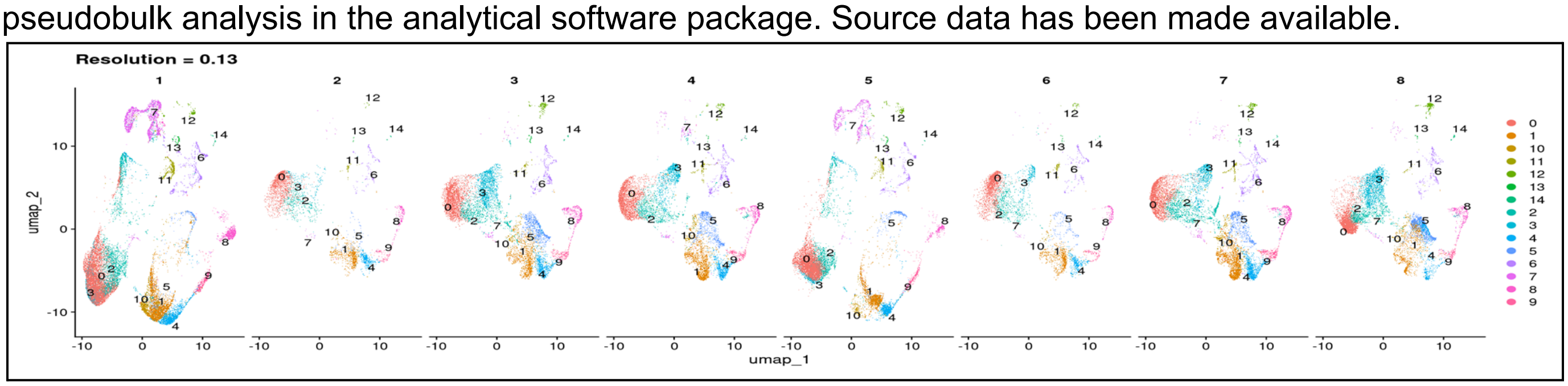

After quality control, we retained three samples per condition. One sample had >50K cells and was excluded following consultation with our bioinformatics specialist and co-author (Dr. Macchietto), as its substantially

higher cell count disproportionately weighed the pseudobulk signal and biased downstream analyses upon inclusion of all four replicates. Removing this sample ensured a more balanced and interpretable comparison across replicates. The remaining samples, as shown below, showed strong reproducibility across conditions. WT spleen replicates (19K, 25K, 28K cells) and KO spleen replicates (17K, 24K, 24K cells) exhibited consistent cellular composition and gene expression patterns.

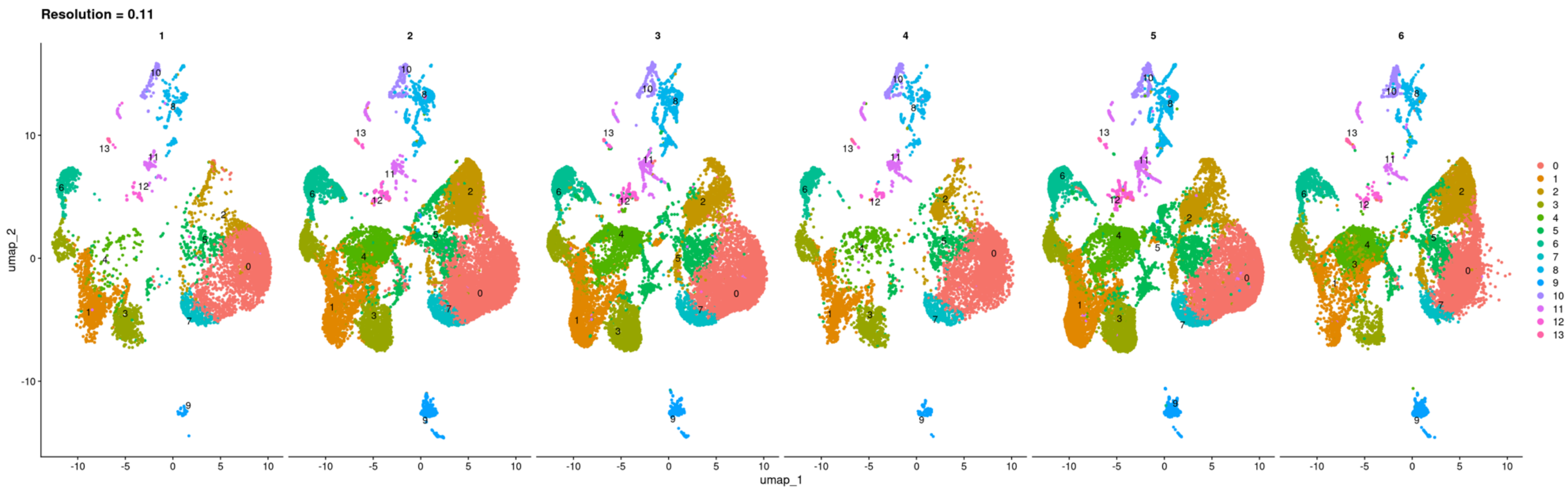

To further validate reproducibility, we examined lineage-defining genes at the single-cell level and confirmed concordance with the pseudobulk analysis. For example, Hmox1 expression, which is specifically enriched in macrophages under homeostatic conditions, was consistently restricted to the macrophage cluster (cluster 11) across all replicates in both WT and KO groups. The figure below shows Hmox expression as feature plot; 3 groups on the left are WT and 3 groups on the right are KO.

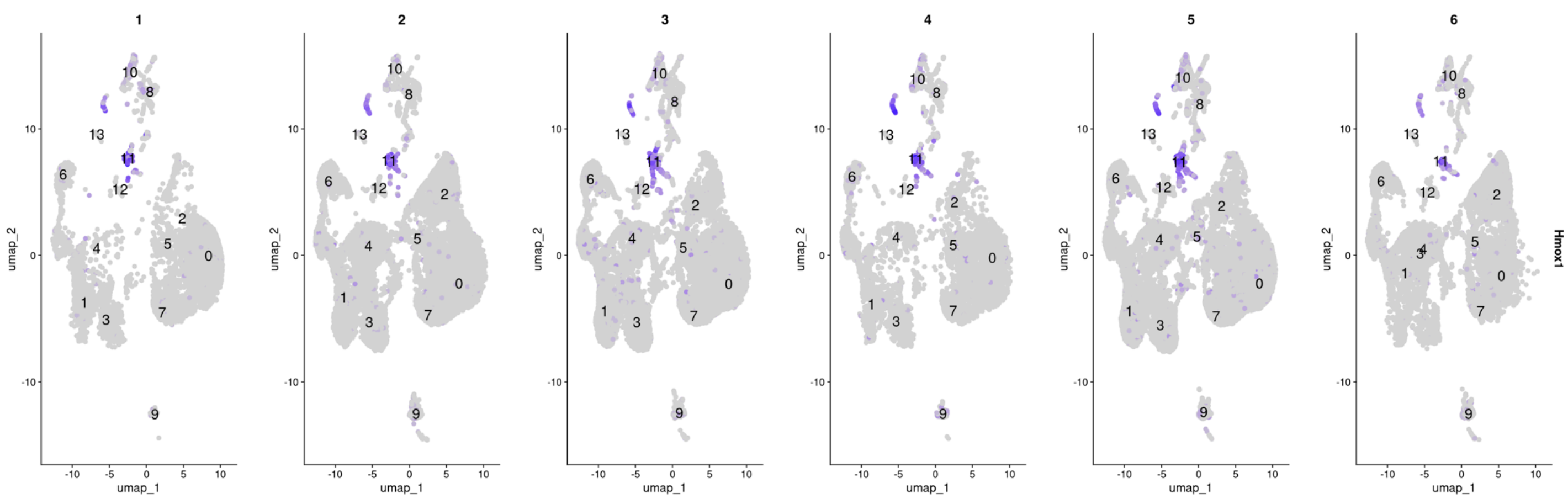

We also recognize the concern regarding the third population observed within the macrophage compartment. We have confirmed that this population represents B cell contamination that was primarily associated with a single scRNA-seq sample in which >50,000 cells were recovered. Inclusion of two additional biological replicates, exclusion of the high-cell-count outlier sample, and more stringent batch correction and integration now demonstrate uniform identification of two distinct macrophage populations, Pde1bpos (cluster 1) and Pde1bneg (cluster 0), across all biological replicates without the contaminating B cell population. These findings are consistent with the integrated pseudobulk analysis. The figure below shows Pde1b expression across individual biological replicates; 3 groups on the left are WT and 3 groups on the right are KO.

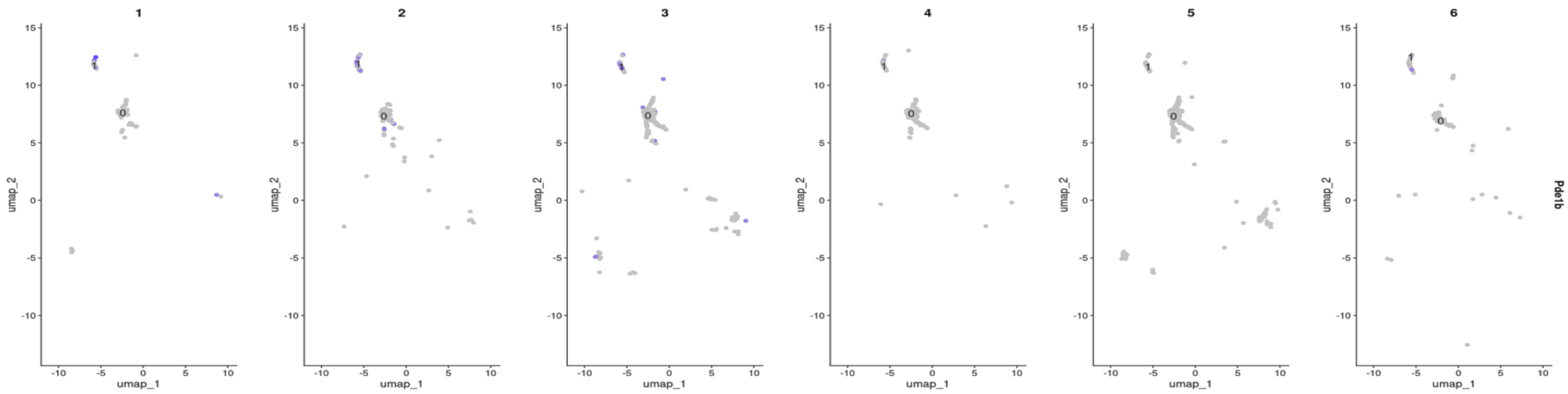

For differential expression analysis of scRNA-seq data, including pseudobulk comparisons, lower logFC thresholds (~0.2–0.25) are employed in combination with statistical significance (adjusted p-values), as is standard practice. This approach enables detection of coordinated but moderate transcriptional shifts across cell populations, which are particularly relevant in immune regulatory contexts. All differential expression analyses were performed using established methodologies with appropriate normalization and statistical testing. Key findings independently validated (i.e. by qPCR or protein expression analyses).

#3 - Reviewer feedback: Whether immunological changes translate into altered disease outcomes in vivo.

- Reviewer #2: "Did the authors observe a more severe disease in Pde1b-/- mice and increased bacterial load in vivo? These data would be important…"
- Reviewer #1:"I wonder if the colonization of liver, gallbladder, bone marrow… was altered" requested microbiological, histopathological, and clinical parameters.

Response: We thank the reviewers for this important question regarding *in vivo* disease severity and tissue dissemination. In this study, we employed the attenuated ΔactA-2W Listeria monocytogenes strain, which is specifically optimized to assess antigen-specific T cell priming and intracellular bacterial control rather than systemic disease progression. We have clarified the procedure in the Methods section, describing that this strain induces a transient infection following intravenous delivery, drives robust expansion of antigen-specific T cells, and is rapidly cleared within a defined experimental window. The actA mutation disrupts actin-based motility and cell-to-cell spread, thereby limiting dissemination beyond initially infected phagocytes. In the context of intravenous administration, this results in preferential localization to blood-filtering secondary lymphoid organs, particularly the spleen, with limited and non-sustained colonization of non-lymphoid tissues (i.e. liver, gallbladder, or bone marrow). To directly address the excellent reviewer question/concern, we do have measurements for spleen bacterial burden now placed into subpanel **Figure 2A**. It is the laboratory standard routine to measure bacterial load among all studies as a direct measure to assure successful *in vivo* tail vein delivery of bacterium, thus we have the quantified bacterial burden following infection among the biological replicates. We observe clear differences between WT vs. KO; KO have comparatively higher bacterial load over this window compared to WT. These data indicate a defect in the transition to the clearance phase, rather than in the initial containment of infection, and are consistent with demonstrating impaired APC function and reduced antigen-specific T cell priming in Pde1b$^{-/-}$ mice.

#4 - Reviewer feedback: Need to distinguish contributions of specific cell types and include rescue-type experiments.

- Reviewer #2 questioned: how to interpret extrinsic effects on lymphocytes and whether DCs/macrophages are the true drivers
- Reviewer #3 stated: "The paper is missing any kind of 'rescue' experiment"

Response: These are both excellent points. We directly assessed cDC1-intrinsic function using an *in vitro* antigen presentation system, thereby eliminating systemic knockout confounders and directly addressing the cell-intrinsic versus extrinsic contribution, as shown in **Figure 3**. To functionally test causality *in vivo*, we transferred sorted WT cDC into Pde1b$^{-/-}$ mice prior to LM infection, as shown in **Figure 4**. This resulted in a restoration of T cell priming in Pde1b$^{-/-}$ hosts, demonstrating that presence of Pde1b in cDC is sufficient to

reconstitute the defective immune response *in vivo*. These findings establish a cell-intrinsic requirement in regulating/directing antigen-specific T cell priming.

#5 - Reviewer feedback: “authors must address… cGMP generators (sGC subunits)… and PKG isoforms”
Response: We present below scRNAseq, validated among the biological replicates and by qPCR, for the following genes expressed in WT vs. KO. For Adenylate Cyclase, we show (Stimulated by Ca^{2+}/Calmodulin): Adcy1, Adcy3, Adcy8, Group 2 (Stimulated by Gβγ): Adcy2, Adcy4, Adcy7, Group 3 (Inhibited by Ca^{2+} or Gαi): Adcy5, Adcy6, Group 4 (Forskolin insensitive): Adcy9. In addition, we show the PKG I and II: Prkg1, Prkg2, and we also assessed in: the PKA Catalytic Subunits: Prkaca, Prkacb, Prkacg and the Regulatory Subunits: Prkar1a, Prkar1b, Prkar2a, Prkar2b. Lastly, we assessed: sGCalpha1, sGCalpha2, sGCbeta1, and NRP1/NRP2. We found no difference between the WT and KO for any of these genes in cDC1 or macrophages. Representative scRNAseq data shown below and the manuscript has been updated with new **Figure 7E** and the discussion section.

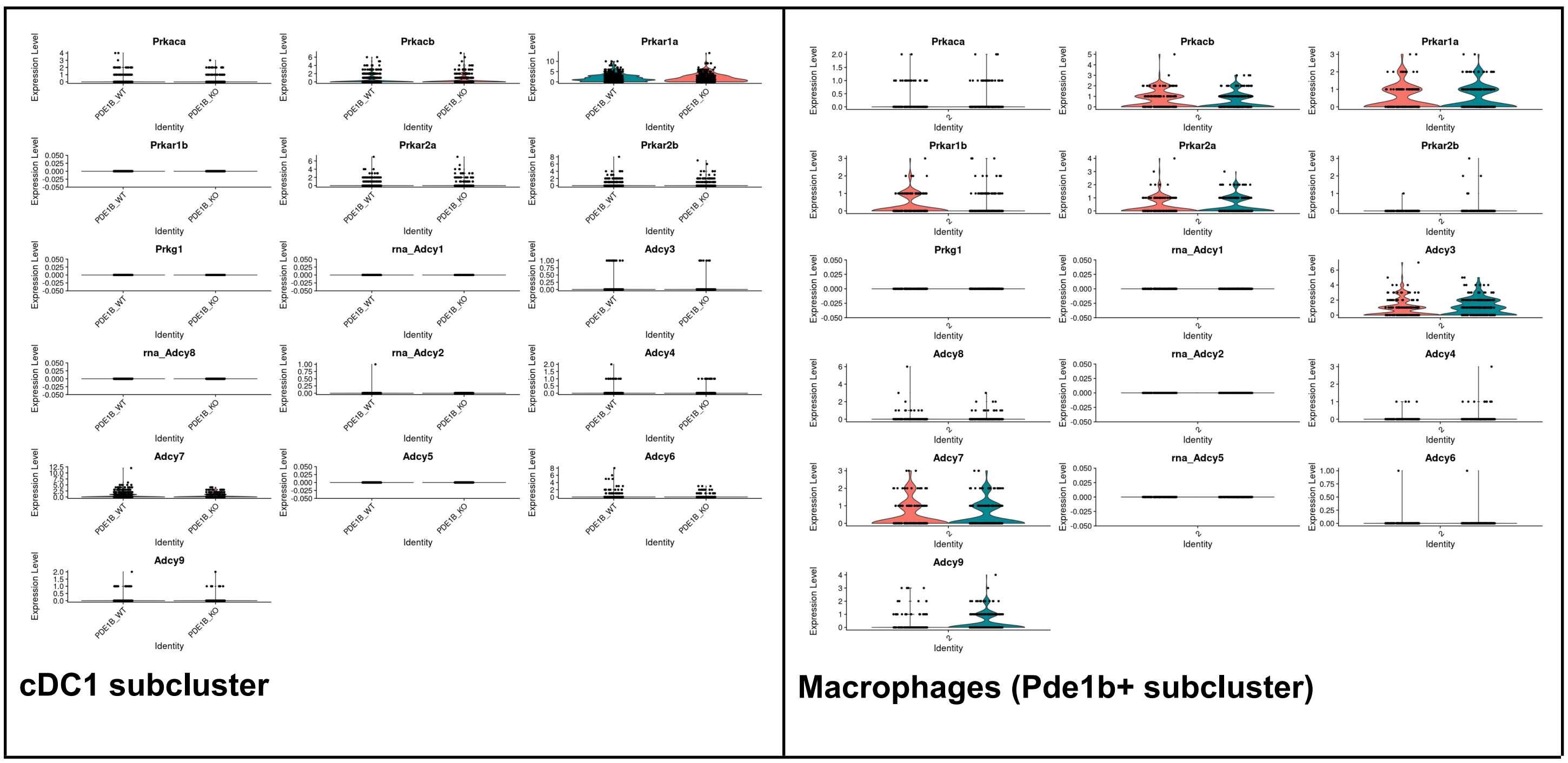

PKG (1a/b) is expressed at very low subset-specific levels in dendritic cells at steady-state, but rapidly induced upon TLR stimulation. The Giordano paper is the most well established linking PKG to regulating more of migratory capacity during inflammatory setting, focusing on 1beta; the most recent work building on this came from Tang et al (Tang, Nature 2025) – both linking to NO, cGMP and trafficking capacity. Below we show the work by Giordano showing increased PKG (Prkg1) upon LPS via qPCR. Our own data of TLR stimulation triggers increased Prkg1, typical of maturing DC prior to priming for in vitro ag-specific T cell priming studies; most of the field leans on this work. To our knowledge, Prkg2 is not expressed nor inducible in mAPC. Although we are still performing and optimizing cGMP-PKG studies for a follow-up story, our developing data indicate that cGMP-PKG is not the dominant regulator of specific T-cell priming in cDC1s, but rather the cAMP-PKA axis. Our current findings strongly support the paradigm established by Giordano et al., wherein the migration switch required for lymph node homing depends on depressed cAMP levels and reciprocal rise in cGMP. However, we posit that this entire cascade first requires cAMP inhibition to be actively relieved. Our working model (still in development stages) wherein the dual-specificity phosphodiesterase Pde1b acts as the principal, bona fide controller of this axis specifically in cDC1. Because of its distinct, differential Km affinities for cAMP versus cGMP, we are testing whether Pde1b dynamically bridges initial cAMP clearance with the subsequent temporal regulation of cGMP by moving into more real time kinetic readouts. This handoff would then coordinate the cyclic nucleotide fluctuations necessary to shuttle cDC1 between a mobile migratory state, where an induction of iNOS (through NO) drives the cGMP surge, and a highly immunogenic state optimized for downstream T-cell instruction upon trafficking to the lymph node.

Figure from Giordano et al, Blood 2006 removed for copyright reasons

References:

- Giordano D, et al Nitric oxide and cGMP protein kinase (cGK) regulate dendritic-cell migration toward the lymph-node-directing chemokine CCL19. Blood. 2006. PMID: 16249377;
- Tang H, Wei Z, Zheng B, Cai Y, Wu P, Wu L, Ma X, Chen Y, Su S, Xu J, Qiao Y, Zhang Y, Miao J, Yu Z, Zhao Y, Xia Z, Zhou R, Liu J, Guo J, Liu Z, Xie Q, Ginhoux F, Zhao L, Li X, Xia B, Wu H, Zhang Y, Zhou T. Rescuing dendritic cell interstitial motility sustains antitumour immunity. Nature. 2025 Sep;645(8079):244-253. doi: 10.1038/s41586-025-09202-9. Epub 2025 Jun 25. PMID: 40562926.

#6 - Reviewer feedback: "The term 'metabolic control'… is not clearly defined and not related to altered metabolic functions like glycolysis and oxidative phosphorylation…"

Response: We agree that the term "metabolic control" is incorrect in this context, and we have removed and revised the manuscript accordingly to better reflect second messenger-driven cyclic nucleotide signaling (cAMP/cGMP, PKA/PKG pathways) rather than classical metabolic flux processes. These changes have been implemented throughout the text to improve conceptual accuracy and clarity.

#7 - Reviewer feedback: "The authors should refine their gating strategies or show isotype or FMO controls…"

Response: We thank the reviewer for this comment. Flow cytometry gating was performed using unstained and single-stained controls for compensation, with exclusion of doublets and debris and restriction to live singlet populations. Gates were defined using appropriate negative cell populations and applied consistently across all datasets. The anti-Pde1b unconjugated antibody was knockout validated (Abcam) and its specificity was further confirmed in-house using Pde1b KO cells compared to WT. We demonstrate the use of isotype vs. Pde1b-antibody in **Figure 1** and use of the phospho-antibodies with respective isotype controls in **Figure 9**. We have updated the Materials and Methods section to more concretely reflect our data acquisition and analysis strategy, including gating and control procedures.

#8 - Reviewer feedback: "Is the expression of Pde1b conserved between mice and humans?"

Response: Yes. Published works and examined publicly available datasets, including resources from the ImmGen Consortium and human protein atlas, demonstrate that Pde1b expression is enriched in human myeloid populations, including dendritic cells and monocytes/macrophages, while remaining low or absent in lymphoid compartments, consistent with our observations in mouse cDC1 and macrophage subsets. Initial publication by Gelinasa et al initially identified and characterized Pde1b in humans, identifying that it was calcium dependent and preferentially targeted cGMP (Yu et al Cell Signal 1997). In subsequent work, Beavo et al used human monocytes obtained from standard blood buffy coats to generate DC under GM-CSF *in vitro* conditions (Bender et al PNAS 2005). They show Pde1b protein expression and mRNA levels in monocytes driven into DC/macrophages. Please see the figure below with accompanying legend from their work.

Figure from Bender et al, PNAS 2005 removed for copyright reasons

References:

- Bender AT, Ostenson CL, Wang EH, Beavo JA. Selective up-regulation of PDE1B2 upon monocyte-to-macrophage differentiation. Proc Natl Acad Sci U S A. 2005 Jan 11;102(2):497-502. doi: 10.1073/pnas.0408535102. Epub 2004 Dec 29. PMID: 15625104; PMCID: PMC544304.
- Yu J, Wolda SL, Frazier AL, Florio VA, Martins TJ, Snyder PB, Harris EA, McCaw KN, Farrell CA, Steiner B, Bentley JK, Beavo JA, Ferguson K, Gelinas R. Identification and characterisation of a human calmodulin-stimulated phosphodiesterase PDE1B1. Cell Signal. 1997 Nov;9(7):519-29. doi: 10.1016/s0898-6568(97)00046-6. PMID: 9419816.

#9 - Reviewer feedback: "Why did the authors perform co-culture of cDC1 and OT-I T cells… they should rather test OT-II T cells."

Responses: We understand the reviewer's comment on this important point, particularly in that we did *in vivo* work with CD4+ T cell responses (*Figure 2*), but then moved next to *in vitro* work with CD8+ T cells (*Figure 3*). The purpose in use of OT-I CD8+ T cells is specifically designed to assess cross-presentation capacity of cell associated antigens, a hallmark functional specialization of cDC1. Additionally, at the time, we did not have available peptide:MHC class I-restricted peptides to measure antigen specific frequency *in vivo* (and hence turned to OT-I model). To now show *in vitro* and *in vivo* relevant comparative data, we performed the rescue studies using WT, Pde1b KO, and Pde1b KO rescued with WT cDC or with Pde1b-deficient cDC using new pMHC:tetramers (obtained from the NIH Tetramer Core Facility) for analyzing LM antigen-specific CD8+ T cell responses *in vivo*. Please see **Figure 4**. As such, we are now able to show *in vitro* and *in vivo* alignment of the important CD8+ T cell responses, with companion support of *in vivo* relevant CD4+ T cell responses.

#10 - Reviewer feedback: "Why did the authors exclude the green-labeled cells in Figure 3C?"

Response: The green-labeled cluster was excluded from macrophage subclustering analyses because it did not express canonical macrophage markers (Itgam, Adgre1) and instead showed enrichment of immunoglobulin and lineage-defining genes (e.g., Pax5), consistent with B cell-like populations. Such overlap can occur in scRNA-seq datasets, where B cells share partial antigen-presenting cell transcriptional features and may be positioned near myeloid clusters during unsupervised clustering. Inclusion of this population would therefore confound macrophage-specific analyses. We have clarified this point in **Figure 6** legend.

#11 - Reviewer feedback: "... reduced frequencies of MDPs. Did the authors observe reduced frequencies of DCs and monocytes/macrophages outside of the bone marrow in Pde1b-/- mice?...

Response: Yes, M-CSF or GM-CSF driven bone marrow differentiation results in lower macrophages in Pde1b-/- vs. WT. The phenotype is strong and reproducible. The bone marrow data has been removed from this revised manuscript due to the inclusion of several requested datasets, narrowing the focus-direction of the manuscript towards Pde1b role in inflammation and immunity, rather than development.

#12 - Reviewer feedback "... directly compare the expression level in cDC1 and macrophages between spleen and thymus? As thymus mainly consists of T cells, which are shown to be negative for Pde1b, this might be misinterpreted".

Response: We appreciate this point and agree that tissue-level comparisons between spleen and thymus can be confounded by differences in cellular composition. However, direct comparison between these tissues is not well suited to addressing the role of Pde1b in APC priming of T cell immunity or inflammation induction. The thymus is predominantly composed of developing lymphoid cells and contains very limited numbers of mature cDC1 and macrophages. Moreover, thymic APC are functionally specialized to support T cell development (e.g., positive and negative selection), rather than peripheral T cell priming. As such, differences in Pde1b expression between thymus and spleen would primarily reflect tissue composition and functional context, rather than provide meaningful insight into its role in peripheral APC immunobiology.

#13 - Author data update

We observed a discrepancy in IL-6 qPCR gene expression profiling vs. that of the Luminex IL-6 protein analyte analyses. To resolve, we repeated using standard IL-6 ELISA using both splenic-derived and bone marrow-derived macrophages and now observe the phenotype across several biological replicates. We have updated the figure subpanel **Figure 3D**. All other datasets were also confirmed accurate using standard individual ELISAs for IL1beta, IL-12p70 and IL-10.

#14 - Minor and Editorial revisions

We have made the following revisions to align the manuscript with the journal styling, including: (a) inclusion of exact p-values, n values, and statistical tests, (b) clarification of figure legends and terminology and (c) inclusion of source data, as well as updated details present within the Materials and Methods section.

Thank you again-

Dear Dr. Lipscomb,

Thank you for the submission of your revised manuscript to our editorial offices. I have now received the reports from the three referees that were asked to re-evaluate the study, you will find below. As you will see, the referees now fully support publication of your study in EMBO reports. Referee #2 has remaining concerns and suggestions to improve the manuscript I ask you to address in a final revised manuscript. Please also provide a final p-b-p-response to the editorial requests and the remaining referee points.

Editorial requests:

- The Expanded View format, which will be displayed in the main HTML of the paper in a collapsible format, has replaced the Supplementary information. Please follow the nomenclature Figure EV1, Figure EV2 etc. The figure legend for these should be included in the main manuscript document file in a section called Expanded View Figure Legends after the main Figure Legends section.

Thus, please upload the 5 supplementary figures as EV Figures, update their callouts (Figure EV1 - Figure EV5) and remove the Appendix file (Supplementary Materials).

- Please order the manuscript sections like this, using only these names:
Title page - Abstract - Keywords (max. 5) - Introduction - Results - Discussion - Methods - Data availability section - Acknowledgements - Disclosure and Competing Interests Statement - References - Figure legends - Expanded View Figure legends

- Please remove the running title form the title page.

- We now use CRediT to specify the contributions of each author in the journal submission system. CRediT replaces the author contribution section. Please use the free text box to provide more detailed descriptions and do NOT provide your final manuscript text file with an author contributions section. See also our guide to authors (section 'Author contributions'): https://link.springer.com/journal/44319/submission-guidelines#cms-Revised-submissions

- Please move the parapgrah 'Declaration of generative AI and AI-assisted technologies in the writing process' to the methofd section.

- Please check again that the number "n" for how many independent experiments were performed, their nature (biological versus technical replicates), the bars and error bars (e.g. SEM, SD) and the test used to calculate p-values is indicated in the respective figure legends (main and EV figures). Please also check that all the p-values are explained in the legend, and that these fit to those shown in the figure. Please provide statistical testing where applicable. Please avoid the phrase 'independent experiment' but clearly state if these were biological or technical replicates. Please also indicate (e.g. with n.s.) if testing was performed, but the differences are not significant. In case n=2, please show the data as separate datapoints without error bars and statistics. See also:

https://link.springer.com/journal/44319/submission-guidelines#cms-Figure-and-data-presentation

If n<5, please show single datapoints for diagrams. Moreover:
- Please note that the exact p values are not provided in the legend of figure 8C
- Please note that information related to n is missing in the legends of figures 1E, F; 3F, 5A, 6A
- Please note that the error bars are not defined in the legends of figures 1E, F.

- The data availability section (DAS) is restricted to information about large datasets generated in the study that have been deposited externally. Please remove any other information from this section.

In addition, I would need from you uploaded separately:
- a short, two-sentence summary of the manuscript (not more than 35 words).
- two to four short (!) bullet points highlighting the key findings of your study (two lines each).
- a schematic summary figure as separate file that provides a sketch of the major findings (not a data image) in jpeg or tiff format (with the exact width of 550 pixels and the exact height of 300 pixels) that can be used as a visual synopsis on our website. Please upload this separately and remove the graphical abstract from the manuscript text file.

I look forward to seeing the further revised version of your manuscript when it is ready. Please let me know if you have questions regarding the revision.

Best,

Achim Breiling
Senior Editor
EMBO Reports

Referee #1:

The authors have significantly revised their compelling study. Most of my main points were addressed, some of them even experimentally. I have no further concerns regarding this manuscript.

Referee #2:

The authors adequately adressed most of my concerns. Some minor points remain that might be adressed:
1) As the data with the rescue experiment with cDC1 shows still an effect when wt cDC1 are transfered, the authors should add as a limitation of their study that in the mice in their rescue experiment contain both host Pde1b- cDC1 and donor wt Pde1b+ cDC1 and that this might cause a reduction in CD8+ T cell activation. Here, use of Batf3-/- mice as host would have been more adequate to exclude a mixture of Pde1b- and Pde1b+ cDC1.
2) In supplemental figure 2, the authors show lineage defining transcription factors for their scRNAseq DC clustering. While XCR1 looks convincing that cluster 1 contains cDC1, there is no difference between cluster 0 and cluster 1 in expression of Batf3 and Irf4, which should clearly distinguish on mRNA level between cDC1 and cDC2, respectively. The authors might add some additional cDC2 specific molecules as Sirpa and Itgam are broadly expressed among myeloid cells and not specific for cDC2.

Referee #3:

The authors have addressed all my comments satisfactorily.

Response to Reviewers

We thank the Editor and reviewers for their positive evaluation and constructive feedback, which greatly strengthened our manuscript. Our responses and revisions address the concerns on limitations of the rescue experiments and better clarify the use of markers for cDC/pDC subset identification. Below, we provide a point-by-point response addressing all remaining points.

#1 - Reviewer feedback: *As the data with the rescue experiment with cDC1 shows still an effect when wt cDC1 are transferred, the authors should add as a limitation of their study that in the mice in their rescue experiment contain both host Pde1b- cDC1 and donor wt Pde1b+ cDC1 and that this might cause a reduction in CD8+ T cell activation. Here, use of Batf3-/- mice as host would have been more adequate to exclude a mixture of Pde1b- and Pde1b+ cDC1.*

Response: This is a very good suggestion; it is indeed a caveat. We have updated the discussion section to input this point. The inserted section now reads: "As a potential limitation, recipient mice harbor endogenous Pde1b-deficient cDC1 alongside transferred wild-type cDC1. This cellular co-existence introduces the possibility of bidirectional crosstalk influencing effector states or direct competition for antigen and cognate T cells during priming. Although the results show partial restoration, in the absence of a cDC1-deficient host model, we cannot conclusively rule out that endogenous mutant cDC1 actively impede or outcompete adoptively transferred cells. Furthermore, because macrophage populations remain Pde1b-deficient, these experiments cannot definitively establish whether cDC1 restoration alone is fully sufficient for maximal effector T cell responses without concurrent correction across cooperating myeloid compartments."

#2 - Reviewer feedback: "*In supplemental figure 2, the authors show lineage defining transcription factors for their scRNAseq DC clustering. While XCR1 looks convincing that cluster 1 contains cDC1, there is no difference between cluster 0 and cluster 1 in expression of Batf3 and Irf4, which should clearly distinguish on mRNA level between cDC1 and cDC2, respectively. The authors might add some additional cDC2 specific molecules as Sirpa and Itgam are broadly expressed among myeloid cells and not specific for cDC2."*

Response: We thank the referee for the careful evaluation and recognize now the ambiguity in our initial manuscript text, where Batf3 was imprecisely framed as a strict binary marker for cDC1. To correct/clarify this in the manuscript, we have revised our text to read that Batf3 and Zbtb46 mark the broader classical DC (cDC) lineage rather than distinguishing cDC1 from cDC2. Furthermore, cluster segregation and subset identities are rigorously defined using our combinatorial marker panel: cDC1 cells are identified by Xcr1, Irf8, Aif1, Cd8a, and Cd24; cDC2 cells by Itgam (CD11b), Irf4 and Sirpa (CD172a)*; and pDCs by Siglech and Irf8 (as presented in Expanded View Figure 2). Importantly, ImmGen data does align directly with our scRNAseq data to corroboratively reveal that Batf3 and Irf4 do not serve as absolute binary separators between cDC1 and cDC2 populations at the transcript level (please see below). Specifically, while Irf4 exhibits relatively low relative expression in cDC1s, it is prominently enriched in cDC2, and Batf3 is similarly shared across classical DC subsets with preferential enrichment in cDC1.

Thank you,

-Authors

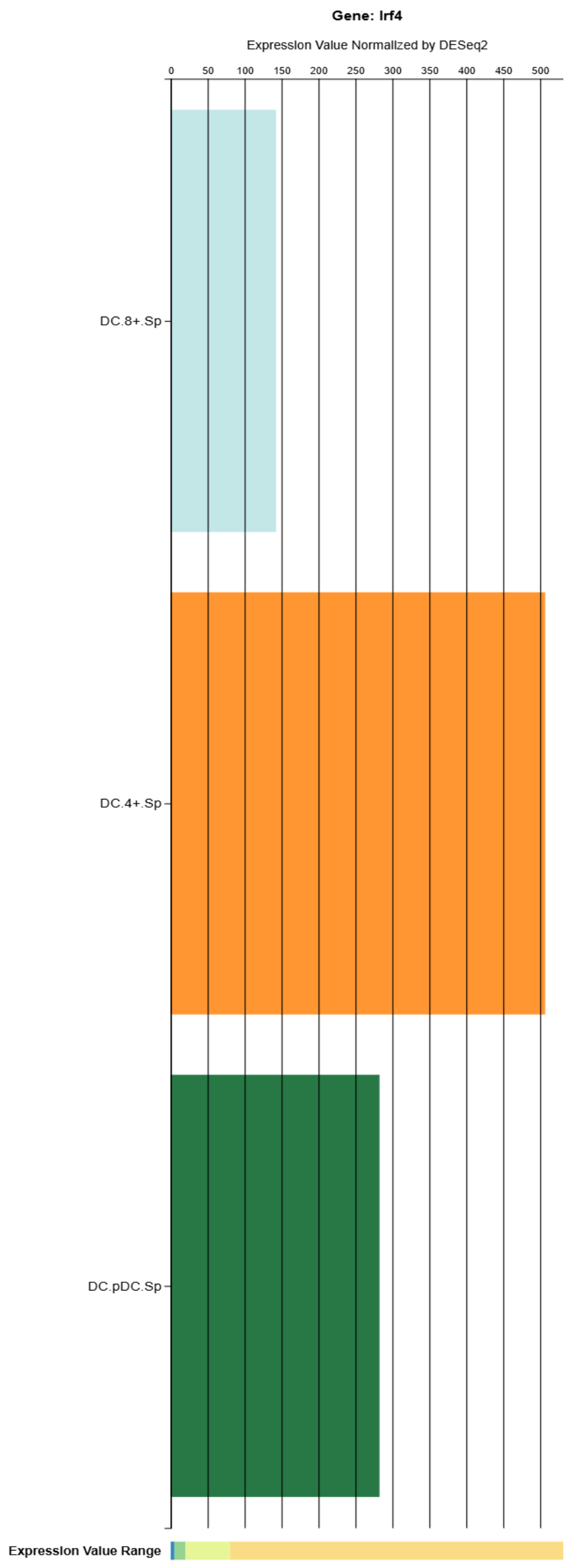	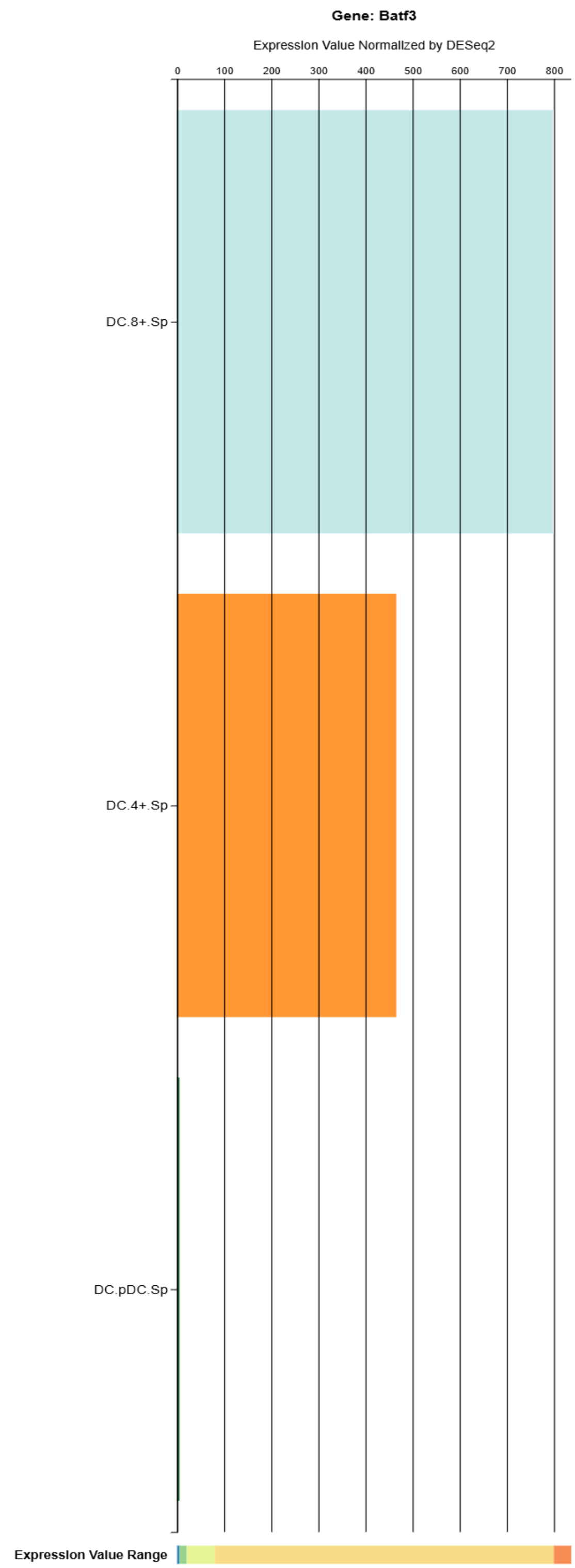
IRF4 expression in splenic cDC1 (DC.8+Sp), cDC2 (DC.4+.Sp), and pDC (DC.pDC.Sp). Expression is prominent in cDC2, but also expressed in cDC1 and cDC2	Batf3expression in splenic cDC1 (DC.8+Sp), cDC2 (DC.4+.Sp), and pDC (DC.pDC.Sp). Expression is prominent in cDC1, but also expressed in cDC2 and not in pDC

Dr. Michael Lipscomb
University of Minnesota
Pharmacology
United States

Dear Dr. Lipscomb,

I am very pleased to accept your manuscript for publication in the next available issue of EMBO reports. Thank you for your contribution to our journal.

Your manuscript will be processed for publication by EMBO Press. It will be copy edited and you will receive page proofs prior to publication. Please note that you will be contacted by Springer Nature Author Services to complete licensing and payment information.

You may qualify for financial assistance for your publication charges - either via a Springer Nature fully open access agreement or an EMBO initiative. Check your eligibility: https://link.springer.com/journal/44319/how-to-publish-with-us

Should you be planning a Press Release on your article, please notify the production team via this form in order to coordinate publication and release dates: https://forms.cloud.microsoft/e/eH4A8JVP6g

If you have any questions, please do not hesitate to contact the Editorial Office. Thank you for your contribution to EMBO Reports.

Yours sincerely,

Martina Rembold, PhD
Senior Editor
EMBO reports

>>> Please note that it is EMBO Reports policy for the transcript of the editorial process (containing referee reports and your response letter) to be published as an online supplement to each paper. If you do NOT want this, you will need to inform the Editorial Office via email immediately. More information is available here: https://link.springer.com/partners/embo-press/editorial-policies#Peer%20review

EMBO Press Author Checklist

Corresponding Author Name: Michael Lipscomb	
Journal Submitted to: EMBO Reports	
Manuscript Number: EMBOR-2025-62733V2	

USEFUL LINKS FOR COMPLETING THIS FORM
The EMBO Journal - Author Guidelines
EMBO Reports - Author Guidelines
Molecular Systems Biology - Author Guidelines
EMBO Molecular Medicine - Author Guidelines

Reporting Checklist for Life Science Articles (updated January
This checklist is adapted from Materials Design Analysis Reporting (MDAR) Checklist for Authors. MDAR establishes a minimum set of requirements in transparent reporting in the life sciences (see Statement of Task: 10.31222/osf.io/9sm4x). Please follow the journal's guidelines in preparing your manuscript.
Please note that a copy of this checklist will be published alongside your article.

Abridged guidelines for figures
1. Data
The data shown in figures should satisfy the following conditions:
- the data were obtained and processed according to the field's best practice and are presented to reflect the results of the experiments in an accurate and unbiased manner.
- ideally, figure panels should include only measurements that are directly comparable to each other and obtained with the same assay.
- plots include clearly labeled error bars for independent experiments and sample sizes. Unless justified, error bars should not be shown for technical
- if n<5, the individual data points from each experiment should be plotted. Any statistical test employed should be justified.
- Source Data should be included to report the data underlying figures according to the guidelines set out in the authorship guidelines on Data

2. Captions
Each figure caption should contain the following information, for each panel where they are relevant:
- a specification of the experimental system investigated (eg cell line, species name).
- the assay(s) and method(s) used to carry out the reported observations and measurements.
- an explicit mention of the biological and chemical entity(ies) that are being measured.
- an explicit mention of the biological and chemical entity(ies) that are altered/varied/perturbed in a controlled manner.
- the exact sample size (n) for each experimental group/condition, given as a number, not a range;
- a description of the sample collection allowing the reader to understand whether the samples represent technical or biological replicates (including how many animals, litters, cultures, etc.).
- a statement of how many times the experiment shown was independently replicated in the laboratory.
- definitions of statistical methods and measures:

- common tests, such as t-test (please specify whether paired vs. unpaired), simple χ2 tests, Wilcoxon and Mann-Whitney tests, can be unambiguously identified by name only, but more complex techniques should be described in the methods section;

- are tests one-sided or two-sided?
- are there adjustments for multiple comparisons?
- exact statistical test results, e.g., P values = x but not P values < x;
- definition of 'center values' as median or average;
- definition of error bars as s.d. or s.e.m.

Please complete ALL of the questions below.
Select "Not Applicable" only when the requested information is not relevant for your study.

Materials

Newly Created Materials	Information included in the manuscript?	In which section is the information available? (Reagents and Tools Table, Materials and Methods, Figures, Data Availability Section)
New materials and reagents need to be available; do any restrictions apply?	Not Applicable	

Antibodies	Information included in the manuscript?	In which section is the information available? (Reagents and Tools Table, Materials and Methods, Figures, Data Availability Section)
For **antibodies** provide the following information:<br>- Commercial antibodies: RRID (if possible) or supplier name, catalogue number and or/clone number<br>- Non-commercial: RRID or citation	Yes	Reagents and Tools table; Materials and Methods

DNA and RNA sequences	Information included in the manuscript?	In which section is the information available? (Reagents and Tools Table, Materials and Methods, Figures, Data Availability Section)
**Short novel DNA or RNA including primers, probes**: provide the sequences.	Yes	Reagents and Tools table; Materials and Methods

Cell materials	Information included in the manuscript?	In which section is the information available? (Reagents and Tools Table, Materials and Methods, Figures, Data Availability Section)
**Cell lines:** Provide species information, strain. Provide accession number in repository **OR** supplier name, catalog number, clone number, and/**OR** RRID.	Not Applicable	
**Primary cultures:** Provide species, strain, sex of origin, genetic modification status.	Yes	Materials and Methods, Figures
Report if the cell lines were recently **authenticated** (e.g., by STR profiling) and tested for mycoplasma contamination.	Not Applicable	

Experimental animals	Information included in the manuscript?	In which section is the information available? (Reagents and Tools Table, Materials and Methods, Figures, Data Availability Section)
**Laboratory animals or Model organisms:** Provide species, strain, sex, age, genetic modification status. Provide accession number in repository **OR** supplier name, catalog number, clone number, **OR** RRID.	Yes	Reagents and Tools Table, Materials and Methods, Figures
**Animal observed in or captured from the field:** Provide species, sex, and age where possible.	Not Applicable	
Please detail **housing and husbandry conditions**.	Yes	Materials and Methods

Plants and microbes	Information included in the manuscript?	In which section is the information available? (Reagents and Tools Table, Materials and Methods, Figures, Data Availability Section)
**Plants:** provide species and strain, ecotype and cultivar where relevant, unique accession number if available, and source (including location for collected wild specimens).	Not Applicable	
**Microbes:** provide species and strain, unique accession number if available, and source.	Not Applicable	

Human research participants	Information included in the manuscript?	In which section is the information available? (Reagents and Tools Table, Materials and Methods, Figures, Data Availability Section)
If collected and within the bounds of privacy constraints report on age, sex and gender or ethnicity for all study participants.	Not Applicable	

Core facilities	Information included in the manuscript?	In which section is the information available? (Reagents and Tools Table, Materials and Methods, Figures, Data Availability Section)
If your work benefited from core facilities, was their service mentioned in the acknowledgments section?	Yes	

Materials and Methods, Acknowledgements

Design

Study protocol	Information included in the manuscript?	In which section is the information available? (Reagents and Tools Table, Materials and Methods, Figures, Data Availability Section)
If study protocol has been **pre-registered, provide DOI in the manuscript**. For clinical trials, provide the trial registration number **OR** cite DOI.	Not Applicable	
Report the **clinical trial registration number** (at ClinicalTrials.gov or equivalent), where applicable.	Not Applicable	

Laboratory protocol	Information included in the manuscript?	In which section is the information available? (Reagents and Tools Table, Materials and Methods, Figures, Data Availability Section)
Provide DOI OR other citation details if **external detailed step-by-step protocols** are available.	Not Applicable	

Experimental study design and statistics	Information included in the manuscript?	In which section is the information available? (Reagents and Tools Table, Materials and Methods, Figures, Data Availability Section)
Include a statement about **sample size** estimate even if no statistical methods were used.	Yes	Materials and Methods
Were any steps taken to minimize the effects of subjective bias when allocating animals/samples to treatment (e.g. **randomization procedure**)? If yes, have they been described?	Yes	Materials and Methods
Include a statement about **blinding** even if no blinding was done.	Yes	Materials and Methods
Describe **inclusion/exclusion criteria** if samples or animals were excluded from the analysis. Were the criteria pre-established?<br><br>If sample or data points were omitted from analysis, report if this was due to attrition or intentional exclusion and provide justification.	Yes	Materials and Methods
For every figure, are **statistical tests** justified as appropriate? Do the data meet the assumptions of the tests (e.g., normal distribution)? Describe any methods used to assess it. Is there an estimate of variation within each group of data? Is the variance similar between the groups that are being statistically compared?	Yes	Materials and Methods

Sample definition and in-laboratory replication	Information included in the manuscript?	In which section is the information available? (Reagents and Tools Table, Materials and Methods, Figures, Data Availability Section)
In the figure legends: state number of times the experiment was **replicated** in laboratory.	Yes	Figure Legends
In the figure legends: define whether data describe **technical or biological replicates**.	Yes	Figure Legends

Ethics

Ethics	Information included in the manuscript?	In which section is the information available? (Reagents and Tools Table, Materials and Methods, Figures, Data Availability Section)
Studies involving **human participants**: State details of **authority granting ethics approval** (IRB or equivalent committee(s), provide reference number for approval.	Not Applicable	
Studies involving **human participants**: Include a statement confirming that **informed consent** was obtained from all subjects and that the experiments conformed to the principles set out in the WMA Declaration of Helsinki and	Not Applicable	
Studies involving **human participants:** For publication of **patient photos**, include a statement confirming that consent to publish was obtained.	Not Applicable	
Studies involving experimental **animals**: State details of **authority granting ethics approva**l (IRB or equivalent committee(s), provide reference number for approval. Include a statement of compliance with ethical regulations.	Yes	Materials and Method
Studies involving **specimen and field samples:** State if relevant **permits** obtained, provide details of authority approving study; if none were required, explain why.	Not Applicable	

Dual Use Research of Concern (DURC)	Information included in the manuscript?	In which section is the information available? (Reagents and Tools Table, Materials and Methods, Figures, Data Availability Section)
Could your study fall under dual use research restrictions? Please check biosecurity documents and list of **select agents and toxins** (CDC): https://www.selectagents.gov/sat/list.htm	Not Applicable	
If you used a select agent, is the security level of the lab appropriate and reported in the manuscript?	Not Applicable	
If a study is subject to dual use research of concern regulations, is the name of the **authority granting approval and reference number** for the regulatory approval provided in the manuscript?	Not Applicable	

Reporting

The MDAR framework recommends adoption of discipline-specific guidelines, established and endorsed through community initiatives. Journals have their own policy about requiring specific guidelines and recommendations to complement MDAR.

Adherence to community standards	Information included in the manuscript?	In which section is the information available? (Reagents and Tools Table, Materials and Methods, Figures, Data Availability Section)
State if relevant guidelines or checklists (e.g., **ICMJE, MIBBI, ARRIVE, PRISMA**) have been followed or provided.	Yes	
For **tumor marker prognostic studies,** we recommend that you follow the **REMARK** reporting guidelines (see link list at top right). See author guidelines, under 'Reporting Guidelines'. Please confirm you have followed these guidelines.	Not Applicable	
For **phase II and III randomized controlled trials**, please refer to the **CONSORT** flow diagram (see link list at top right) and submit the CONSORT checklist (see link list at top right) with your submission. See author guidelines, under 'Reporting Guidelines'. Please confirm you have submitted this list.	Not Applicable	

Data Availability

Data availability	Information included in the manuscript?	In which section is the information available? (Reagents and Tools Table, Materials and Methods, Figures, Data Availability Section)
Have **primary datasets** been deposited according to the journal's guidelines (see 'Data Deposition' section) and the respective accession numbers provided in the Data Availability Section?	Yes	Materials and Methods
Were **human clinical and genomic datasets** deposited in a public access-controlled repository in accordance to ethical obligations to the patients and to the applicable consent agreement?	Not Applicable	
Are **computational models** that are central and integral to a study available without restrictions in a machine-readable form? Were the relevant accession numbers or links provided?	Not Applicable	
If publicly available data were reused, provide the respective **data citations in the reference list**.	Not Applicable	
